# Synthesis and biological evaluation of new brassinosteroid analogs with C-22 benzoate function

**DOI:** 10.3762/bjoc.22.57

**Published:** 2026-05-18

**Authors:** María Núñez, Camila Escobar, Mario Párraga, Mauricio Soto, Luis Espinoza-Catalán, Katy Díaz, Andrés F Olea

**Affiliations:** 1 Departamento de Química, Universidad Técnica Federico Santa María, Avenida España 1680, Valparaíso 2340000, Chile; 2 Centro Interdisciplinario de Investigación Biomédica e Ingeniería para la Salud, MEDING. Laboratorio de Biología molecular. Escuela de Medicina, Universidad de Valparaíso, Chile; 3 Instituto de Ciencias Aplicadas, Facultad de Ingeniería, Universidad Autónoma de Chile, Av. del Valle Sur 534, Santiago 8580640, Chile

**Keywords:** biological activity, brassinosteroids, BRI1-EMS-Suppressor1, ligand–receptor interaction, structure–activity relationship

## Abstract

The synthesis and structural characterization of new brassinosteroid (BR) analogs, in which substituents with different electronegativities and molecular sizes have been attached to a C-22 benzoate function, are described. The biological activities of all new compounds were evaluated by using the rice lamina inclination test (RLIT) and inhibition of root growth of *Arabidopsis thaliana*. The RLIT data is compared with those previously reported for two series of compounds having the same substitution pattern at C-22 but different structure in ring A. This comparison revealed that a 2α,3α-dihydroxy configuration is more active than a 3-carbonyl or 3β-hydroxy function in this ring. Additionally, the accumulation of the dephosphorylated form of the BES1 protein, which is part of the BRs signaling pathway and control their activity, has been evaluated as well. The results are analyzed in terms of BR analog’s structure and compared with binding energies obtained from a docking study. In this way, it is intended to assess the effect of chemical structure on the initial and one intermediate step, and on the final plant response. Our results show that the binding of BR analogs to the active site, which initiate the signaling process, and dephosphorylation of BES1 depend on the structure of BR analogs in a similar way. However, the relationship between the BR analog’s structure and the final plant response is different. The dependence on unknown factors which are able to activate or repress genes associated with growth and development is discussed.

## Introduction

**Figure 1:**
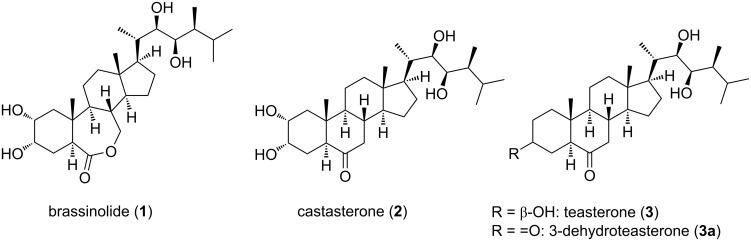
Structures of natural occurring brassinosteroids.

Brassinosteroids (BRs) are phytohormones that are widespread in the plant kingdom, and are found in very low concentrations, i.e., in the of nano- to micromolar range [1–4]. BRs play various important roles, such as, cell development, vascular differentiation, reproduction, modulation of gene expression [5–6], and other developmental processes [7–8]. Their natural occurrence and biological activities have been reviewed by different groups, and brassinolide (**1**) and castasterone (**2**) are the most active and widely distributed [4,9–10], whereas teasterone (**3**) and 3-dehydroteasterone (**3a**), which are intermediates in the biosynthetic pathway, exhibit much lower activities [11–14] (Figure 1).

It has been established that BRs are sensed by the extracellular part of a receptor kinase, NRI1, and then the activated BRI1 initiates a series of processes comprising the transduction pathway of BRs [3,15]. The main consequence of this mechanism is the accumulation of two transcription factors: BZR1 (Brassinazole Resistant1) and BES1 (BRI1-EMS-Suppressor1) in the nucleus, which regulates the expression of a large number of genes involved in different physiological processes in *Arabidopsis thaliana* [15–17]. At the same time, BZR1 and BES1 repress thousands of genes associated to BRs biosynthesis, helping to maintain BRs homeostasis [18]. As consequence, BRs responses are inhibited.

Various bioassays have been developed to evaluate the effects of different concentrations of exogenous application of BRs on plant growth. The most widely used are bean second internode bioassay (BSIB ) [19], rice lamina inclination test (RLIT) [20–21], and inhibition of root and hypocotyl elongation in *Arabidopsis thaliana* seedlings, among other bioassays. The results show that for the same BRs, the measured effects vary with the bioassay and concentration. For example, in the RLIT the growing effect increases with increasing concentration, whereas on root growth BRs act as root growth promoter at low concentration and the opposite effect is observed at high concentrations [22].

As sensing of BRs by BRI1 is the starting point of the mechanism by which plant responses are produced, this binding process has been extensively studied and it has been shown that bioactivity can be correlated with the binding of BRs to BRI1 [23–24], and some structure–activity relationships (SAR) have been proposed [25–26].

**Figure 2:**
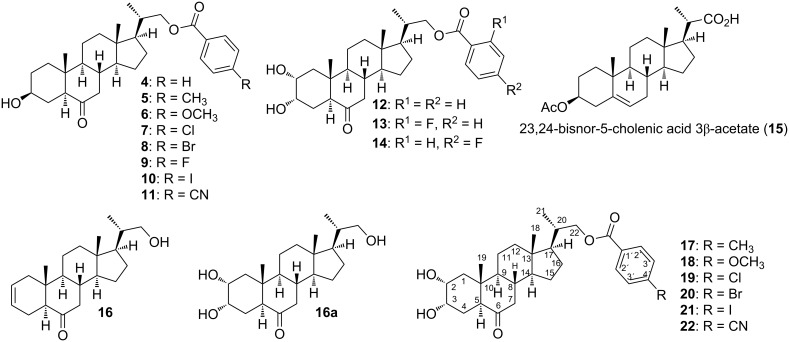
Structures of C-22 benzoate-functionalized brassinosteroid analogs **4**–**14** and **17**–**22** prepared from the known precursor **16** [29], analog **16a** [29] and structure of starting material 23,24-bisnor-5-cholenic acid 3β-acetate (**15**).

The low abundance of BRs present in plants has prompted the quest of synthetic pathways to obtain these compounds. However, to synthesize molecules with the same stereochemical configuration of natural BRs, both in the rings and side alkyl chain, is an enormous synthetic challenge [27]. Consequently, synthetic BRs and BR analogs are not affordable for large-scale commercial applications and therefore, the search for more accessible and bioactive analogs has become a matter of current interest. For example, derivatives of teasterone (**3**), compounds **4**–**11**, and castasterone, compounds **12**–**14**, with benzoyl function at C-22 (Figure 2) have been synthesized, and their bioactivities have been evaluated by BSIB, RLIT, and inhibition of root and hypocotyl elongation in *A. thaliana* seedlings [28–29].

The obtained results indicate that compounds **10** and **11** are the most active of teasterone analogs [28], whereas fluorinated castasterone analogs **13** and **14** with a fluoro substituent in the *ortho-* and *para-*positions, show interesting activities at intermediate concentrations [29].

Based on the preliminary and promissing results obtained for castasterone analogs **13** and **14**, it was decided to expand this study by considering the 2α,3α diol function in ring A, a ketone function at C-6, and C-22 benzoate-function with various substituents, of different electronegativities and sizes in the aromatic ring. In this way, we intend to assess the effect of these properties on the biological activity. Additionally, by comparing RLIT results obtained for this new series of castasterone (**2**) with those obtained for teasterone (**3**) [30] and 3-dehydroteasterone (3-DT, **3a**) [31] analogs, it would be possible to evaluate the contribution of different functions attached to ring A. Thus, herein the synthesis of analogs **17**–**22**, starting from the precursor **16**, is described. All compounds have been fully characterized by spectroscopic techniques (IR, 1D, 2D NMR and HRMS).

The biological activities have been assessed by RLIT and inhibition of root growth of *A. thaliana*. Additionally, the Western blot technique has been applied for the detection and identification of BES1 in *A. thaliana* seedlings, which has been used as a model organism for this type of study [32].

## Results and Discussion

### Chemistry

**Scheme 1:**
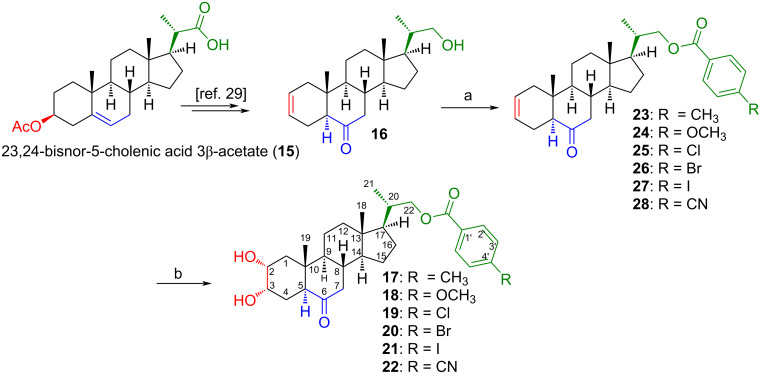
Synthesis of compounds **23**–**28** and new BR analogs **17**–**22**, from 23,24-bisnor-5-cholenic acid 3β-acetate (**15**). Conditions: a) *p*-R-PhCOCl/CH_2_Cl_2_/py, DMAP, rt, 2 h, 96.7%, 78.0%, 91.2%, 95.3%, 95.6%, and 93.6% yields for **23**–**28**, respectively. b) DHQD-CLB, CH_3_SO_2_NH_2_, K_2_CO_3_, K_3_[Fe(CN)_6_], OsO_4_, *t*-BuOH/H_2_O (1:1 v/v), rt, 36 h, 77.8%, 67.9%, 65.8%, 84.9%, 77.5%, and 63.4% yields for **17**–**22**, respectively. The overall yields for compounds **17**–**22** are 75.2%, 53.0%, 60.0%, 80.9%, 74.1%, and 59.3%, respectively.

The synthesis of new BR analogs **17**–**22** (Figure 2) and compounds **23**–**28** has been carried out using the olefinic precursor **16** according to previously described procedures (Scheme 1) [28–29].

Treatment of olefine **16** with *p*-R-PhCOCl in DMAP/py/CH_2_Cl_2_, leads to compounds **23**–**28** with high yields (78.0–96.7%). All compounds have been characterized by IR, NMR (1D and 2D) and HRMS spectroscopy techniques (see Supporting Information File 1, Figures S1–S30). Thus, in the ^1^H NMR spectrum of compound **23**, the presence of signals at δ = 7.91 ppm (2H, d, *J* = 8.1 Hz) and 7.21 ppm (2H, d, *J* = 8.1 Hz) are assigned to aromatic hydrogens H-2’ and H-3’, respectively. While the observed signal at δ = 2.38 ppm (3H, s) corresponds to CH_3_-Ar. Additionally, the signals observed downfield (by the electron-withdrawing effect of the ester function) at δ = 4.27 (1H, dd, *J* = 10.8 and 3.4 Hz, H-22a) and 4.01 (1H, dd, *J* = 10.8 and 7.2 Hz, H-22b), are assigned to carbinolic hydrogens H-22a and H-22b, respectively. In the ^13^C NMR spectrum, the signals observed at δ = 166.57, 143.31, 129.40, 128.93, 127.65, and 21.52 ppm, are assigned to carbon ArCOO, C-4’, C-2’, C-3’, C-1’, and CH_3_-Ar of the benzoate group, respectively. All these signals show direct correlation (^1^*J*_HC_ coupling) in the 2D HSQC and ^2^*J*_HC_ or ^3^*J*_HC_ on the 2D HMBC spectra (Figures S1–S5, Supporting Information File 1). The structural determination of compounds **24**–**28** has been performed in a similar manner to that used for compound **23**.

Next, Sharpless dihydroxylation of double bond on C-2 of compounds **23**–**28**, following a described procedure [29], leads to BR analogs **17**–**22** with good reaction yields (63.4–84.9%) (Scheme 1).

Structural characterization of BR analogs **17**–**22** was accomplished by using ^13^C NMR data, combined with 2D NMR techniques (HSQC and HMBC). For example, in the ^1^H NMR spectrum of compound **17**, the three signals appearing at δ = 4.26 (1H, dd, *J* = 10.4 and 3.4 Hz), 4.02–3.97 (2H, m), and 3.71 (1H, bd, *J* = 10.2 Hz) are assigned to carbinolic hydrogens H-22a, H-2, H-22b, and H-3, respectively (Figure S32, Supporting Information File 1). While from the ^13^C NMR spectrum, the signals observed at δ = 69.65, 68.38, and 68.27, are assigned to carbinolic carbons C-22, C-3, and C-2, respectively (Figure S33, Supporting Information File 1). In this way, the carbinol signals of H and C were confirmed by correlations to ^1^*J*_HC_, ^2^*J*_HC_, and ^3^*J*_HC_ from the ^1^H,^13^C HSQC NMR and ^1^H,^13^C HMBC NMR spectra data, respectively (Figures S35 and S36, Supporting Information File 1).

Previous studies have shown that Sharpless dihydroxylation of 2,3-steroidal alkenes with OsO_4_/DHQD-CLB system, leads to formation of "*cis*" glycols with C-2(*R*) and C-3(*S*) stereochemistry in the A ring of A/B *trans*-fused (5α steroids). This is the consequence of the steric hindrance imposed by the 19-methyl group, which prevents OsO_4_ from approaching the top face of the A ring, and thereby favoring the reaction from the lower face of the double bond [29,33–34].

The structural determination of compounds **18**–**22** has been performed in a similar way to that used to characterize compound **17** (Figures S37–S66, Supporting Information File 1). In summary, BRs analogs **17**–**22** have been efficiently synthesized in a two-step pathway starting from **16**. The overall yields for **17**–**22** are 75.2%, 53.0%, 60.0%, 80.9%, 74.1%, and 59.3%, respectively.

### Biological activity

#### Rice lamina inclination test (RLIT)

RLIT is a standard, sensitive, and specific method widely used to evaluate BRs activity [20–21]. BRs induce a greater cell expansion of adaxial cells relative to abaxial cells in the attachment regions, causing laminar inclination in a concentration-dependent manner. Although the molecular mechanism of this phenomenon remains elusive [35–36], this bioassay has been used with a large number of BR analogs and the obtained results have allowed the establishment of structure–activity relationships [37–38]. Interestingly, the interpretation of the obtained data is commonly carried out in terms of the binding energy of BRs analogs to the active site of *A. thaliana*, which is calculated from molecular docking studies [28].

Herein, the difference of bending angles induced by analogs **12**, **14** and **17**–**22** have been measured at different concentrations (Table S1, Supporting Information File 1). The activities, calculated as the difference of bending angles obtained for the analogs and the negative control, and normalized respect to the latter, are shown in Table 1.

**Table 1:** RLIT activities of BR analogs of 2α,3α-dihydroxy-5α-cholan-6-oxo-23,24-dinor-22-(4-substituted)-benzoate-22-yl type, on seedlings after 72 h of incubation.

RLIT activities ± (standard error)^a^			

Compounds	1 × 10^−8^ M	1 × 10^−7^ M	1 × 10^−6^ M

**1**	4.58 ± 1.33	6.25 ± 1.92	7.83 ± 2.03
**12**	4.50 ± 1.67^ns^	0.92 ± 0.40^***^	0.58 ± 0.28^***^
**17**	6.83 ± 1.78*	4.08 ± 1.13^*^	2.58 ± 0.73^**^
**18**	2.17 ± 0.65^**^	6.33 ± 1.49^ns^	7.00 ± 1.71^ns^
**19**	4.58 ± 1.43^ns^	4.17 ± 1.02^*^	3.67 ± 1.28^**^
**14**	3.25 ± 0.89^*^	3.33 ± 0.88^**^	1.83 ± 0.79^***^
**20**	2.08 ± 0.92^**^	2.75 ± 0.82^**^	7.08 ± 2.10^ns^
**21**	1.50 ± 0.68^***^	5.08 ± 1.83^ns^	3.83 ± 1.54^**^
**22**	2.00 ± 0.81^**^	2.75 ± 1.01^**^	5.83 ± 1.63^*^

^a^Values are the mean of leaf–sheath bending angle, normalized to the average of negative control (12 ± 2.4). Data are expressed as mean ± standard deviation of two independent experiments, each with at least six replicates (*n* = 12). Statistical significance was determined using the Student’s t-test comparing each treatment to the positive control (**1**). ns = not significant; *p < 0.05, **p < 0.01, ***p < 0.001. Brassinolide (**1**) was used as positive control.

The data show that the plant response to exogenous application of brassinolide (**1**) (positive control) or synthetic BR analogs strongly depends on the chemical structure of the BRs. For example, the activity increases with increasing concentration of **1**, and analogs **18**, **20** and **22**, whereas the opposite effect is obtained by applying analogs **12** and **17**. On the other hand, the bioactivity of synthetic analogs **19**, **14** and **21** does not vary with increasing concentration. It is well established that lamina inclination is promoted by brassinolide and that other phytohormones participate in the regulation of this process as well [35,39]. Thus, the concentration dependence of lamina inclination activity could be attributed to multiple factors such as inhibition or activation of factors affecting BRs activity [40]. This effect becomes more important at highest concentrations and therefore, the relationship between activity and chemical structures should be made at the lowest tested concentration [28,41].

Comparison of activities at 1 × 10^−8^ M show that the activity of analogs with benzoate function at C-22 change with the nature of the substituent in the *para* position. The unsubstituted derivative **12** and compound **19**, substituted with a Cl atom, are as active as the positive control **1**, whereas compound **17**, with a *p*-methyl group, is more active than **1**. On the other hand, analogs **18**, **20**, **21** and **22**, which are substituted with OCH_3_, Br, I and CN groups, are half as active as **12**. These results indicate that small substituents with no electronegative effects on the aromatic ring enhance the analogs’ bioactivity in the RLIT.

A comparison of results obtained for compounds with the same substituent at C-22, but having just one hydroxy group at C-3 (**3**) (TE analogs) [28], or only a carbonyl group in this position (**3a**) (3-DT analogs) [31] is given in Table 2.

**Table 2:** RLIT relative activity at 1 × 10^−8^ M, using brassinolide (**1**) as reference compound, for different series of BRs analogs with the same substituent at C-22 and structural differences in ring A, i.e., castasterone (CAT), teasterone (TE) and 3-dehydroxyteasterone (3-DT).

Analogue	CAT	TE	3-DT

**12** (*p*-H)	0.98	0.95	0.72
**17** (*p*-CH_3_)	1.49	1.05	0.80
**18** (*p*-OCH_3_)	0.47	1.50	0.36
**19** (*p*-Cl)	1.00	0.55	0.45
**14** (*p*-F)	0.71	0.73	0.77
**20** (*p*-Br)	0.45	0.68	–
**21** (*p*-I)	0.33	1.73	0.77

The data indicate that derivatives with a OH group at C-3 (TE analogs) are much more active than derivatives with a carbonyl group in this position (3-DT analogs). However, the addition of another OH group at C-2 (CAT analogs) increases the activity of analogs substituted with *p*-methyl (**17**) or *p*-Cl (**19**). The opposite effect is observed for those analogs substituted with *p*-OCH_3_ (**18**) or *p*-I (**21**).

#### Inhibition of root growth in *Arabidopsis thaliana* seedlings assay

**Figure 3:**
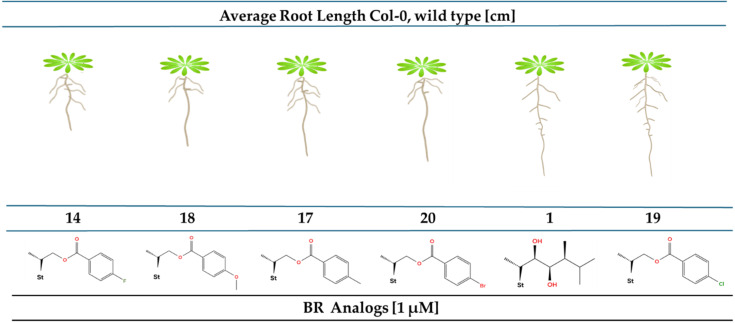
Effect of brassinolide (**1**), and BR analogs **14**, **17**–**20**) and analog **1** on root system inhibition of *A. thaliana* Col-0 wild type. Seedlings were grown on medium containing **1** and analogs (1 × 10^−6^ M) for 6 days. DMSO was used as negative control. For each treatment, more than 20 seedlings were analyzed. St = steroidal skeleton of the structure.

The root of *A. thaliana* has been used as a model to study cell division and elongation processes [42]. It has been shown that BRs affect root growth of *Oryza sativa* and *A. thaliana* in a dose-dependent manner. At low concentrations a slight root elongation is observed, whereas at higher concentrations a marked inhibition of growth is obtained [43–44]. These observations are consistent with reports showing that BRs modulate cell division and elongation processes in a concentration-depending manner [45]. The inhibitory effects on root growth of *A. thaliana* seedlings (Columbia ecotype, Col-0), caused by exogenous application of brassinolide (**1**) and 2α,3α-dihydroxylated analogs, **14**, **17**–**20** are shown in Figure 3.

The root lengths measured for *A. thaliana* grown in presence of **1**, **12**, **16a**, analogs **17**–**22** at 1 × 10^−6^ M are summarized in Table 3.

**Table 3:** Effect of brassinolide (**1**), and BR analogs with 23,24-bisnor-5α-cholane-type side chains (**12**, **14** and **17**–**22**) and analog **16a,** applied at 1 × 10^−6^ M, on root growth of *A. thaliana* Col-0 wild type^a^.

Treatments	Root length [cm]	Treatments	Root length [cm]

control	0.93 ± 0.05^†‡^	**19**	0.98 ± 0.16^†‡§^
**1**	0.93 ± 0.12^†‡§^	**14**	0.49 ± 0.11^*^
**16a**	1.13 ± 0.22^‡§^	**20**	0.87 ± 0.08^†^
**12**	1.07 ± 0.23^†‡§^	**21**	1.16 ± 0.05^§^
**17**	0.86 ± 0.14^†^	**22**	1.16 ± 0.18^§^
**18**	0.67 ± 0.24^*†^		

^a^Five days old *A. thaliana* seedlings (Columbia ecotype, Col-0) treated with 1 × 10^−6^ M of **1** (positive control) and BR analogs, whereas 0.1% of DMSO was used as negative control. For each treatment, more than 20 seedlings were analyzed. Means followed by the same symbol (^†^, ^‡^, ^§^, and *) do not differ significantly according to Duncan’s multiple range test (*p* ≤ 0.05)”.

The activities displayed by the unsubstituted analog **12**, and that with a Cl atom at the *para*-position, **19**, are comparable to that shown by brassinolide (**1**). Interestingly, those analogs containing F and OCH₃ at the *para*-position, **14** and **18**, respectively, exhibit activities that are higher than that measured for **1** at the tested concentration (1 × 10^−6^ M). On the other hand, the compound with no benzoyl function in the side chain, **16a**, and analogs with I and CN substituents, **21** and **22**, respectively, show the lowest activities at 1 × 10^−6^ M. These results are quite similar to those previously reported for compounds **4**–**11**, in which ring A has one hydroxy group at C-3 [28]. However, analogs with I and CN substituents in that series, **10** and **11**, are much more active than analogs **21** and **22**.

In summary, for this type of dihydroxylated analogs the biological root inhibitory activity follows the order: **14** > **18** > **17** > **20** > **1** > **19** > **12** > **21** = **22**. The highest activity observed for both mono- and dihydroxylated analogs is compound **14** having a small and very electronegative substituent, i.e., F atom at the *para*-position.

#### Western blotting analyses of compounds 2α,3α-dihydroxy-5α-cholan-6-oxo-23,24-dinor-22-(4-substituted)-benzoate-type treatment on BES1 protein

Western blot is a very useful, sensitive, specific and efficient technique for the detection and identification of specific proteins from a complex mixture of proteins. In addition, it is possible to quantify their expression by using suitable primary and secondary antibodies to visualize it.

It has been established that the dephosphorylated forms of BZR1 and BES1, and their concomitant accumulation in the nucleus, control the activity in the BRs signaling pathway [32,46], whereas their phosphorylated forms remain inactive and unstable and can be degraded by the proteasome under normal conditions [46]. On the other hand, accumulation of non-phosphorylated BES1 protein is specific to BRs, as other plant hormones such as auxin, cytokinin, abscisic acid and gibberellin do not cause BES1 accumulation [47], therefore this assay is accurate and complements the biological tests performed above.

Thus, in this study, the Western blot analysis was used to detect BES1 in presence of different exogenously applied BR analogs (**12**, **14** and **17**–**22**). The main idea is to correlate the amount and form of BES1 with the activity exhibited by these analogs on the root growth assay.

The results of the immunoblot analysis of proteins with an antibody specific for BES1, in roots of *A. thaliana* ecotype Col-0 plants are shown in Figure S67, Supporting Information File 1. The results are expressed as percentage of dephosphorylated BES1 (dBES1) for a negative control, brassinolide (used as positive control), and exogenously applied BR analogs. In Figure S68, Supporting Information File 1 values are represented of experimental percentages relative to both the negative control and positive control. From comparison of data with the negative control relative values of 1.33, 2.56 and 3.70, have been obtained for treatment with **1**, **12** and **19**, respectively, at 1 × 10^−6^ M . So, by considering the negative control it becomes clear that these compounds induce significantly high dBES1 overexpression. The following discussion has been made using the values relative to **1**, i.e., the expression values shown above the bold columns are relative to brassinolide, the positive control. The data show that the highest relative accumulation in dBES1 overexpression, 1.93 and 2.79, are observed for analogs **12** and **19**, respectively. The relative values follow the order **19** > **12** > **1** > **21** > **22** > **14** > **18** > **20** > **17**. These values suggest that these compounds should follow a similar trend in activities related to the BRs signaling process. However, this assumption is not validated by the results obtained in the root inhibition assay which gave a different order of activities (Figure 3, Table 2). In this assay the most active analogs are **14** and **18**. However, even though RLIT was performed at much lower concentrations and in rice instead of *Arabidopsis*, the dBES1 overexpression data and the RLIT results obtained at 1 × 10^−8^ M follow a similar pattern. This result is probably a mere coincidence, but we believe it is worth to mention it.

### Molecular docking study

Molecular docking is a computational tool that is widely used to evaluate the association process of BR analogs to the active site of *A. thaliana*. The results provide information on the most probable configuration, the main interactions, and the binding energy as a function of the chemical structure of BR analogs. The lower binding energy is commonly associated to the most active BR analog. Thus, docking results have allowed to explain experimental correlations between activity and chemical structure of BR analogs [34,48–49]. For example, previous molecular docking studies have predicted similar or better binding energies than brassinolide (**1**) for compounds with a phenyl ring with small groups such as fluorine, chlorine, or methyl [34]. In this work, molecular docking simulations were performed for all dihydroxylated analogs using the crystal structure of the BRI1–BAK1 complex (PDB ID: 4M7E) (Figure S69 and Table S2, Supporting Information File 1). This heterodimeric complex is commonly used because it represents the most structurally and functionally comprehensive model system resolved by X-ray crystallography. Unlike BRL1 and BRL3, for which only monomeric ectodomain structures are available, the 4M7E structure enables modeling of the full receptor–co-receptor assembly required for the “molecular glue” mechanism involving BAK1 [50–51]. Furthermore, BRL2 was also excluded from the analysis because it does not exhibit high-affinity binding to brassinolide and is therefore considered non-functional in ligand perception within this signaling context. The calculated binding energy values are presented in Table 4.

**Table 4:** Average binding energies calculated over at least 10 poses, for brassinolide (**1**) and synthetic analogs (**12**, **14**, **16a** and **17**–**22**).

Compounds	Δ*E*_b_, kcal/mol	Compounds	Δ*E*_b_, kcal/mol

**1**	−13.0	**19**	−14.0
**16a**	−11.6	**14**	−13.1
**12**	−13.2	**20**	−13.1
**17**	−12.9	**21**	−12.9
**18**	−12.9	**22**	−13.0

**Figure 4:**
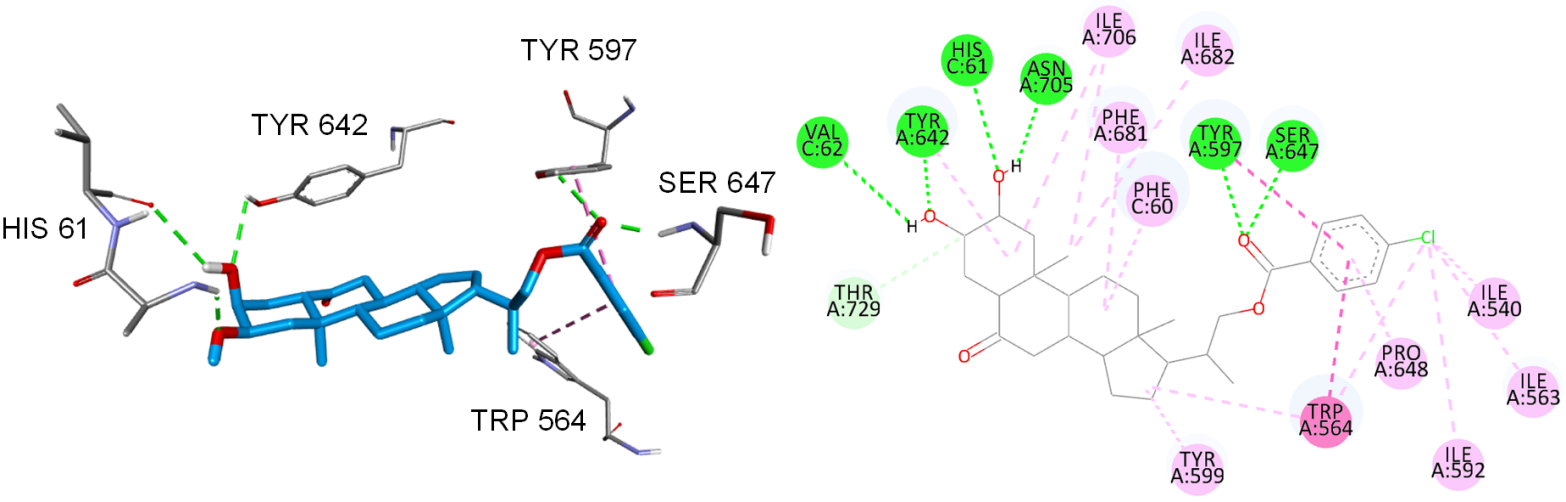
Binding modes of compound **19** into BRI1–BAK1 heterodimer. Hydrogen bonds are represented in green segmented lines. π–π stacking is represented by dark pink segmented lines. Hydrophobic interactions are represented by pink segmented lines.

Interestingly, the estimated binding energy of compound **19**, −14.0 kcal/mol, is lower than that obtained for brassinolide (**1**) (−13.0 kcal/mol). As the predicted conformations are similar, this difference is attributed to six H-bond interactions: two of them involve the ArCO carbonyl group, which interacts with Ser647 (1.89 Å) and Tyr597 (2.4 Å), other two are established between the hydroxy group at C-2 and residues Tyr642 (2.7 Å) and Val62 (2.8 Å), and finally two extra H-bonds are formed between the hydroxy group at C-3 and residues His61 (2.1 Å) and Asn705 (2.4 Å). Additionally, the aromatic ring of compound **19** generates two π–π stacking interactions with Trp564 and Tyr597. Finally, there are also van der Waals interactions with residues Phe60, Phe681, Ile682, Ile706, Tyr642, Tyr599, Pro648, Ile540, Ile563, and Trp564 (Figure 4).

The calculated binding energies suggest the following order of activity **19** > **12** > **14** = **20** > **22** = **1** > **21** > **17** > **18** > **16a**.

Considering that binding to the active site is the initial step in BRs signaling process it is interesting to verify if this calculated pattern is in line with the experimental results obtained for dephosphorylation of BES1 and root inhibitory activity.

From a comparison of binding energy and relative accumulation in dBES1 overexpression it might be expected that there is an acceptable correlation between both parameters. For example, compound **19**, possessing a chlorine substituent on the phenyl ring, has an outstanding molecular coupling in BRI1, and at the same time shows a significant increase of BES1 dephosphorylation. However, an increase in dBES1 should induce a greater root inhibition and lower cell growth at high concentrations of exogenously applied BRs. However, in the presence of compound **19** the root inhibition is as high as that observed for brassinolide, but much lower than that obtained for compounds **14** and **18**. The latter is poorly docked into the binding site and its dBES1 is equally poor. But surprisingly, its root inhibition activity is one of the highest. Similar results are found for compounds **14** and **20**.

Consequently, the data suggest that the initial step of the signaling process and the subsequent dephosphorylation of BES1 depend on the structure of BRs analogs in the same way. In other words, the interaction between BRs analogs and the binding site determines the response of the plant and one intermediate step. However, it seems that on the signaling cascade this structural effect is lost, and the outcome do not follow the initial pattern.

It is known that plant cells can synthesize most hormones and, therefore their regulation is decentralized. In addition, each hormone modulates its own synthesis, degradation, conjugation and oxidation via dynamic interaction with different phytohormones and on homeostasis mechanisms [52]. Consequently, exogenous application of a growth regulator initiates not only the process leading to a phenotypic response but also all those associated to its own regulation. Thus, only a fraction of the whole exogenous applied BRs effectively reaches the receptors and once bound, the active form can result in a positive or negative feedback on its own biosynthesis, adding an additional level of regulatory complexity.

For these reasons, establishing a direct correlation between one of the possible phenotypic responses (inhibition of root growth) and the level of activation of BR-dependent transcription factors (such as dBES1) is particularly difficult to achieve. In other words, the intensity of the signal does not necessarily reflect the amount applied nor does it translate linearly into the accumulation of the transcription factors responsible for activating or repressing the genes associated with growth and development. Other factors such as tissue type, plant development stage and environmental conditions might also influence the different steps of the signaling process.

## Conclusion

In this study, a series of new BR analogs with C22 benzoate-function, **17**–**22**, has been synthesized. The synthesis was carried out in two steps with high yields. The bioactivity of these analogs has been evaluated by two bioassays, rice lamina inclination test, and inhibition of root growth in *A. thaliana* seedlings. In the former test the best biological effects were observed for the analogs with 2α,3α-dihydroxy function (castasterone derivatives) as compared to those obtained for analogs with 3β-hydroxy (teasterone derivatives) and 3-carbonyl function. This result confirms that the best activity results in RLIT are given by analogs having the 2α,3α-dihydroxy configuration in ring A. Additionally, western blot analyses were performed to determine the amount of dBES1 produced in *A. thaliana* seedlings by each exogenously added analog. The results indicate that the measured bioactivity depends on the analogs’ structure and on the used bioassay. Interestingly, the plant response, western blot, and docking results obtained in *A. thaliana* do not follow the same structure–activity pattern. For example, analogs **14** and **18**, are more active than brassinolide (**1**) in the inhibition of root growth, whereas the highest relative accumulation in dBES1, are exhibited by analogs **12** and **19**. On the other hand, analogue **19** forms the most stable complex in the active site. From these results it can be concluded that the efficiency of BR analogs to initiate the signaling process is not a determining factor in the final plant response. Intermediate processes such as dephosphorylation of BES1 depend on the structure of BR analogs in the same way, but it becomes obvious that on following steps this relation is changed. Thus, additional research is needed to evaluate the BRs structure effect on additional subsequent steps of the signaling process.

## Supporting Information

File 1 NMR spectra of compounds, biological bioassays and protein–ligand interactions (molecular docking).

File 2 Experimental section.

## Data Availability

All data that supports the findings of this study is available in the published article and/or the supporting information of this article.
