# Supplementary material for: Synthesis and biological evaluation of new brassinosteroid analogs with C-22 benzoate function
Source: Beilstein J Org Chem. 2026 May 18;22:753–62. doi: 10.3762/bjoc.22.57 (PMC13202480; doi:10.3762/bjoc.22.57)
Supplement: File 1 — NMR spectra of compounds, biological bioassays and protein–ligand interactions (molecular docking). [file Beilstein_J_Org_Chem-22-753-s001.pdf]

BEILSTEIN JOURNAL OF ORGANIC CHEMISTRY

Supporting Information

for

Synthesis and biological evaluation of new brassinosteroid analogs with C-22 benzoate function

María Núñez, Camila Escobar, Mario Párraga, Mauricio Soto, Luis Espinoza-Catalán, Katy Díaz and Andrés F. Olea

Beilstein J. Org. Chem. **2026,** *22,* 753–762. doi:10.3762/bjoc.22.57

NMR spectra of compounds, biological bioassays and protein–ligand interactions (molecular docking)

License and Terms: This is a supporting information file under the terms of the Creative Commons Attribution License (https://creativecommons.org/licenses/by/4.0). Please note that the reuse, redistribution and reproduction in particular requires that the author(s) and source are credited and that individual graphics may be subject to special legal provisions.
The license is subject to the *Beilstein Journal of Organic Chemistry* terms and conditions: (https://www.beilstein-journals.org/bjoc/terms)

Table of contents

		pag.
**Figure S1**.	^{1}H NMR spectrum of 5α-cholan-6-oxo-2-ene-23,24-dinor-22-yl 4-methylbenzoate (**23**).	S8
**Figure S2**.	^{13}C NMR spectrum of 5α-cholan-6-oxo-2-ene-23,24-dinor-22-yl 4-methylbenzoate (**23**).	S8
**Figure S3**.	^{13}C-DEPT 135 NMR spectrum of 5α-cholan-6-oxo-2-ene-23,24-dinor-22-yl 4-methylbenzoate (**23**).	S9
**Figure S4**.	^{1}H-^{13}C HSQC-ed. spectrum 5α-cholan-6-oxo-2-ene-23,24-dinor-22-yl 4-methylbenzoate (**23**).	S9
**Figure S5**.	^{1}H-^{13}C HMBC spectrum of 5α-cholan-6-oxo-2-ene-23,24-dinor-22-yl 4-methylbenzoate (**23**).	S10
**Figure S6**.	^{1}H NMR spectrum of 5α-cholan-6-oxo-2-ene-23,24-dinor-22-yl 4-methoxybenzoate (**24**).	S10
**Figure S7**.	^{13}C NMR spectrum of 5α-cholan-6-oxo-2-ene-23,24-dinor-22-yl 4-methoxybenzoate (**24**).	S11
**Figure S8**.	^{13}C-DEPT 135 NMR spectrum of 5α-cholan-6-oxo-2-ene-23,24-dinor-22-yl 4-methoxybenzoate (**24**).	S11
**Figure S9**.	^{1}H-^{13}C HSQC-ed. spectrum 5α-cholan-6-oxo-2-ene-23,24-dinor-22-yl 4-methoxybenzoate (**24**).	S12
**Figure S10**.	^{1}H-^{13}C HMBC spectrum of 5α-cholan-6-oxo-2-ene-23,24-dinor-22-yl 4-methoxybenzoate (**24**).	S12
**Figure S11**.	^{1}H NMR spectrum of 5α-cholan-6-oxo-2-ene-23,24-dinor-22-yl 4-chlorobenzoate (**25**).	S13

Figure S12. ^{13}C NMR spectrum of 5α-cholan-6-oxo-2-ene-23,24-dinor-22-yl 4-chlorobenzoate (**25**). …………………………………………… S13

Figure S13. ^{13}C-DEPT 135 NMR spectrum of 5α-cholan-6-oxo-2-ene-23,24-dinor-22-yl 4-chlorobenzoate (**25**). ……………………………… S14

Figure S14. ^{1}H-^{13}C HSQC-ed. spectrum 5α-cholan-6-oxo-2-ene-23,24-dinor-22-yl 4-chlorobenzoate (**25**). ………………………………………… S14

Figure S15. ^{1}H-^{13}C HMBC spectrum of 5α-cholan-6-oxo-2-ene-23,24-dinor-22-yl 4-chlorobenzoate (**25**). ………………………………………… S15

Figure S16. ^{1}H NMR spectrum of 5α-cholan-6-oxo-2-ene-23,24-dinor-22-yl 4-bromobenzoate (**26**). …………………………………………… S15

Figure S17. ^{13}C NMR spectrum of 5α-cholan-6-oxo-2-ene-23,24-dinor-22-yl 4-bromobenzoate (**26**). …………………………………………… S16

Figure S18. ^{13}C-DEPT 135 NMR spectrum of 5α-cholan-6-oxo-2-ene-23,24-dinor-22-yl 4-bromobenzoate (**26**). ……………………………... S16

Figure S19. ^{1}H-^{13}C HSQC-ed. spectrum 5α-cholan-6-oxo-2-ene-23,24-dinor-22-yl 4-bromobenzoate (**26**). ………………………………………… S17

Figure S20. ^{1}H-^{13}C HMBC spectrum of 5α-cholan-6-oxo-2-ene-23,24-dinor-22-yl 4-bromobenzoate (**26**). ………………………………………… S17

Figure S21. ^{1}H NMR spectrum of 5α-cholan-6-oxo-2-ene-23,24-dinor-22-yl 4-iodobenzoate (**27**). …………………………………………… S18

Figure S22. ^{13}C NMR spectrum of 5α-cholan-6-oxo-2-ene-23,24-dinor-22-yl 4-iodobenzoate (**27**). …………………………………………… S18

Figure S23. ^{13}C-DEPT 135 NMR spectrum of 5α-cholan-6-oxo-2-ene-23,24-dinor-22-yl 4-iodobenzoate (**27**). ……………………………… S19

Figure S24. ^{1}H-^{13}C HSQC-ed. spectrum 5α-cholan-6-oxo-2-ene-23,24-dinor-22-yl 4-iodobenzoate (**27**). S19

Figure S25. ^{1}H-^{13}C HMBC spectrum of 5α-cholan-6-oxo-2-ene-23,24-dinor-22-yl 4-iodobenzoate (**27**). S20

Figure S26. ^{1}H NMR spectrum of 5α-cholan-6-oxo-2-ene-23,24-dinor-22-yl 4-cyanobenzoate (**28**). S20

Figure S27. ^{13}C NMR spectrum of 5α-cholan-6-oxo-2-ene-23,24-dinor-22-yl 4-cyanobenzoate (**28**). S21

Figure S28. ^{13}C-DEPT 135 NMR spectrum of 5α-cholan-6-oxo-2-ene-23,24-dinor-22-yl 4-cyanobenzoate (**28**). S21

Figure S29. ^{1}H-^{13}C HSQC-ed. spectrum 5α-cholan-6-oxo-2-ene-23,24-dinor-22-yl 4-cyanobenzoate (**28**). S22

Figure S30. ^{1}H-^{13}C HMBC spectrum of 5α-cholan-6-oxo-2-ene-23,24-dinor-22-yl 4-cyanobenzoate (**28**). S22

Figure S31. HRMS spectrum of 2α,3α-dihydroxy-5α-cholan-6-oxo-23,24-dinor-22-yl 4-methylbenzoate (**17**). S23

Figure S32. ^{1}H NMR spectrum of 2α,3α-dihydroxy-5α-cholan-6-oxo-23,24-dinor-22-yl 4-methylbenzoate (**17**). S23

Figure S33. ^{13}C NMR spectrum of 2α,3α-dihydroxy-5α-cholan-6-oxo-23,24-dinor-22-yl 4-methylbenzoate (**17**). S24

Figure S34. ^{13}C-DEPT 135 NMR spectrum of 2α,3α-dihydroxy-5α-cholan-6-oxo-23,24-dinor-22-yl 4-methylbenzoate (**17**). S24

Figure S35. ^{1}H-^{13}C HSQC-ed. spectrum 2α,3α-dihydroxy-5α-cholan-6-oxo-23,24-dinor-22-yl 4-methylbenzoate (**17**). S25

Figure S36. ^{1}H-^{13}C HMBC spectrum of 2α,3α-dihydroxy-5α-cholan-6-oxo-23,24-dinor-22-yl 4-methylbenzoate (**17**). ………………………………… S25

Figure S37. HRMS spectrum of 2α,3α-dihydroxy-5α-cholan-6-oxo-23,24-dinor-22-yl 4-methoxybenzoate (**18**). …………………………………. S26

Figure S38. ^{1}H NMR spectrum of 2α,3α-dihydroxy-5α-cholan-6-oxo-23,24-dinor-22-yl 4-methoxybenzoate (**18**). ………………………………….. S26

Figure S39. ^{13}C NMR spectrum of 2α,3α-dihydroxy-5α-cholan-6-oxo-23,24-dinor-22-yl 4-methoxybenzoate (**18**). ………………………………….. S27

Figure S40. ^{13}C-DEPT 135 NMR spectrum of 2α,3α-dihydroxy-5α-cholan-6-oxo-23,24-dinor-22-yl 4-methoxybenzoate (**18**). …………………… S27

Figure S41. ^{1}H-^{13}C HSQC-ed. spectrum 2α,3α-dihydroxy-5α-cholan-6-oxo-23,24-dinor-22-yl 4-methoxybenzoate (**18**). …………………… S28

Figure S42. ^{1}H-^{13}C HMBC spectrum of 2α,3α-dihydroxy-5α-cholan-6-oxo-23,24-dinor-22-yl 4-methoxybenzoate (**18**). …………………………… S28

Figure S43. HRMS spectrum of 2α,3α-dihydroxy-5α-cholan-6-oxo-23,24-dinor-22-yl 4-chlorobenzoate (**19**). …………………………………….. S29

Figure S44. ^{1}H NMR spectrum of 2α,3α-dihydroxy-5α-cholan-6-oxo-23,24-dinor-22-yl 4-chlorobenzoate (**19**). …………………………………….. S29

Figure S45. ^{13}C NMR spectrum of 2α,3α-dihydroxy-5α-cholan-6-oxo-23,24-dinor-22-yl 4-chlorobenzoate (**19**). …………………………………….. S30

Figure S46. ^{13}C-DEPT 135 NMR spectrum of 2α,3α-dihydroxy-5α-cholan-6-oxo-23,24-dinor-22-yl 4-chlorobenzoate (**19**). ………………………. S30

Figure S47. ^{1}H-^{13}C HSQC-ed. spectrum 2α,3α-dihydroxy-5α-cholan-6-oxo-23,24-dinor-22-yl 4-chlorobenzoate (**19**). ………………………. S31

Figure S48. ^{1}H-^{13}C HMBC spectrum of 2α,3α-dihydroxy-5α-cholan-6-oxo-23,24-dinor-22-yl 4-chlorobenzoate (**19**). ……………………………… S31

Figure S49. HRMS spectrum of 2α,3α-dihydroxy-5α-cholan-6-oxo-23,24-dinor-22-yl 4-bromobenzoate (**20**). …………………………………….. S32

Figure S50. ^{1}H NMR spectrum of 2α,3α-dihydroxy-5α-cholan-6-oxo-23,24-dinor-22-yl 4-bromobenzoate (**20**). ……………………………………. S32

Figure S51. ^{13}C NMR spectrum of 2α,3α-dihydroxy-5α-cholan-6-oxo-23,24-dinor-22-yl 4-bromobenzoate (**20**). …………………………………….. S33

Figure S52. ^{13}C-DEPT 135 NMR spectrum of 2α,3α-dihydroxy-5α-cholan-6-oxo-23,24-dinor-22-yl 4-bromobenzoate (**20**). ……………………… S33

Figure S53. ^{1}H-^{13}C HSQC-ed. spectrum 2α,3α-dihydroxy-5α-cholan-6-oxo-23,24-dinor-22-yl 4-bromobenzoate (**20**). ………………………. S34

Figure S54. ^{1}H-^{13}C HMBC spectrum of 2α,3α-dihydroxy-5α-cholan-6-oxo-23,24-dinor-22-yl 4-bromobenzoate (**20**). ……………………………… S34

Figure S55. HRMS spectrum of 2α,3α-dihydroxy-5α-cholan-6-oxo-23,24-dinor-22-yl 4-iodobenzoate (**21**). …………………………………….. S35

Figure S56. ^{1}H NMR spectrum of 2α,3α-dihydroxy-5α-cholan-6-oxo-23,24-dinor-22-yl 4-iodobenzoate (**21**). …………………………………….. S35

Figure S57. ^{13}C NMR spectrum of 2α,3α-dihydroxy-5α-cholan-6-oxo-23,24-dinor-22-yl 4-iodobenzoate (**21**). …………………………………….. S36

Figure S58. ^{13}C-DEPT 135 NMR spectrum of 2α,3α-dihydroxy-5α-cholan-6-oxo-23,24-dinor-22-yl 4-iodobenzoate (**21**). ………………………. S36

Figure S59. ^{1}H-^{13}C HSQC-ed. spectrum 2α,3α-dihydroxy-5α-cholan-6-oxo-23,24-dinor-22-yl 4-iodobenzoate (**21**). ………………………. S37

Figure S60. ^{1}H-^{13}C HMBC spectrum of 2α,3α-dihydroxy-5α-cholan-6-oxo-23,24-dinor-22-yl 4-iodobenzoate (**21**). ……………………………… S37

Figure S61. HRMS spectrum of 2α,3α-dihydroxy-5α-cholan-6-oxo-23,24-dinor-22-yl 4-cyanobenzoate (**22**). …………………………………… S38

Figure S62. ^{1}H NMR spectrum of 2α,3α-dihydroxy-5α-cholan-6-oxo-23,24-dinor-22-yl 4-cyanobenzoate (**22**). ……………………………………. S38

Figure S63. ^{13}C NMR spectrum of 2α,3α-dihydroxy-5α-cholan-6-oxo-23,24-dinor-22-yl 4-cyanobenzoate (**22**). …………………………….......... S39

Figure S64. ^{13}C-DEPT 135 NMR spectrum of 2α,3α-dihydroxy-5α-cholan-6-oxo-23,24-dinor-22-yl 4-cyanobenzoate (**22**). ………………………… S39

Figure S65. ^{1}H-^{13}C HSQC-ed. spectrum 2α,3α-dihydroxy-5α-cholan-6-oxo-23,24-dinor-22-yl 4-cyanobenzoate (**22**). ………………………… S40

Figure S66. ^{1}H-^{13}C HMBC spectrum of 2α,3α-dihydroxy-5α-cholan-6-oxo-23,24-dinor-22-yl 4-cyanobenzoate (**22**). ……………………………….. S40

Figure S67. Analysis de Immunoblot showing dephosphorylation of BES1 after treatment with active compound **12** and **19**. WB: Western Blot; dBES1: dominant Brassinosteroid-Insensitive 1 Suppressor 1; DMSO: Dimethyl sulfoxide; kDa: Kilodalton. Representative images are shown. dBES1, dephosphorylated BES1………………………… S41

Figure S68 BES1 phosphorylation status tested by immunoblot with α-BES1 antibody in roots of *Arabidopsis thaliana* ecotype Col-0 6-day-old seedlings after analogues of BR treatment. Tubulin detected with α-tubulin antibody was used as a loading control. The graph shows the percentage of dephosphorylated BES1 relative to total BES1 detected in wild-type *Arabidopsis* ecotype (Col-0) treated with brassinolide (1) after 6 days…………………………………………….

S41

Figure S69. Protein–ligand interactions with a) compound **12** b) compound **14**; c) compound **16a**, d) compound **17**, e) compound **18**, f) compound **19,** g) compound **20**, h) compound **21** and i) compound **22**. Hydrogen bonds are represented in green segmented lines. π–π stacking are represented in dark pink segmented lines. Hydrophobic interactions are represented in pink segmented lines. Visualization of the docked poses was performed using Discovery Studio Visualizer (BIOVIA, San Diego, CA, USA). ………………………… S46

Table S1. Rice lamina assays using the second leaf lamina joints (angle opening, degrees) of excised leaf segments treated with BRs analogs (**1**, **12**, **14** and **17**–**22**) at different concentrations. Brassinolide was used as positive control at the same concentrations. ………………………………………………………. S47

Table S2. Docked compounds–heterodimer protein contacts of synthetic analogs (**12**–**22**). ……………………………………………………….. S48

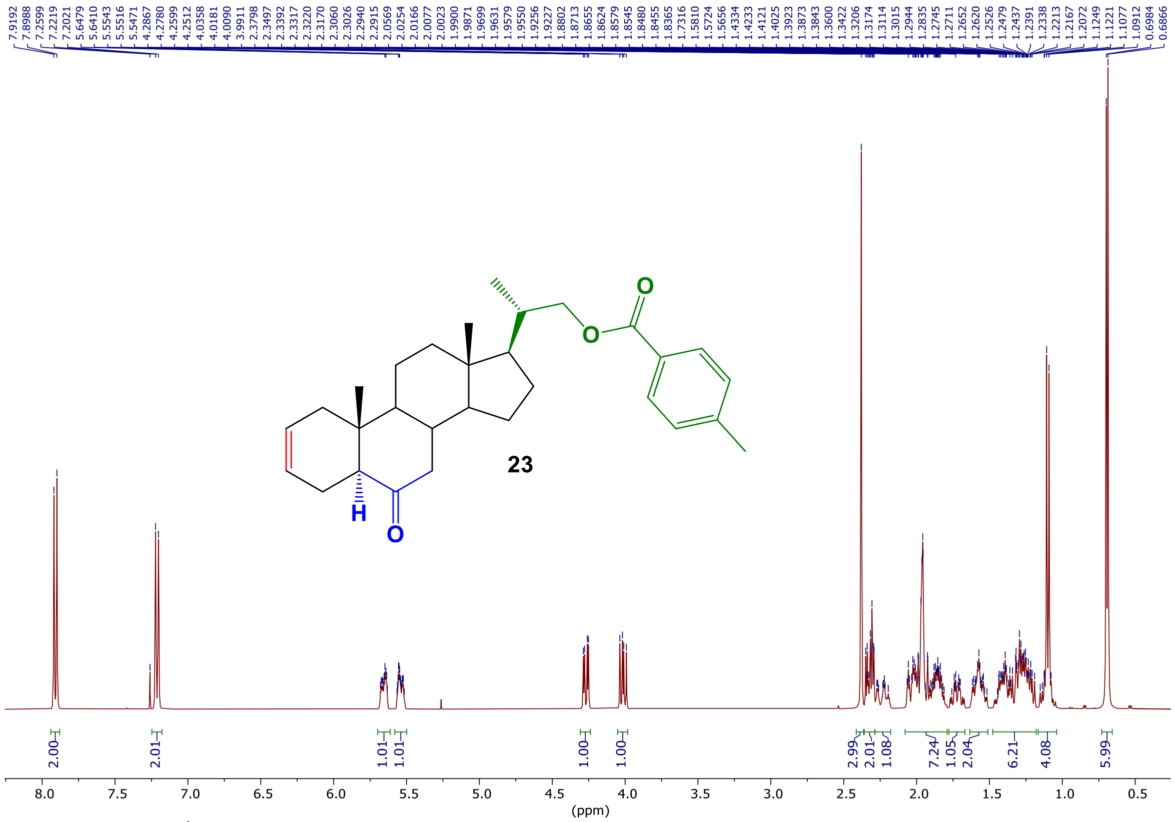

Figure S1. ^{1}H NMR spectrum of 5α-cholan-6-oxo-2-ene-23,24-dinor-22-yl 4-methylbenzoate (**23**).

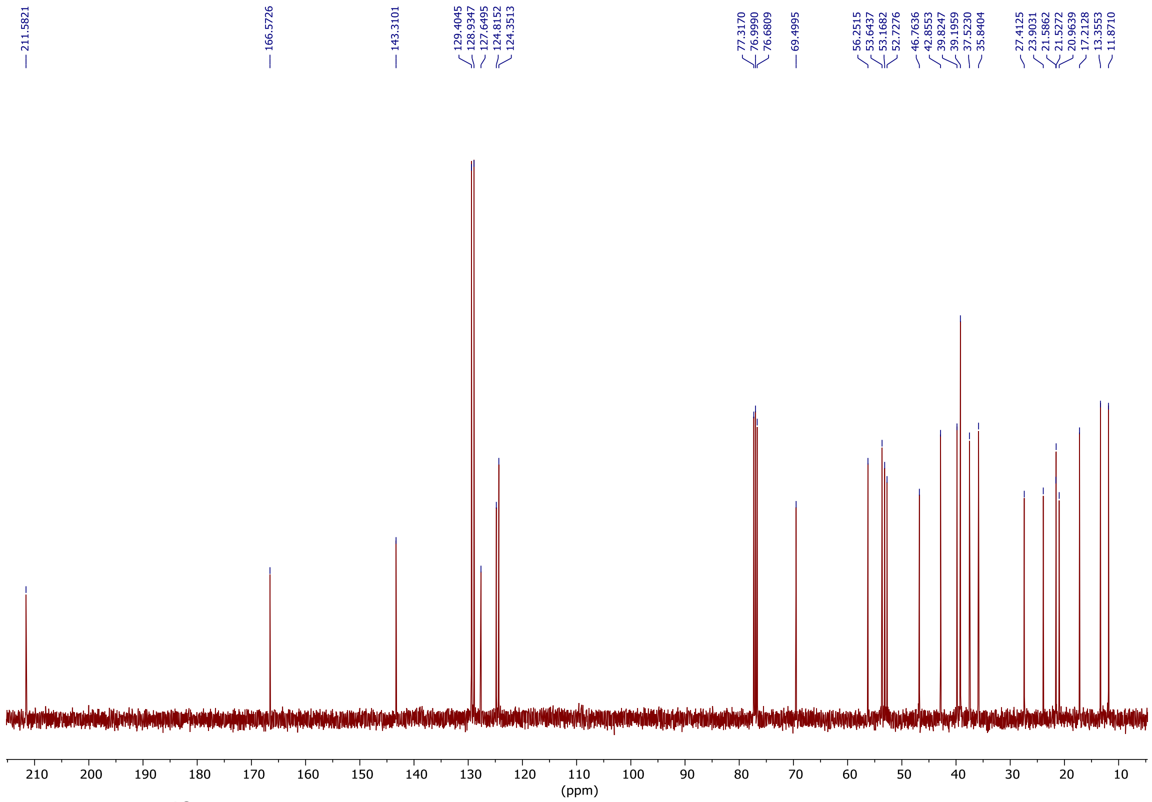

Figure S2. ^{13}C NMR spectrum of 5α-cholan-6-oxo-2-ene-23,24-dinor-22-yl 4-methylbenzoate (**23**).

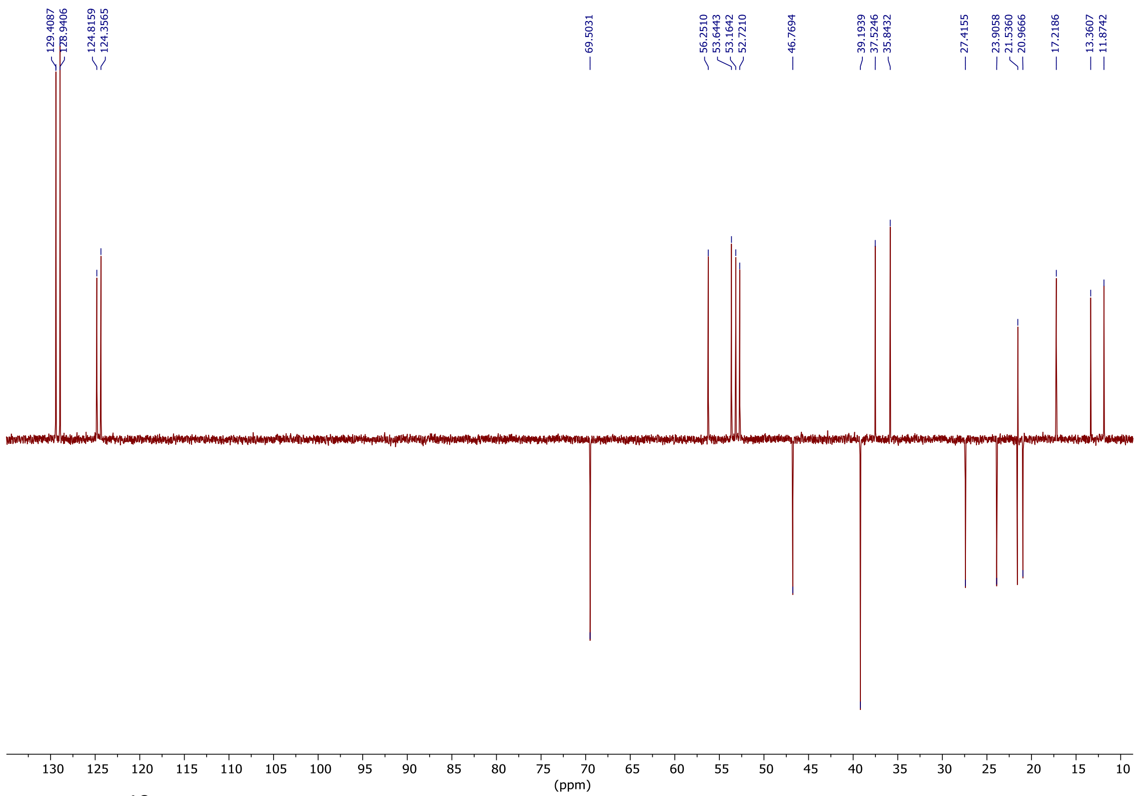

Figure S3. ^{13}C-DEPT 135 NMR spectrum of 5α-cholan-6-oxo-2-ene-23,24-dinor-22-yl 4-methylbenzoate (**23**).

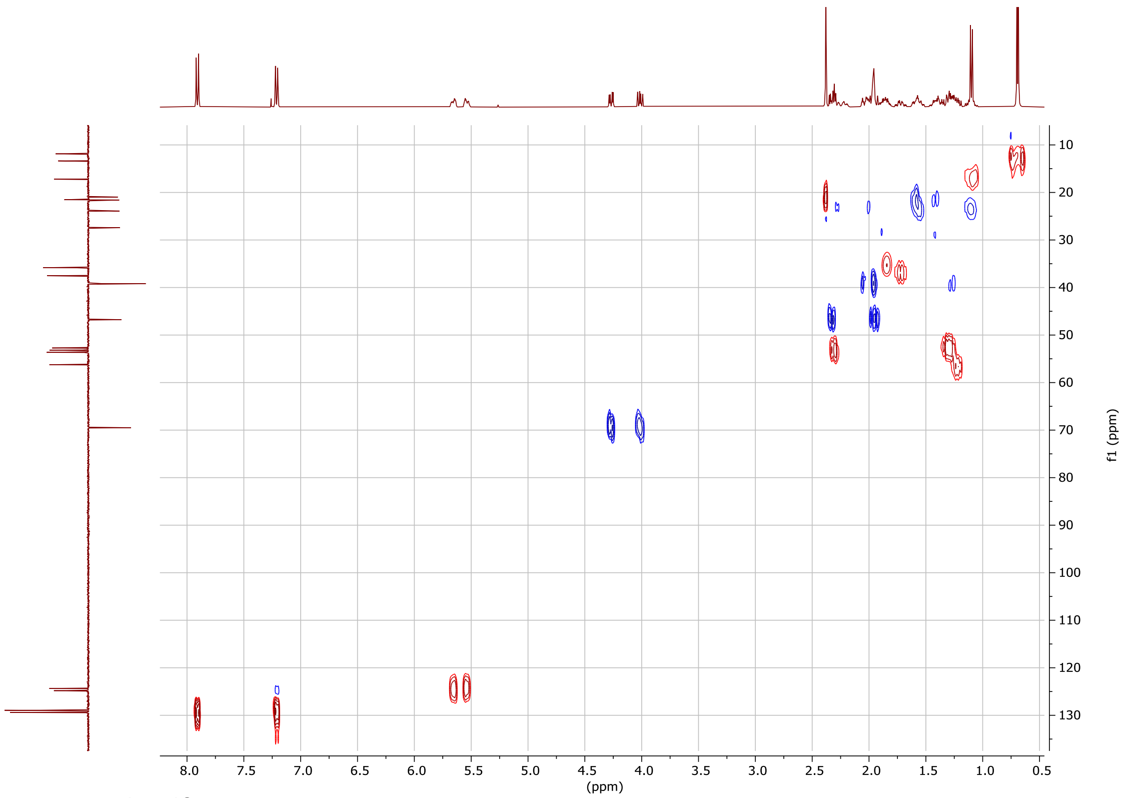

Figure S4. ^{1}H-^{13}C HSQC-ed. spectrum 5α-cholan-6-oxo-2-ene-23,24-dinor-22-yl 4-methylbenzoate (**23**).

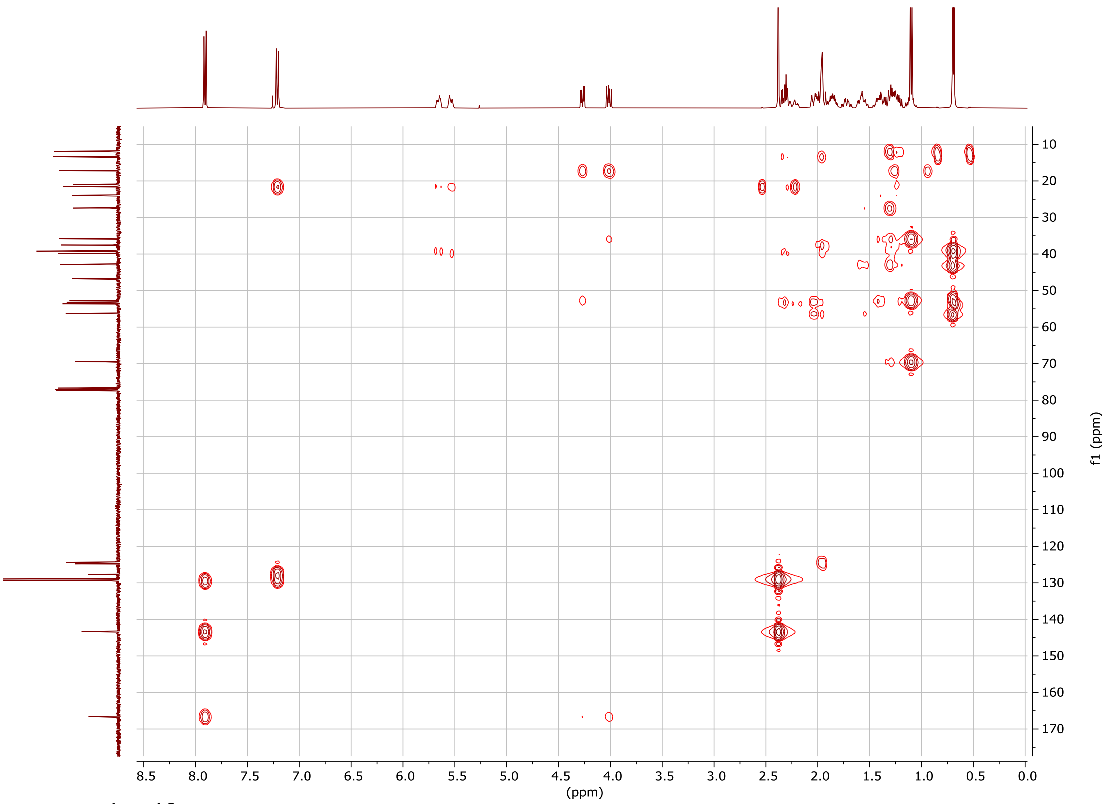

Figure S5. $^{1}H-^{13}C$ HMBC spectrum of 5α-cholan-6-oxo-2-ene-23,24-dinor-22-yl 4-methylbenzoate (**23**).

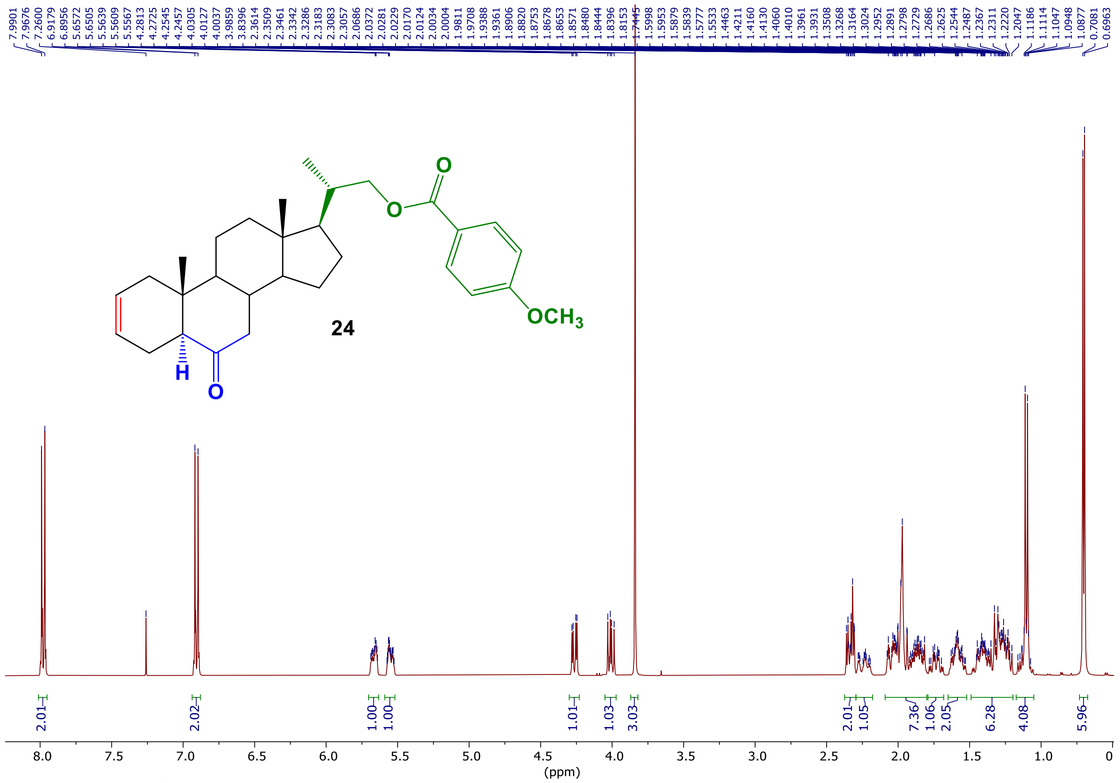

Figure S6. ^{1}H NMR spectrum of 5α-cholan-6-oxo-2-ene-23,24-dinor-22-yl 4-methoxybenzoate (**24**).

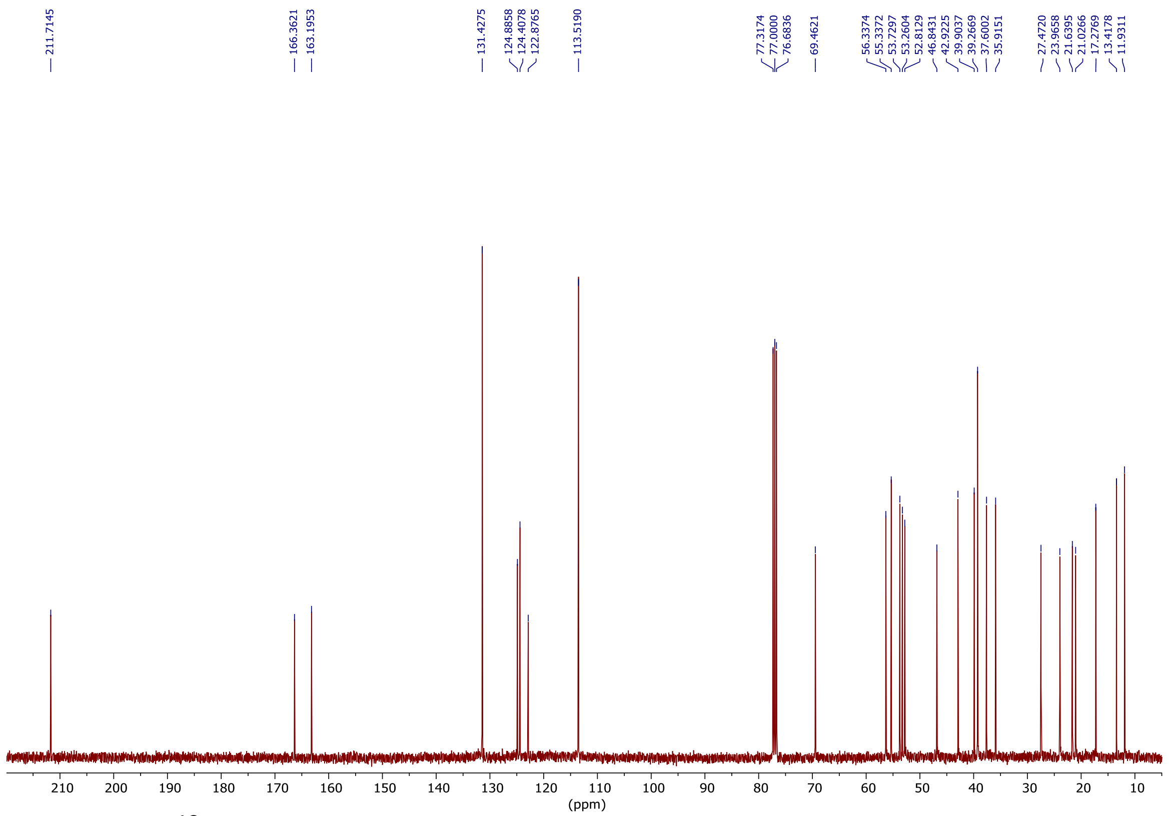

Figure S7. ^{13}C NMR spectrum of 5α-cholan-6-oxo-2-ene-23,24-dinor-22-yl 4-methoxybenzoate (**24**).

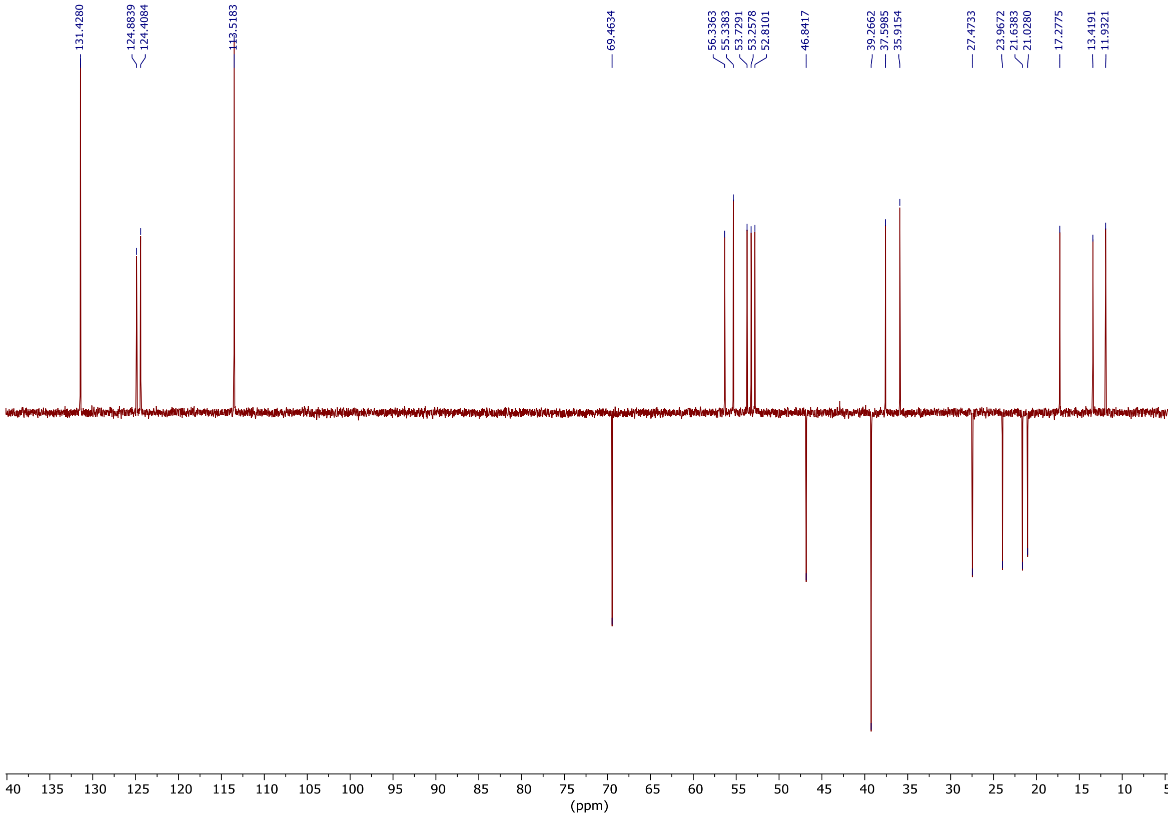

Figure S8. ^{13}C-DEPT 135 NMR spectrum of 5α-cholan-6-oxo-2-ene-23,24-dinor-22-yl 4-methoxybenzoate (**24**).

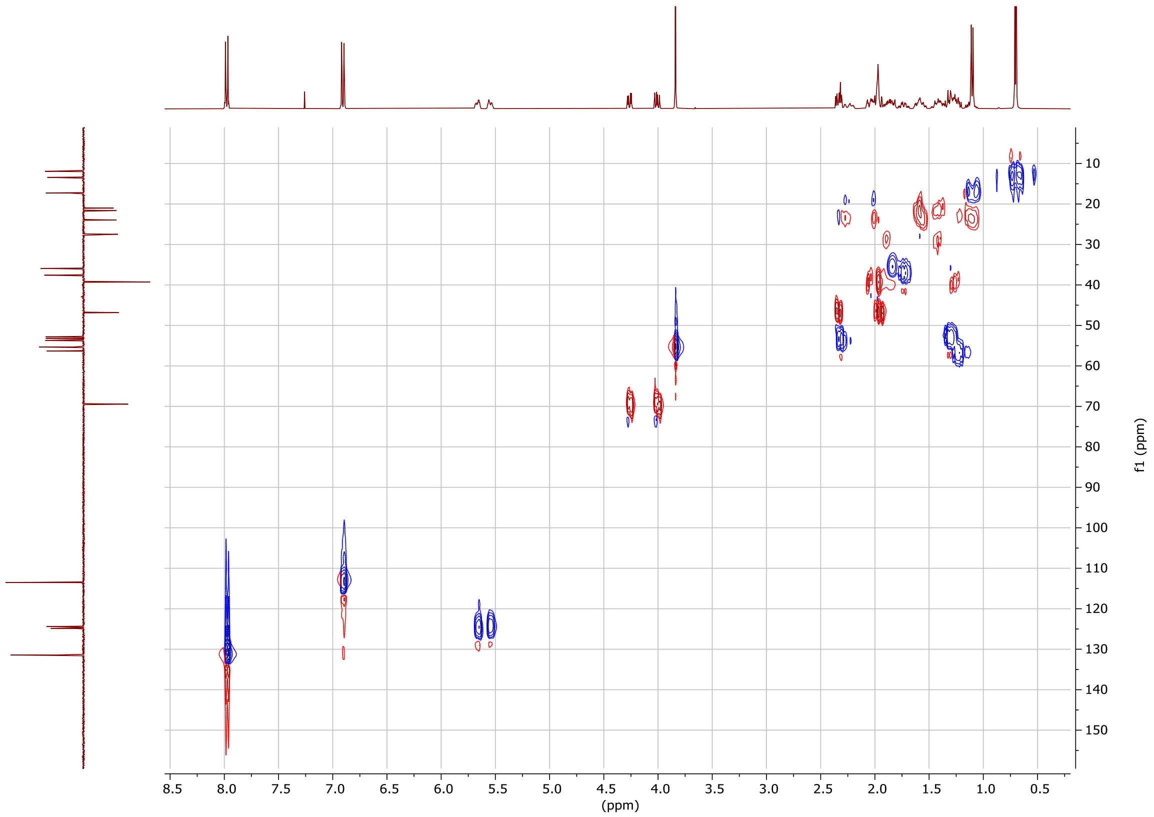

Figure S9. ^{1}H-^{13}C HSQC-ed. spectrum 5α-cholan-6-oxo-2-ene-23,24-dinor-22-yl 4-methoxybenzoate (**24**).

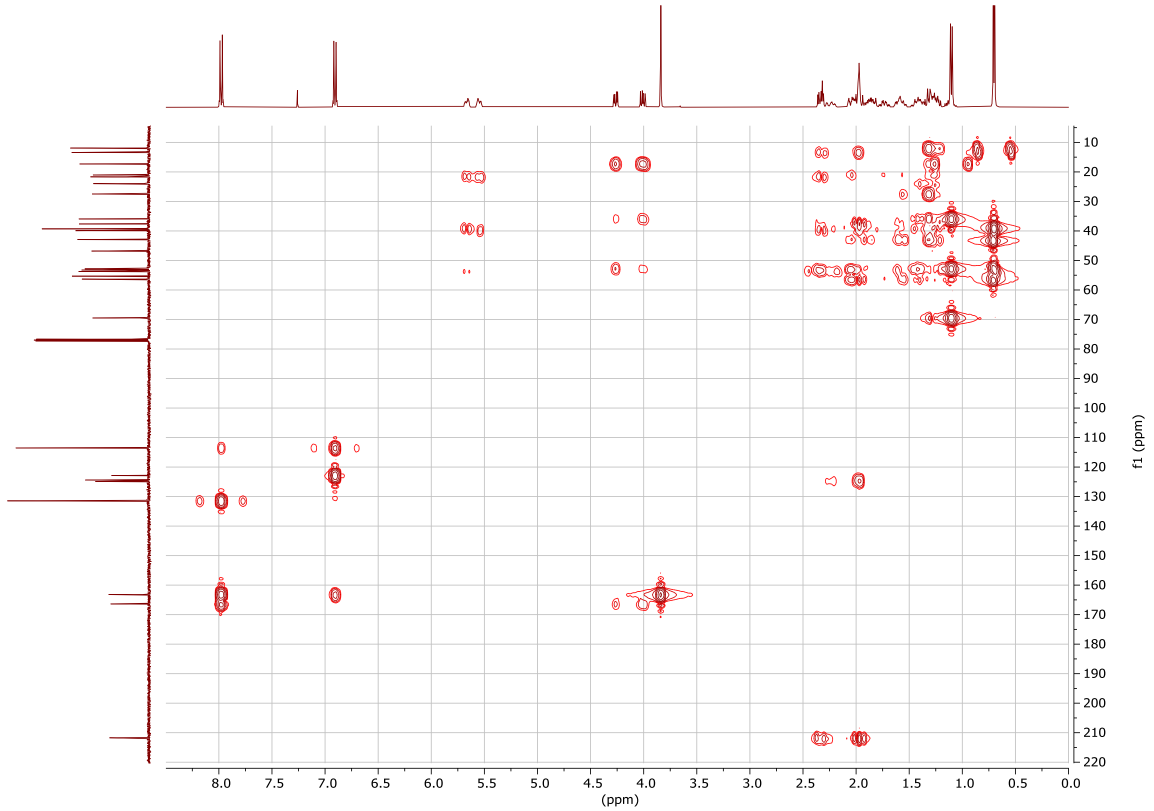

Figure S10. ^{1}H-^{13}C HMBC spectrum of 5α-cholan-6-oxo-2-ene-23,24-dinor-22-yl 4-methoxybenzoate (**24**).

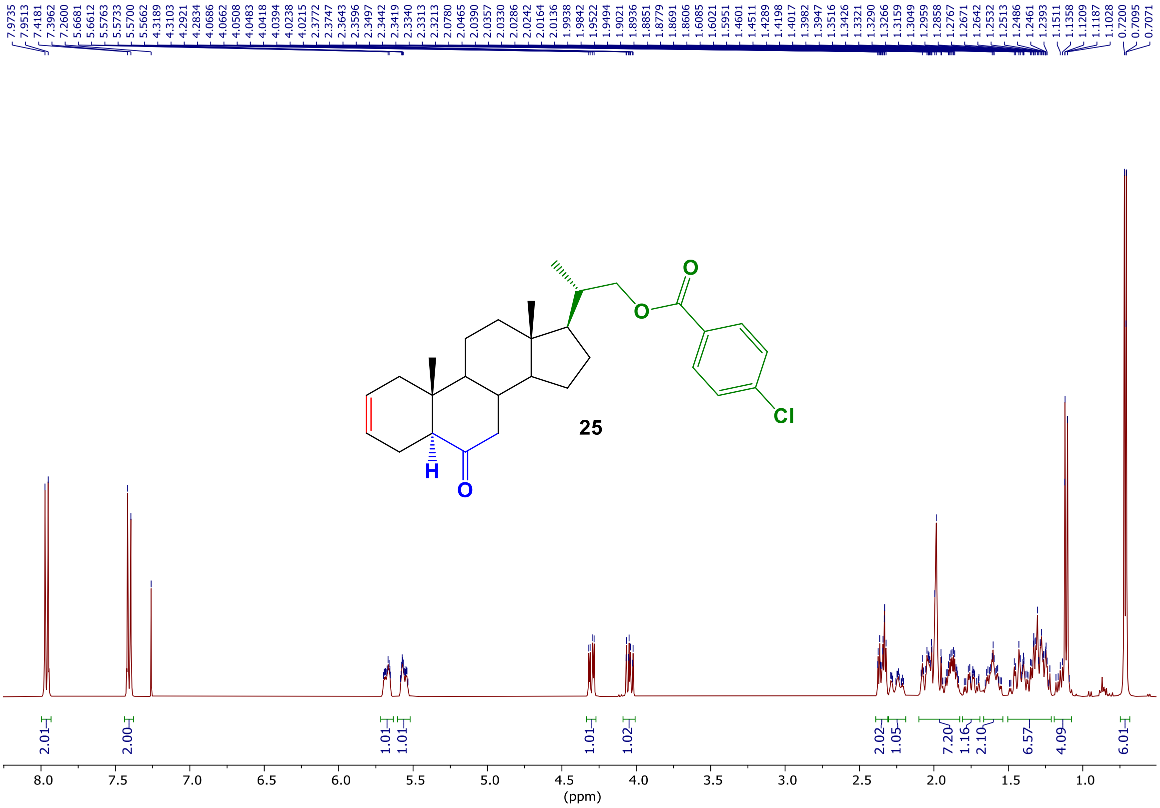

Figure S11. ^{1}H NMR spectrum of 5α-cholan-6-oxo-2-ene-23,24-dinor-22-yl 4-chlorobenzoate (**25**).

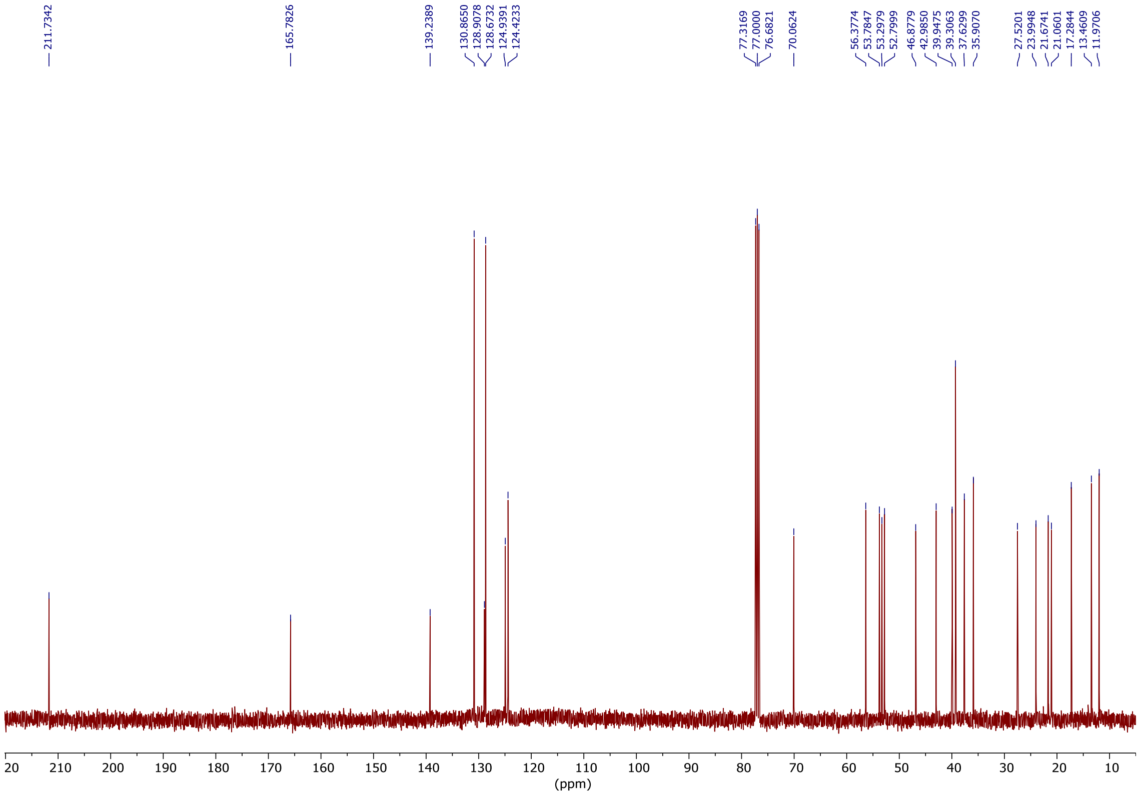

Figure S12. ^{13}C NMR spectrum of 5α-cholan-6-oxo-2-ene-23,24-dinor-22-yl 4-chlorobenzoate (**25**).

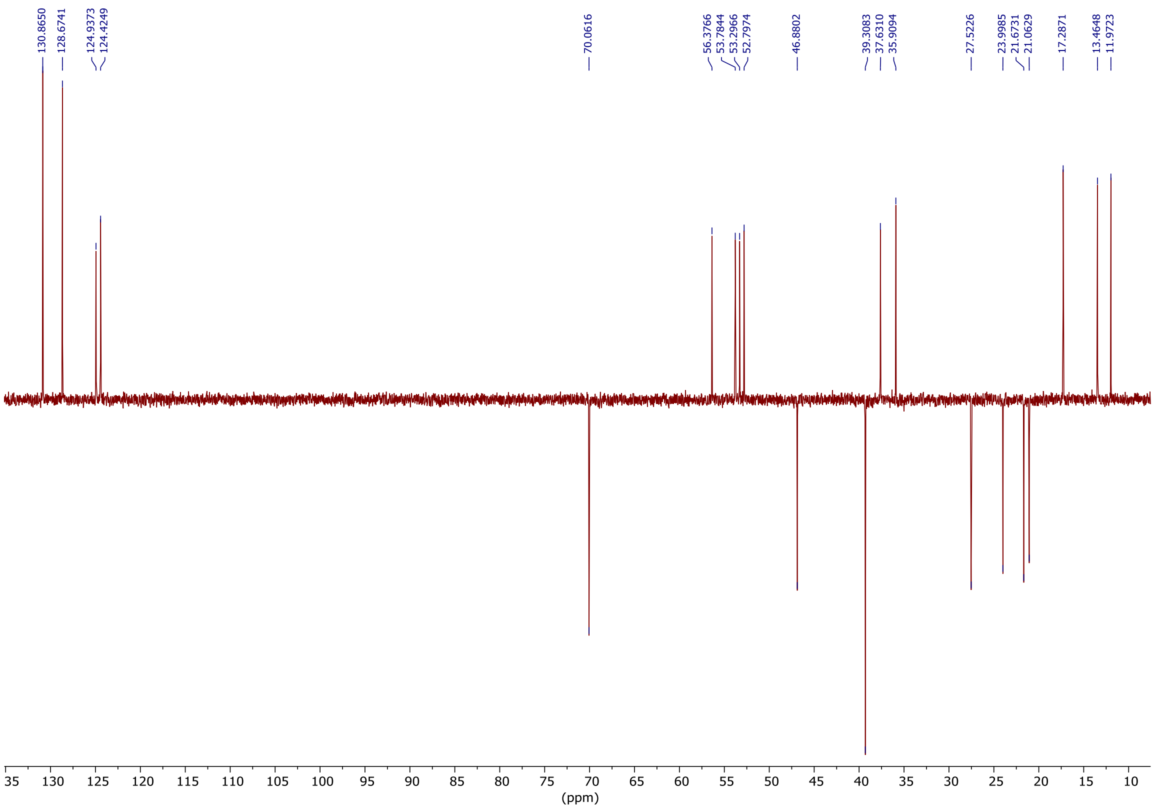

Figure S13. ^{13}C-DEPT 135 NMR spectrum of 5α-cholan-6-oxo-2-ene-23,24-dinor-22-yl 4-chlorobenzoate (**25**).

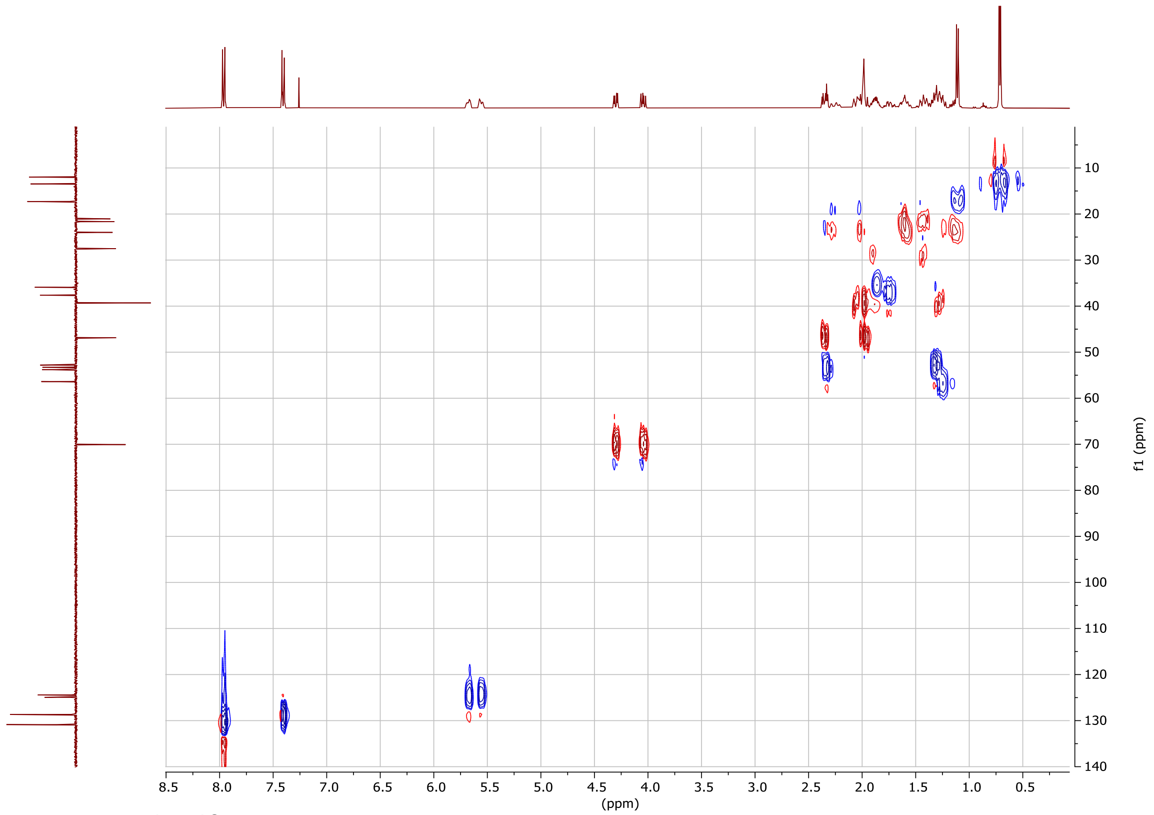

Figure S14. ^{1}H-^{13}C HSQC-ed. spectrum 5α-cholan-6-oxo-2-ene-23,24-dinor-22-yl 4-chlorobenzoate (**25**).

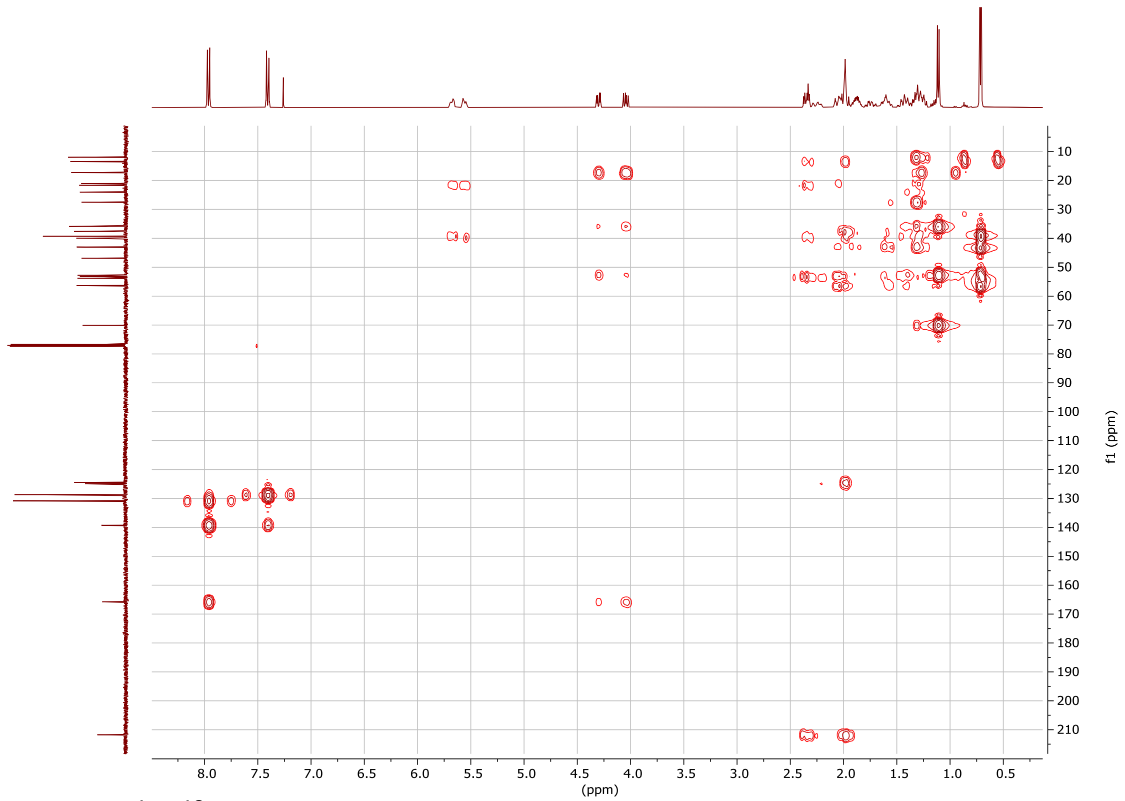

Figure S15. ^{1}H-^{13}C HMBC spectrum of 5α-cholan-6-oxo-2-ene-23,24-dinor-22-yl 4-chlorobenzoate (**25**).

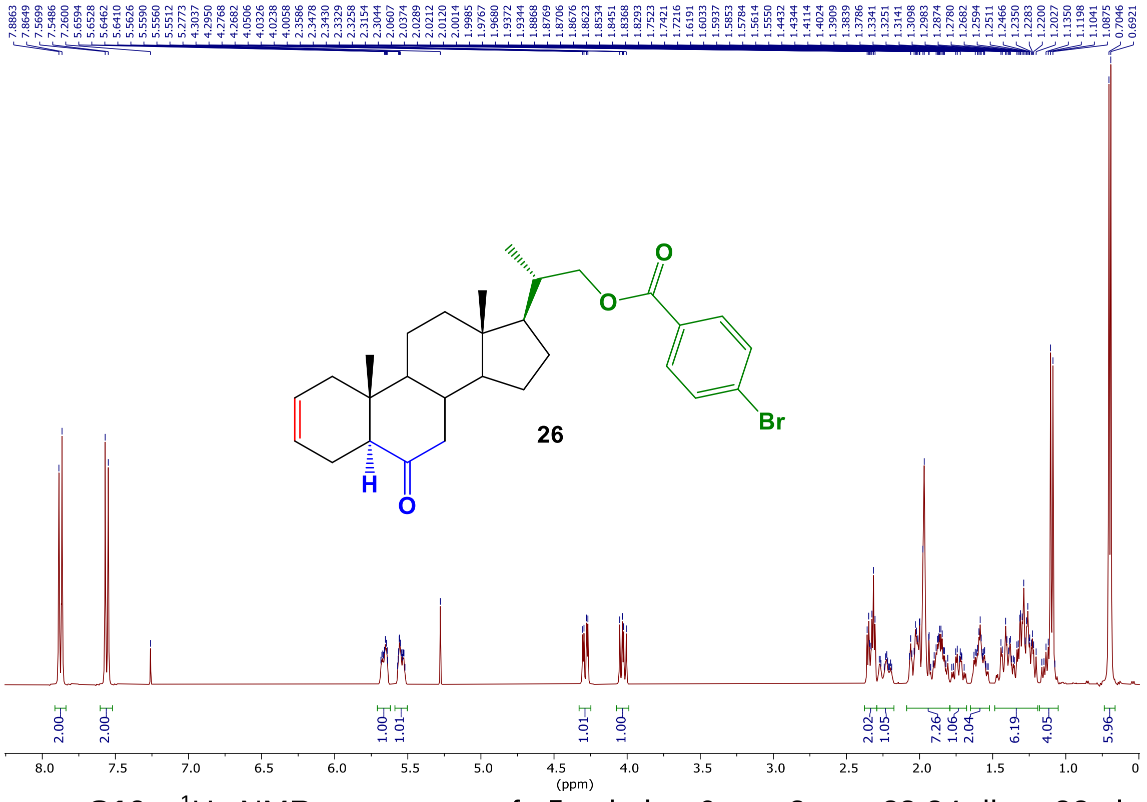

Figure S16. ^{1}H NMR spectrum of 5α-cholan-6-oxo-2-ene-23,24-dinor-22-yl 4-bromobenzoate (**26**).

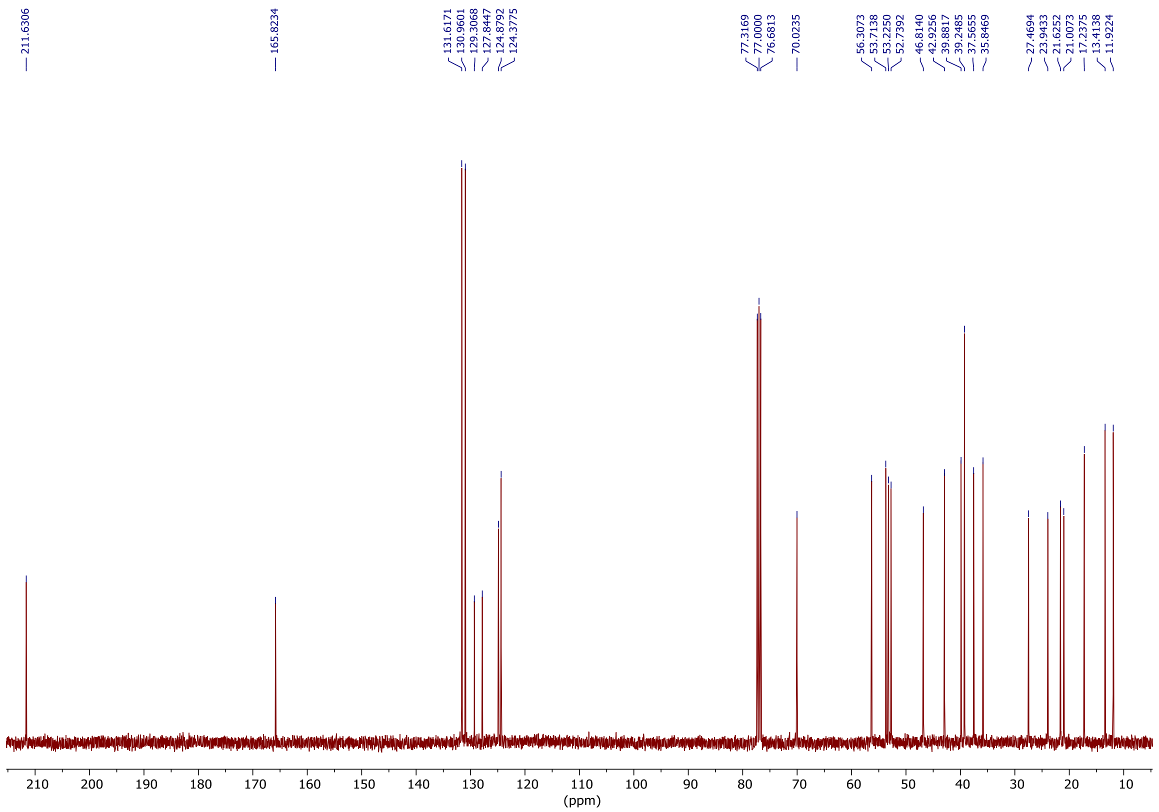

Figure S17. ^{13}C NMR spectrum of 5α-cholan-6-oxo-2-ene-23,24-dinor-22-yl 4-bromobenzoate (**26**).

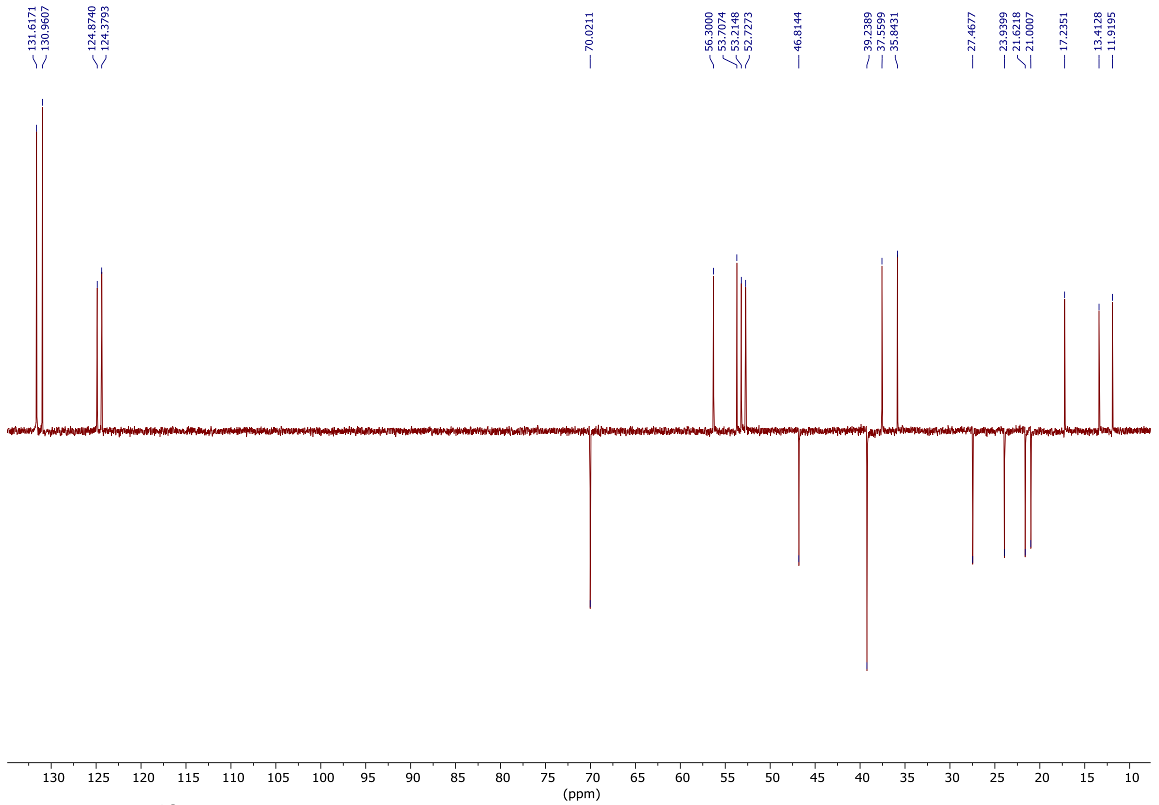

Figure S18. ^{13}C-DEPT 135 NMR spectrum of 5α-cholan-6-oxo-2-ene-23,24-dinor-22-yl 4-bromobenzoate (**26**).

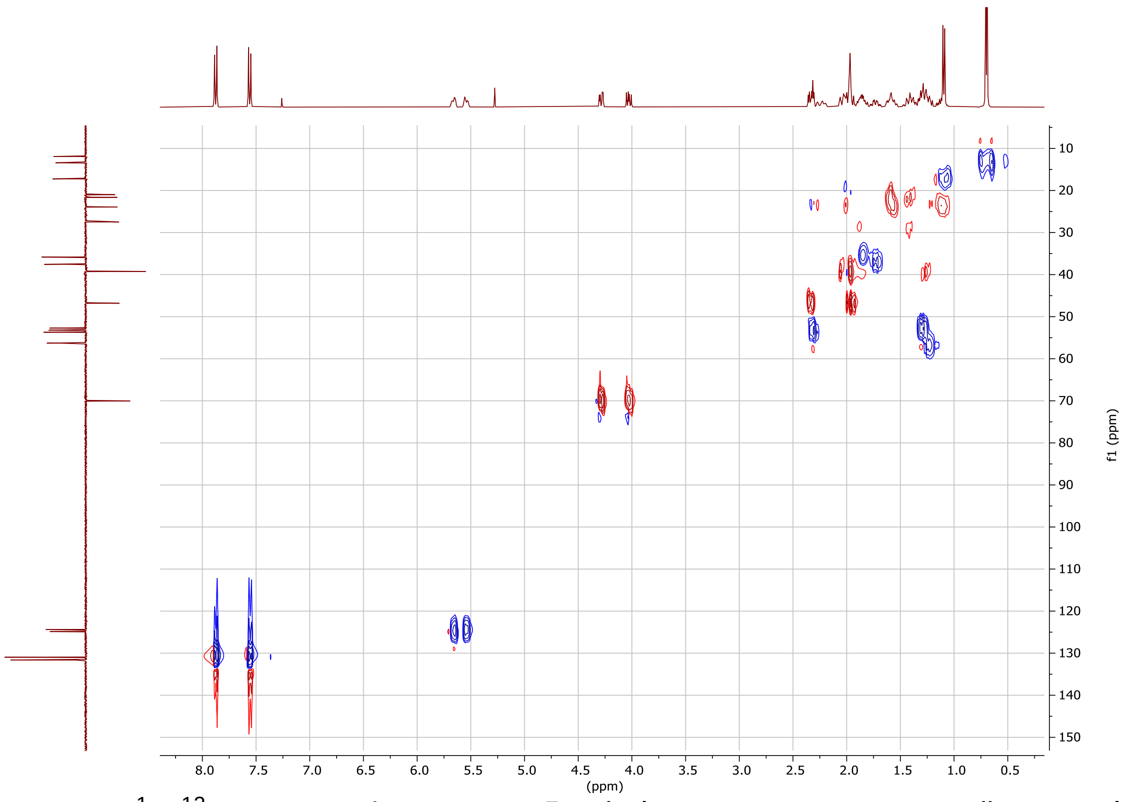

Figure S19. ^{1}H-^{13}C HSQC-ed. spectrum 5α-cholan-6-oxo-2-ene-23,24-dinor-22-yl 4-bromobenzoate (**26**).

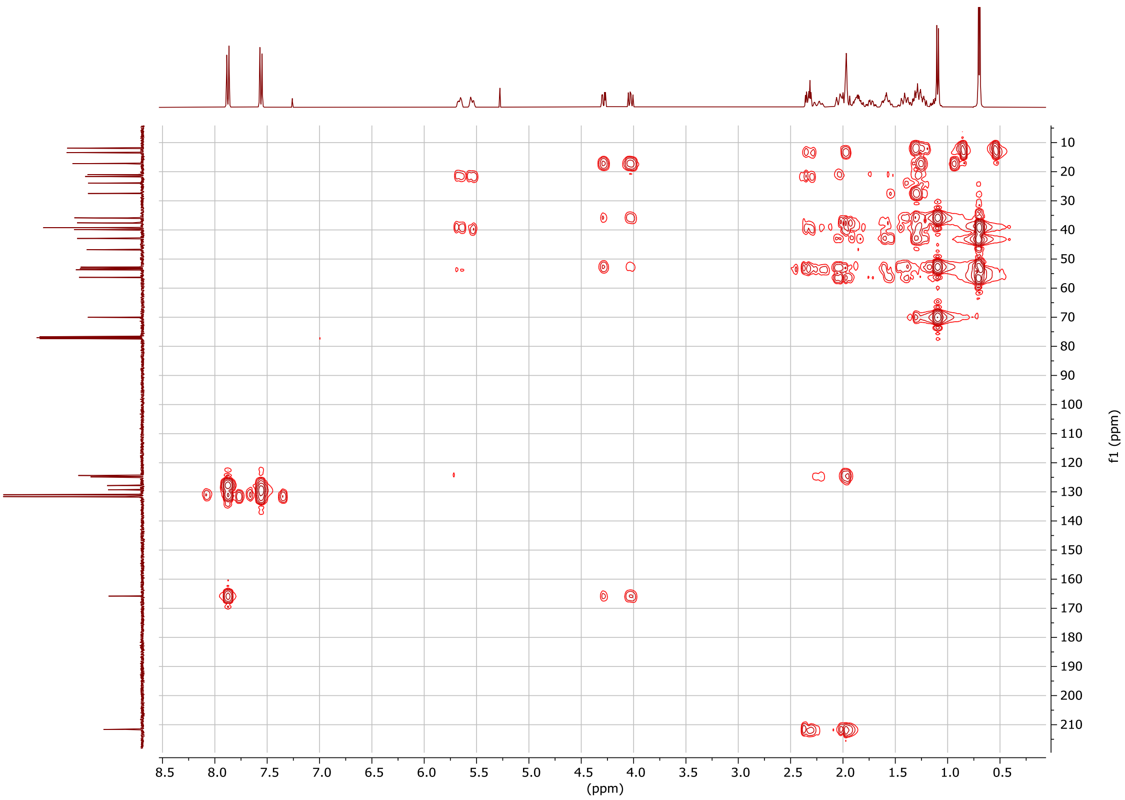

Figure S20. ^{1}H-^{13}C HMBC spectrum of 5α-cholan-6-oxo-2-ene-23,24-dinor-22-yl 4-bromobenzoate (**26**).

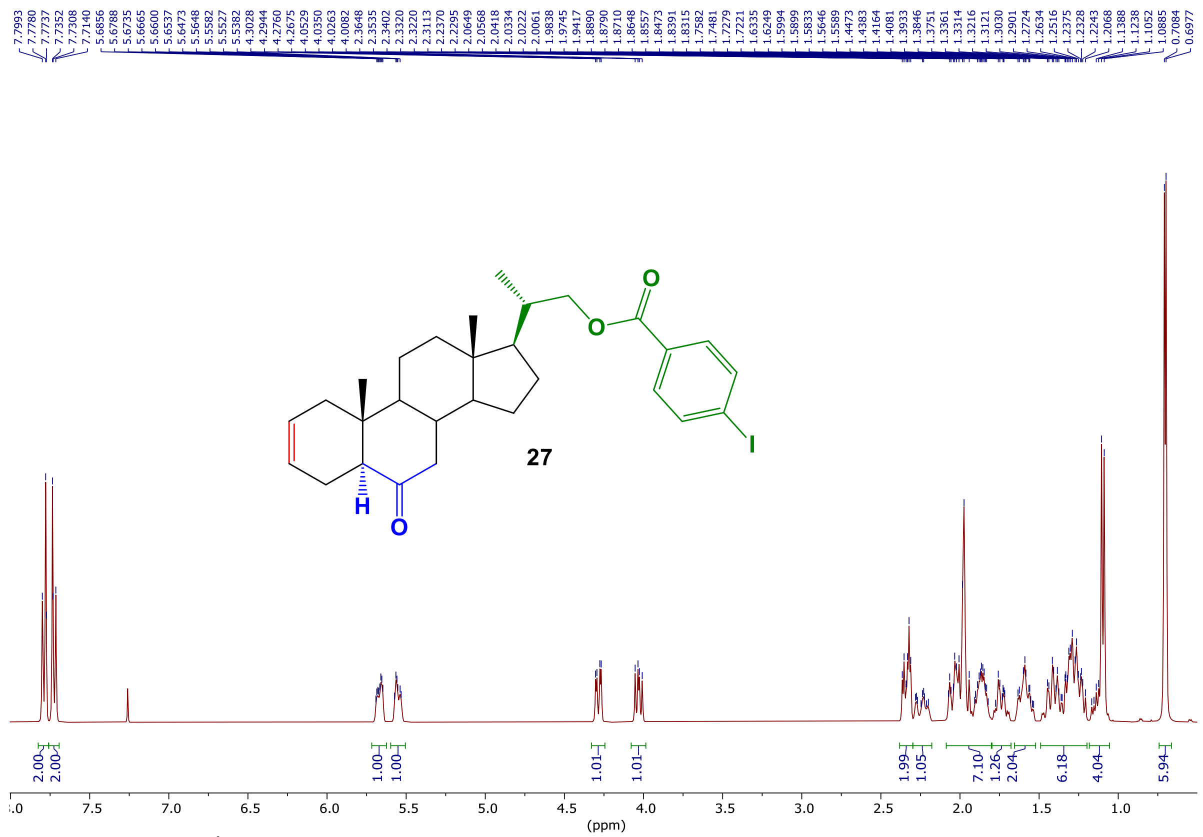

Figure S21. ^{1}H NMR spectrum of 5α-cholan-6-oxo-2-ene-23,24-dinor-22-yl 4-iodobenzoate (**27**).

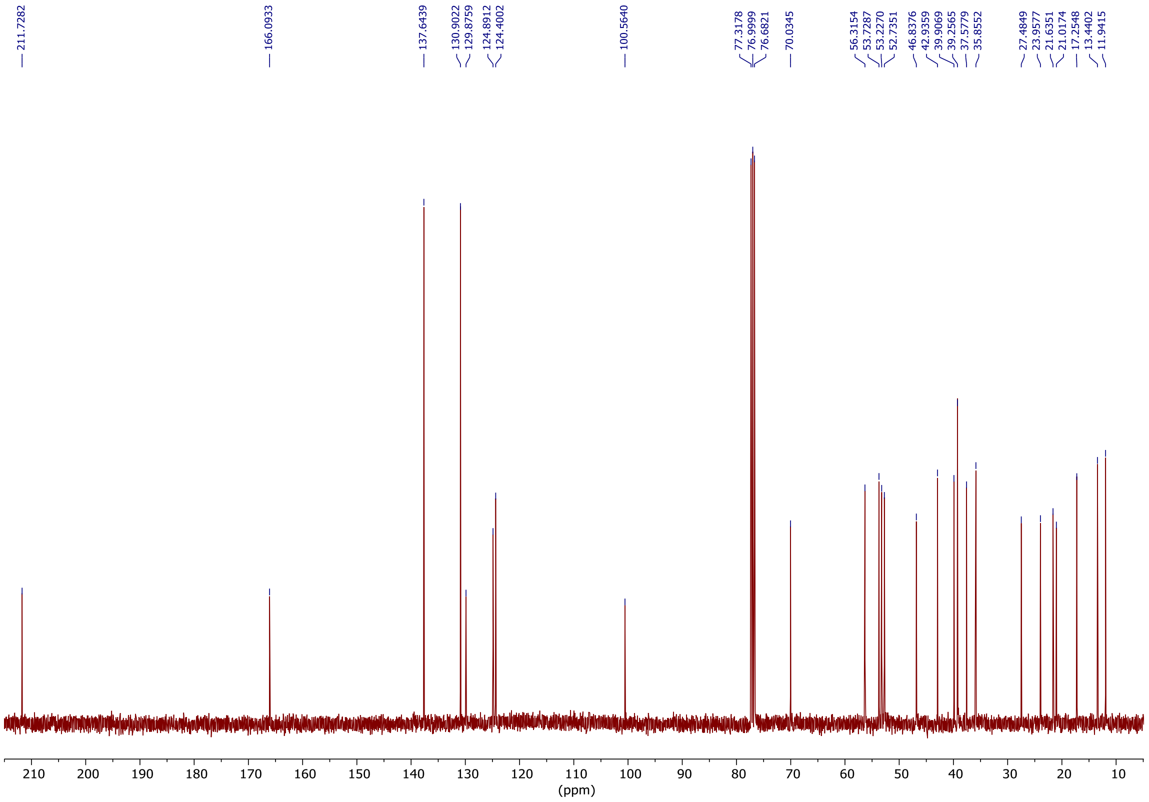

Figure S22. ^{13}C NMR spectrum of 5α-cholan-6-oxo-2-ene-23,24-dinor-22-yl 4-iodobenzoate (**27**).

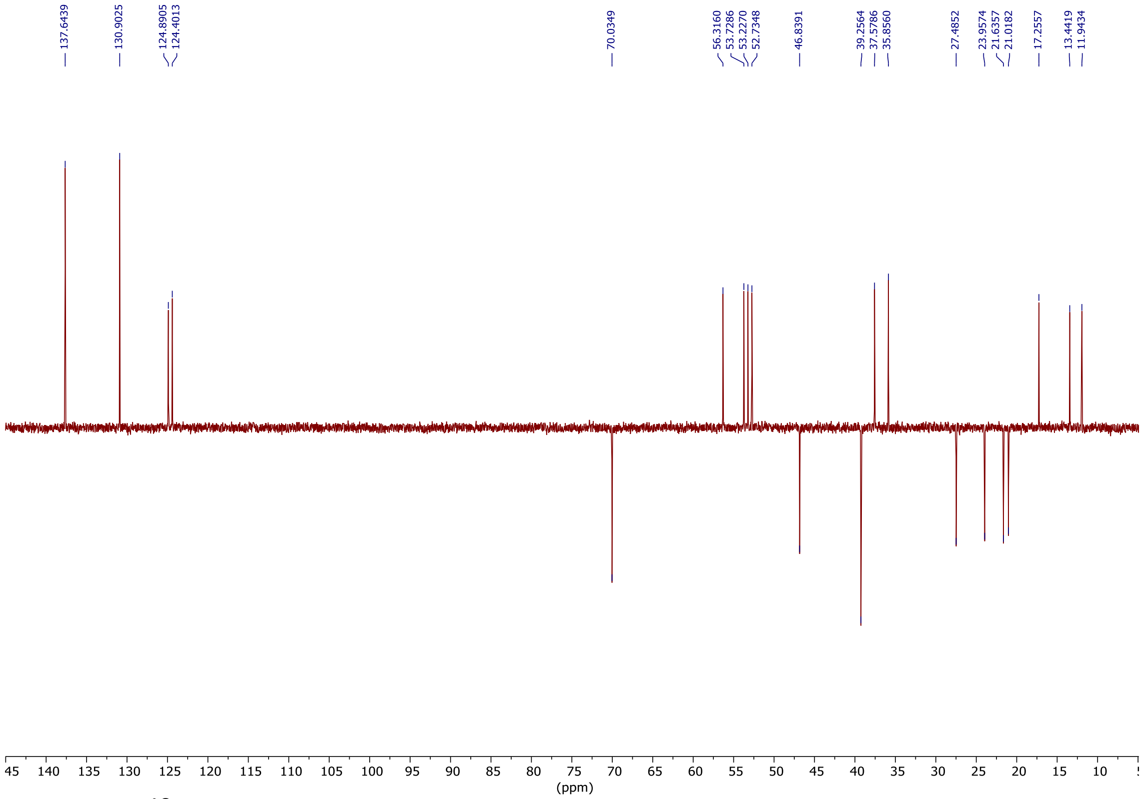

Figure S23. ^{13}C-DEPT 135 NMR spectrum of 5α-cholan-6-oxo-2-ene-23,24-dinor-22-yl 4-iodobenzoate (**27**).

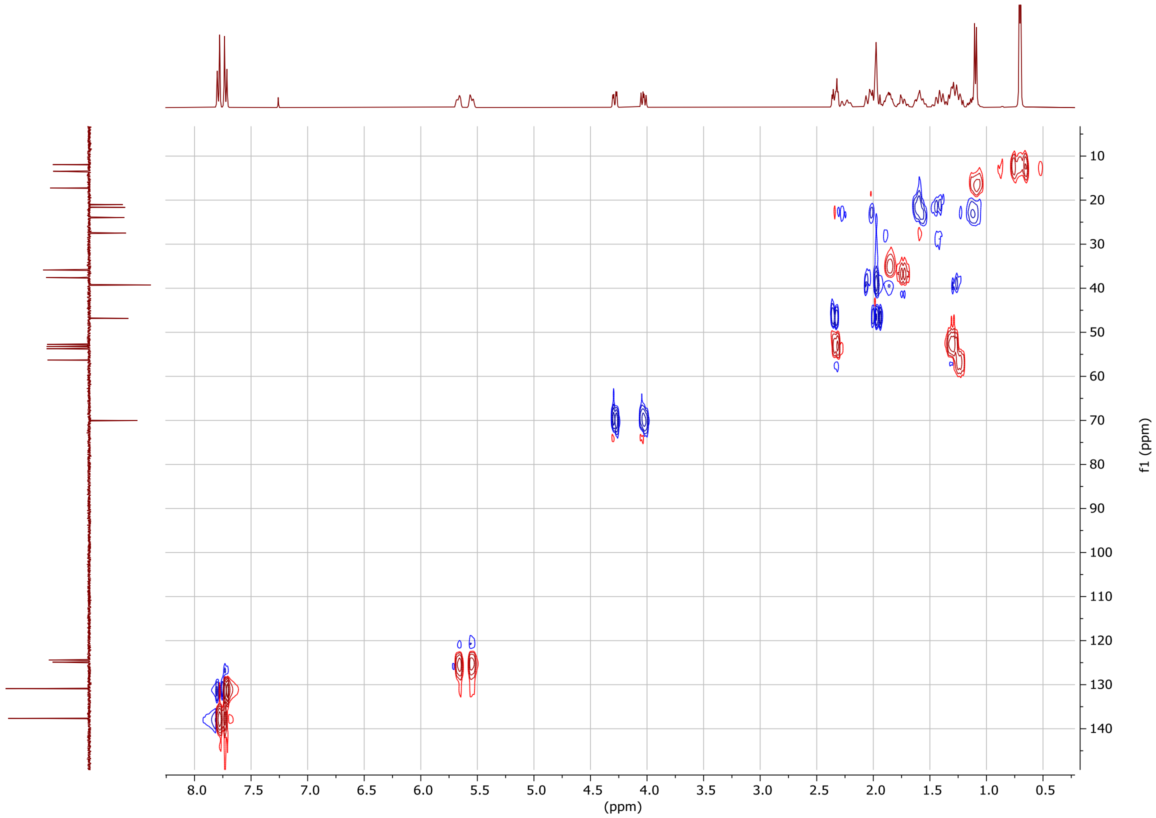

Figure S24. ^{1}H-^{13}C HSQC-ed. spectrum 5α-cholan-6-oxo-2-ene-23,24-dinor-22-yl 4-iodinebenzoate (**27**).

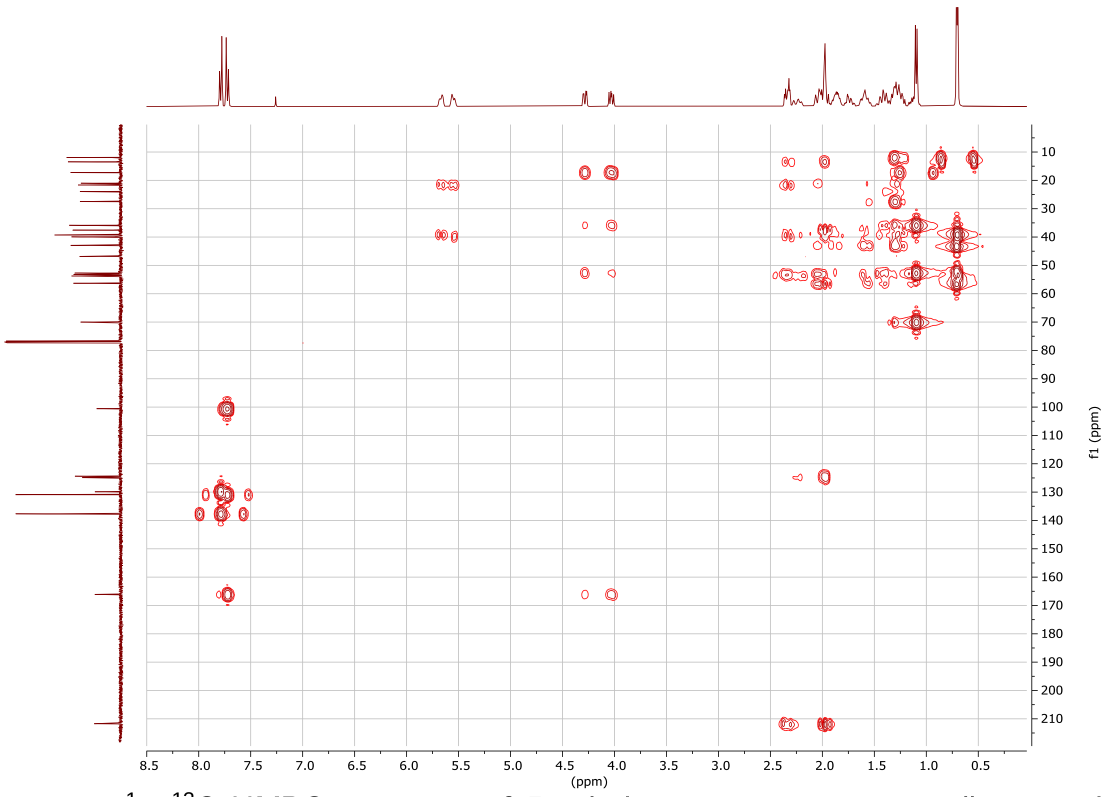

Figure S25. ^{1}H-^{13}C HMBC spectrum of 5α-cholan-6-oxo-2-ene-23,24-dinor-22-yl 4-iodobenzoate (**27**).

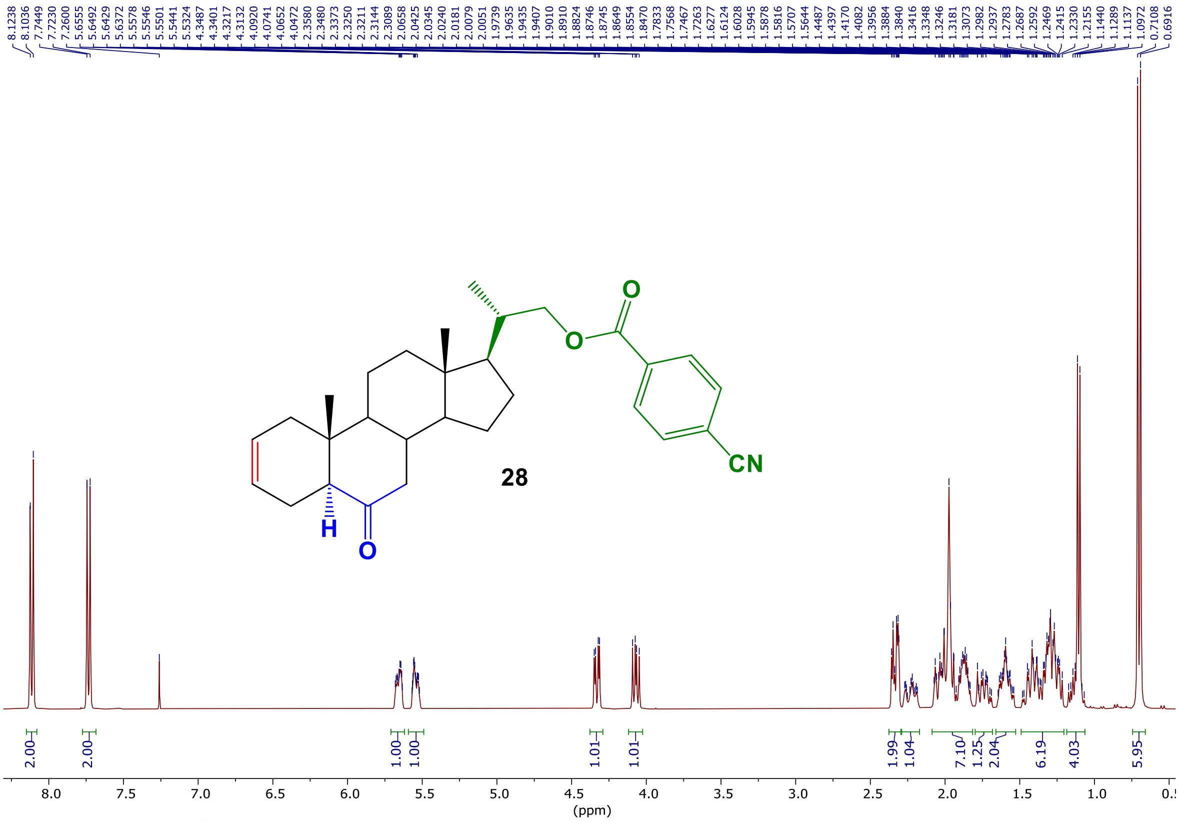

Figure S26. ^{1}H NMR spectrum of 5α-cholan-6-oxo-2-ene-23,24-dinor-22-yl 4-cyanobenzoate (**28**).

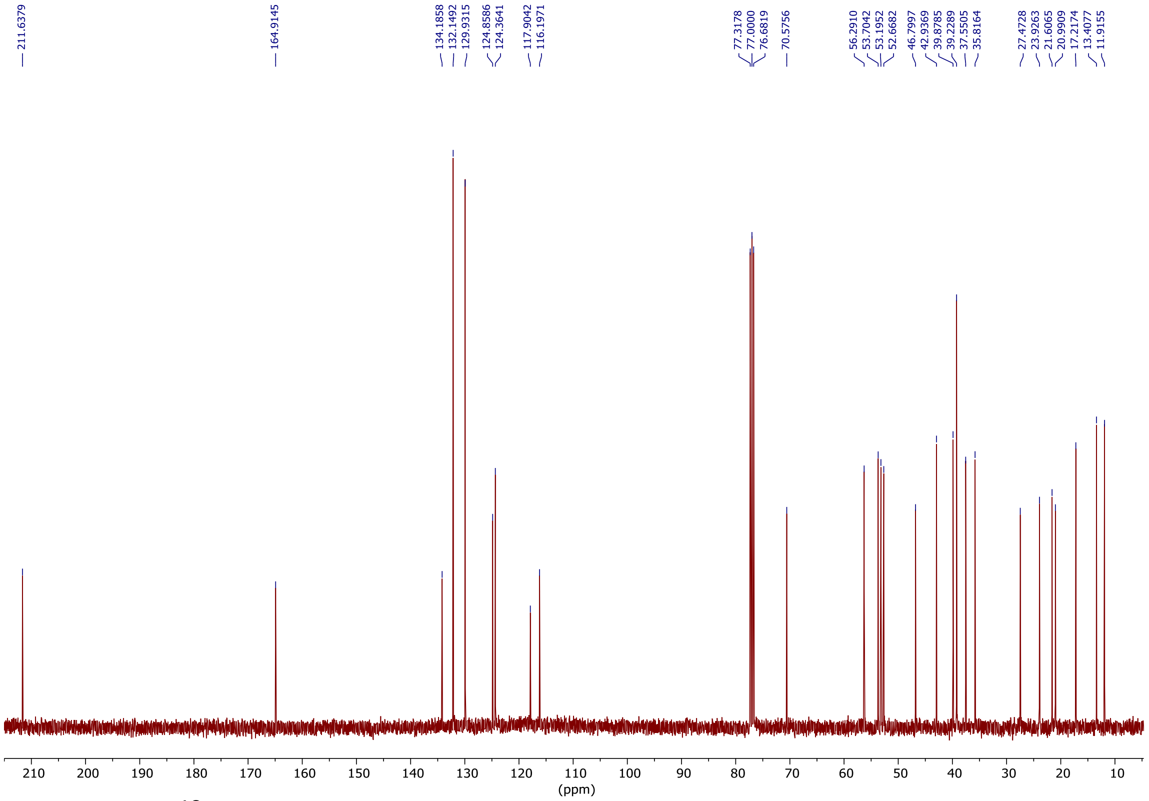

Figure S27. ^{13}C NMR spectrum of 5α-cholan-6-oxo-2-ene-23,24-dinor-22-yl 4-cyanobenzoate (**28**).

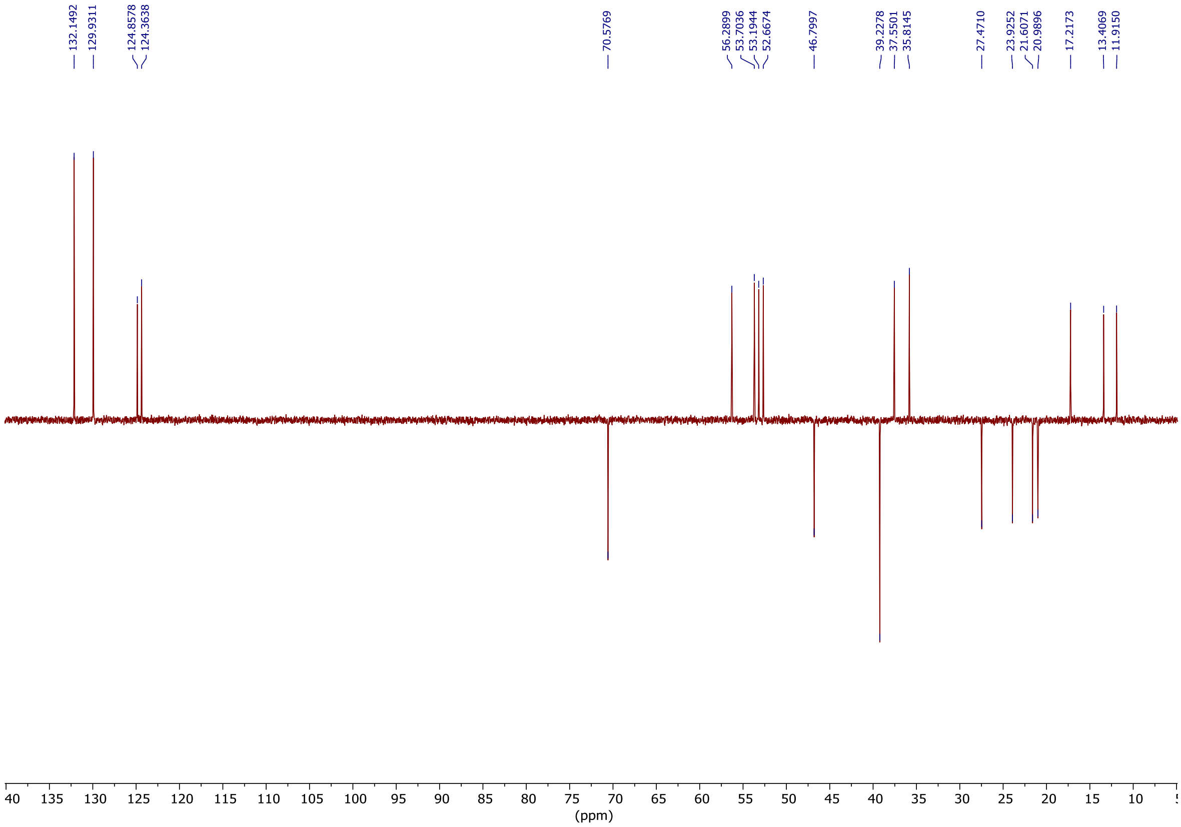

Figure S28. ^{13}C-DEPT 135 NMR spectrum of 5α-cholan-6-oxo-2-ene-23,24-dinor-22-yl 4-cyanobenzoate (**28**).

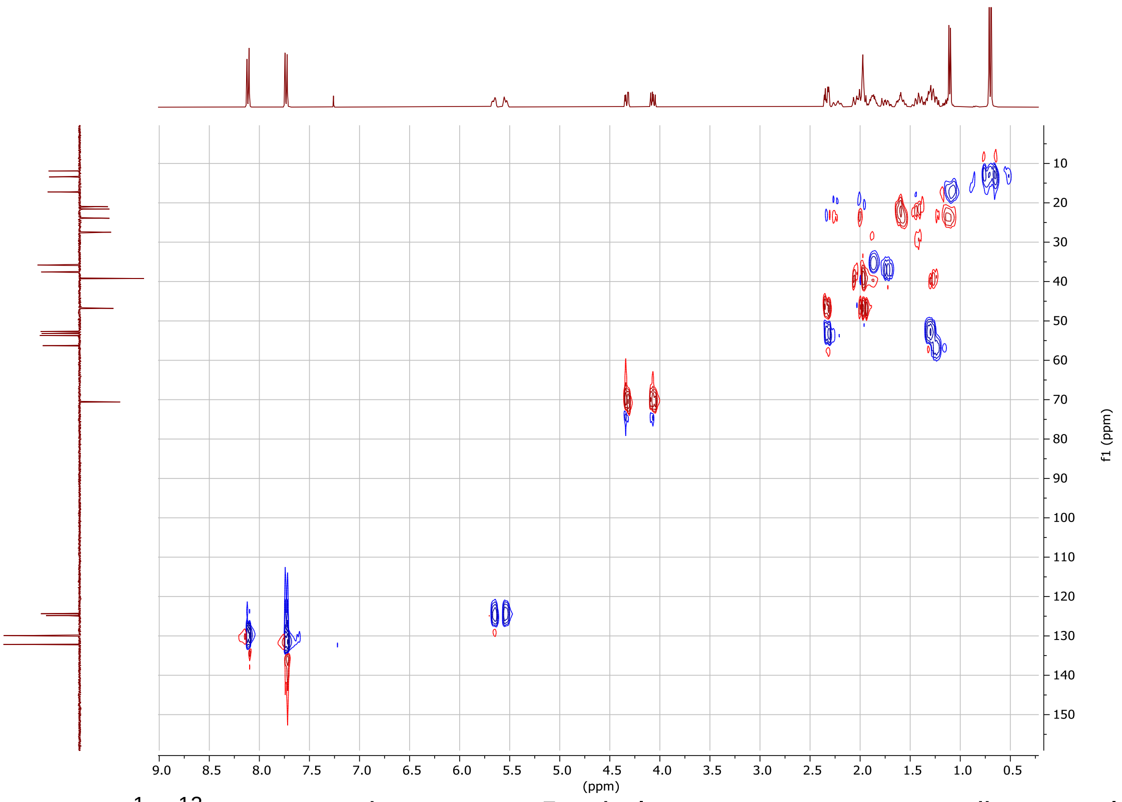

Figure S29. ^{1}H-^{13}C HSQC-ed. spectrum 5α-cholan-6-oxo-2-ene-23,24-dinor-22-yl 4-cyanobenzoate (**28**).

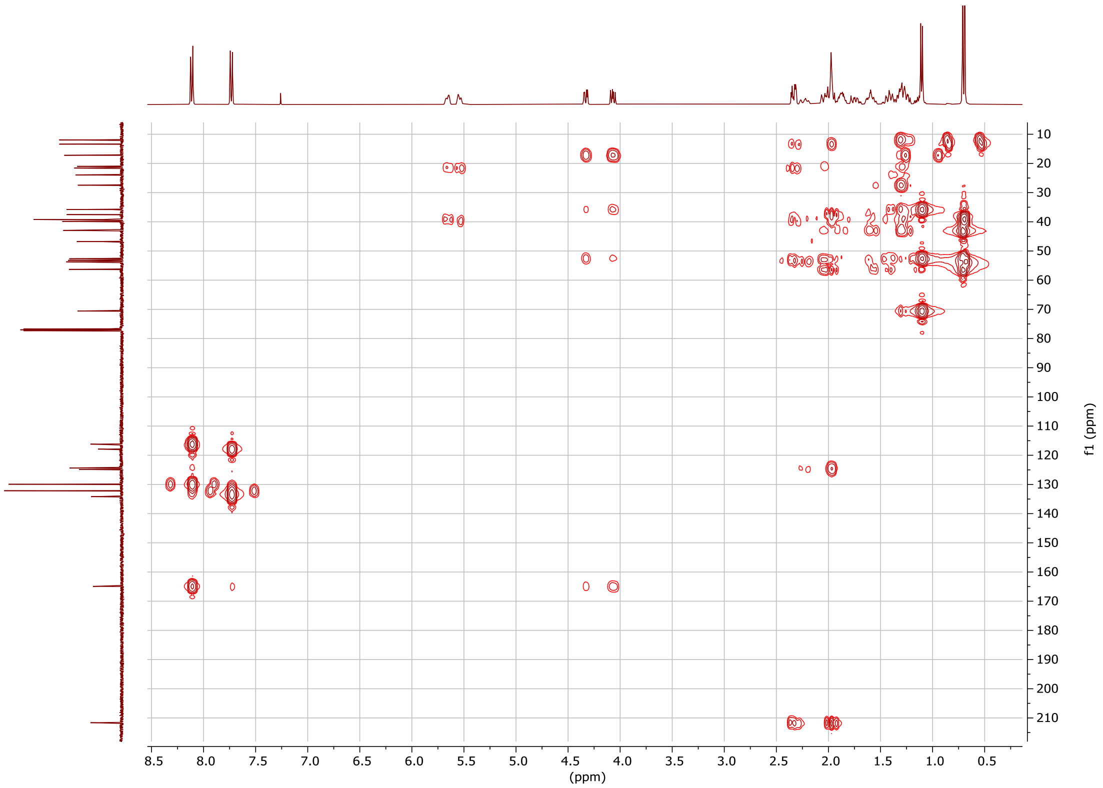

Figure S30. ^{1}H-^{13}C HMBC spectrum of 5α-cholan-6-oxo-2-ene-23,24-dinor-22-yl 4-cyanobenzoate (**28**).

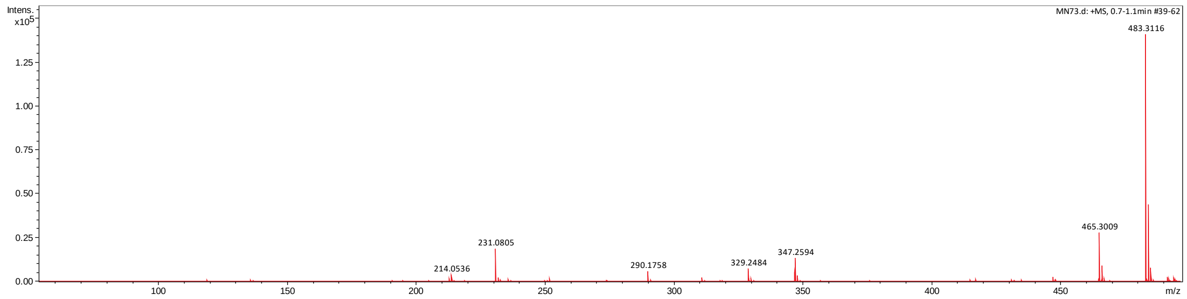

Figure S31. HRMS spectrum of 2α,3α-dihydroxy-5α-cholan-6-oxo-23,24-dinor-22-yl 4-methylbenzoate (**17**).

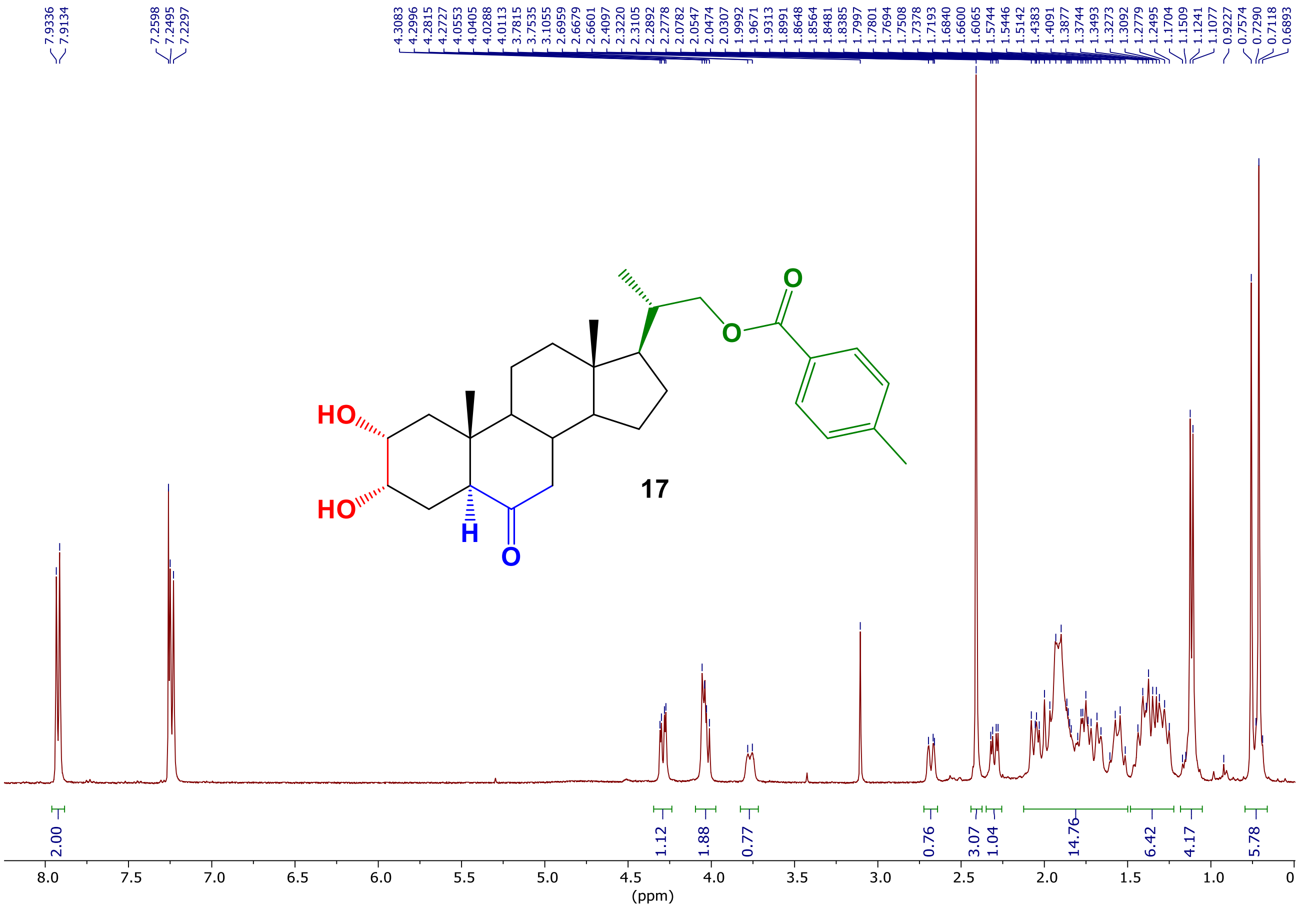

Figure S32. ^{1}H NMR spectrum of 2α,3α-dihydroxy-5α-cholan-6-oxo-23,24-dinor-22-yl 4-methylbenzoate (**17**).

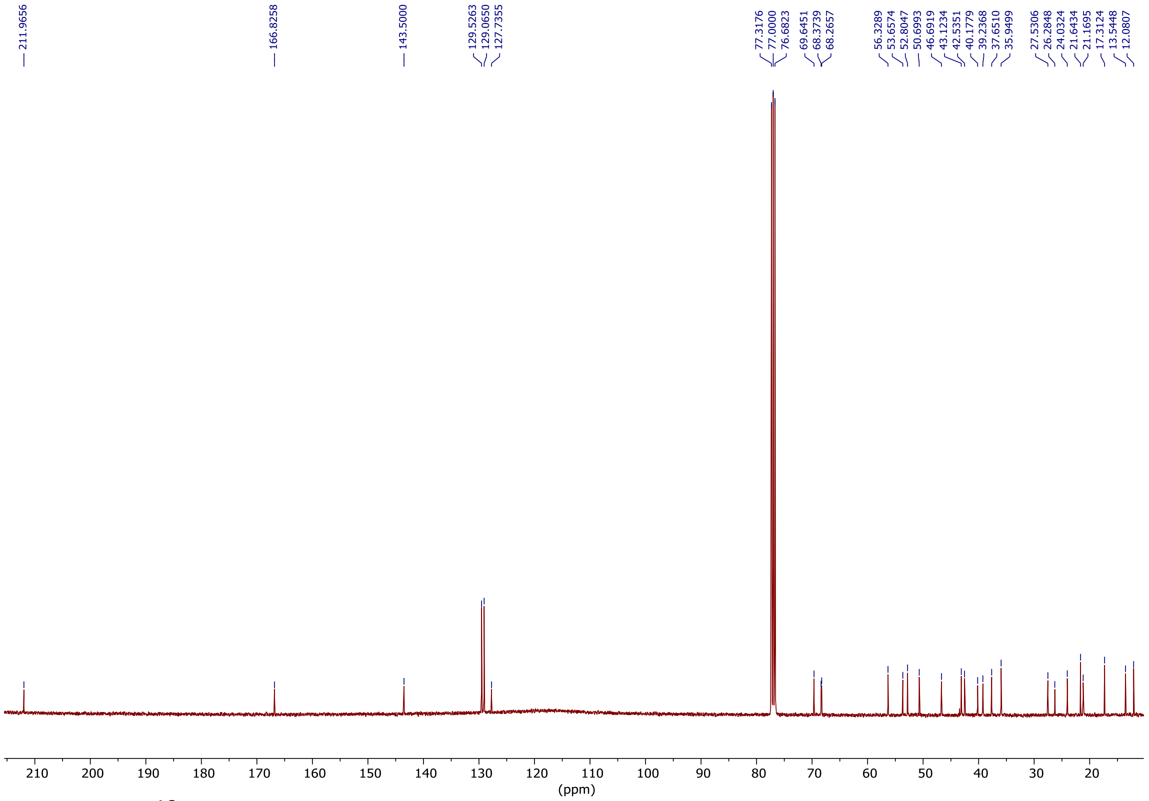

Figure S33. ^{13}C NMR spectrum of 2α,3α-dihydroxy-5α-cholan-6-oxo-23,24-dinor-22-yl 4-methylbenzoate (**17**).

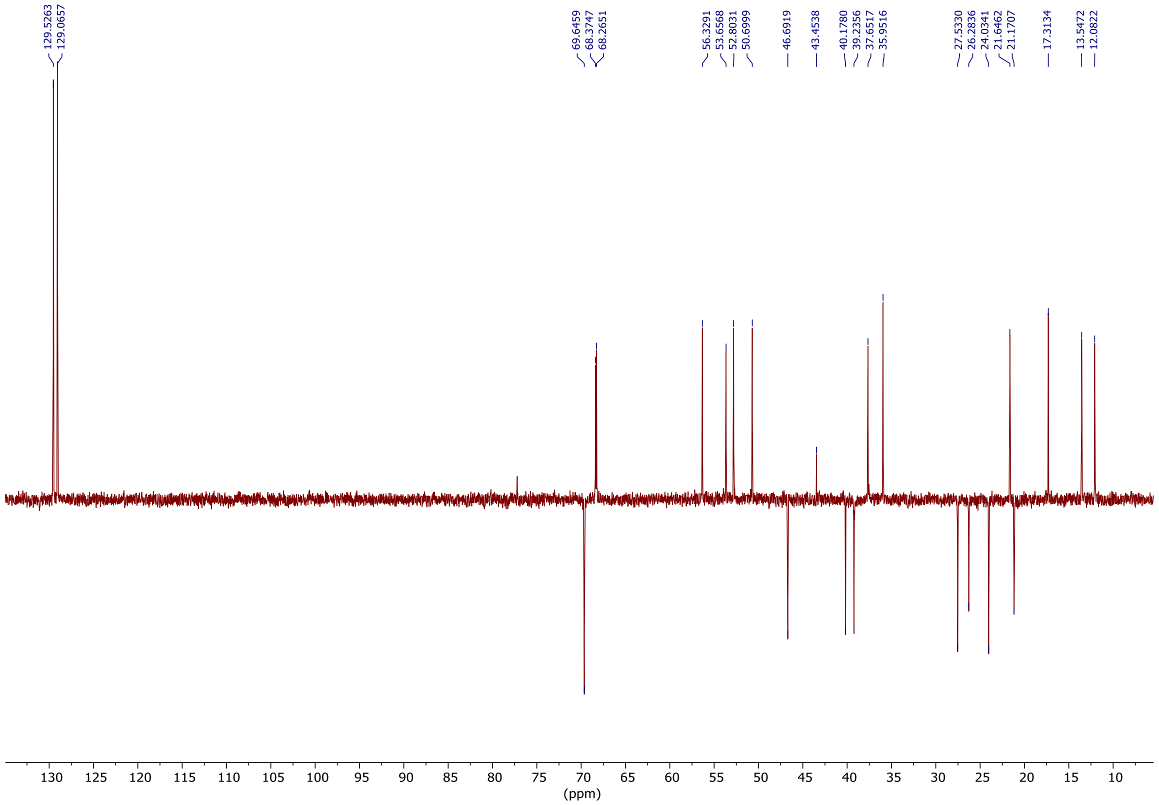

Figure S34. ^{13}C-DEPT 135 NMR spectrum of 2α,3α-dihydroxy-5α-cholan-6-oxo-23,24-dinor-22-yl 4-methylbenzoate (**17**).

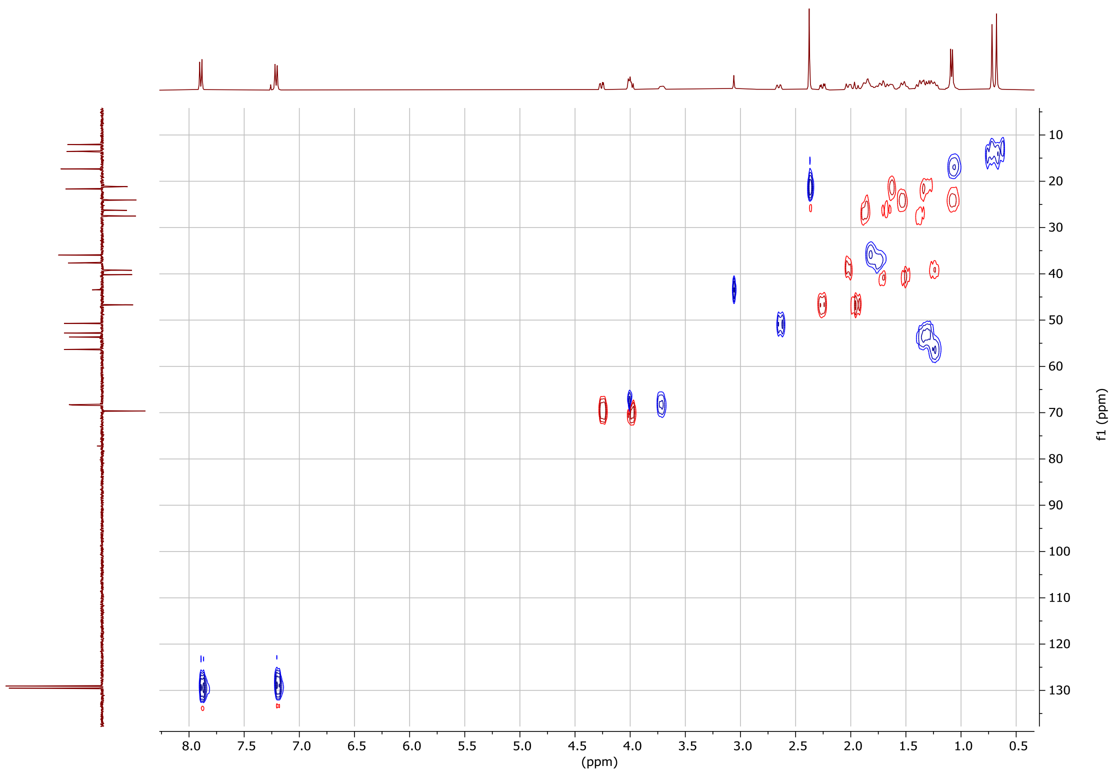

Figure S35. ^{1}H-^{13}C HSQC-ed. spectrum 2α,3α-dihydroxy-5α-cholan-6-oxo-23,24-dinor-22-yl 4-methylbenzoate (**17**).

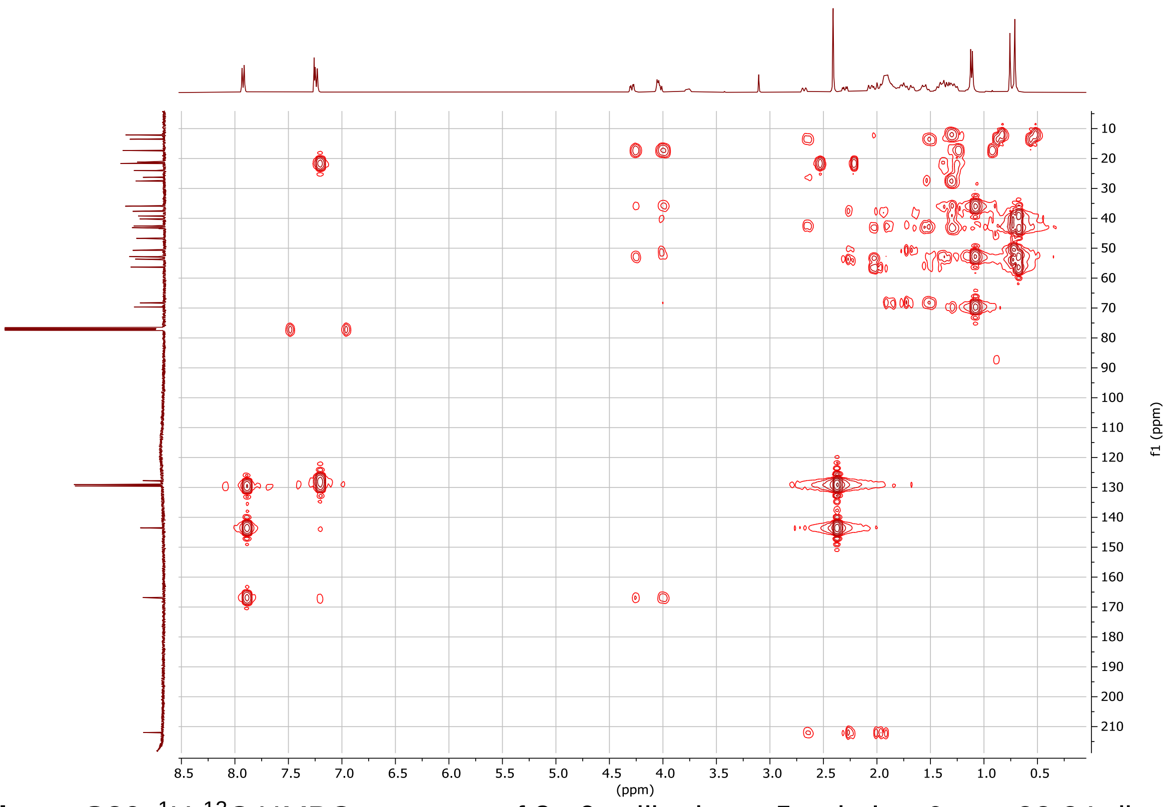

Figure S36. ^{1}H-^{13}C HMBC spectrum of 2α,3α-dihydroxy-5α-cholan-6-oxo-23,24-dinor-22-yl 4-methylbenzoate (**17**).

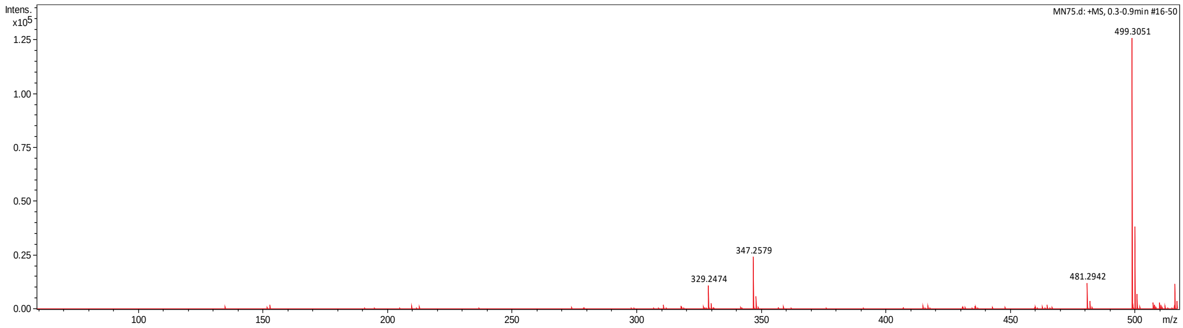

Figure S37. HRMS spectrum of 2α,3α-dihydroxy-5α-cholan-6-oxo-23,24-dinor-22-yl 4-methoxybenzoate (**18**).

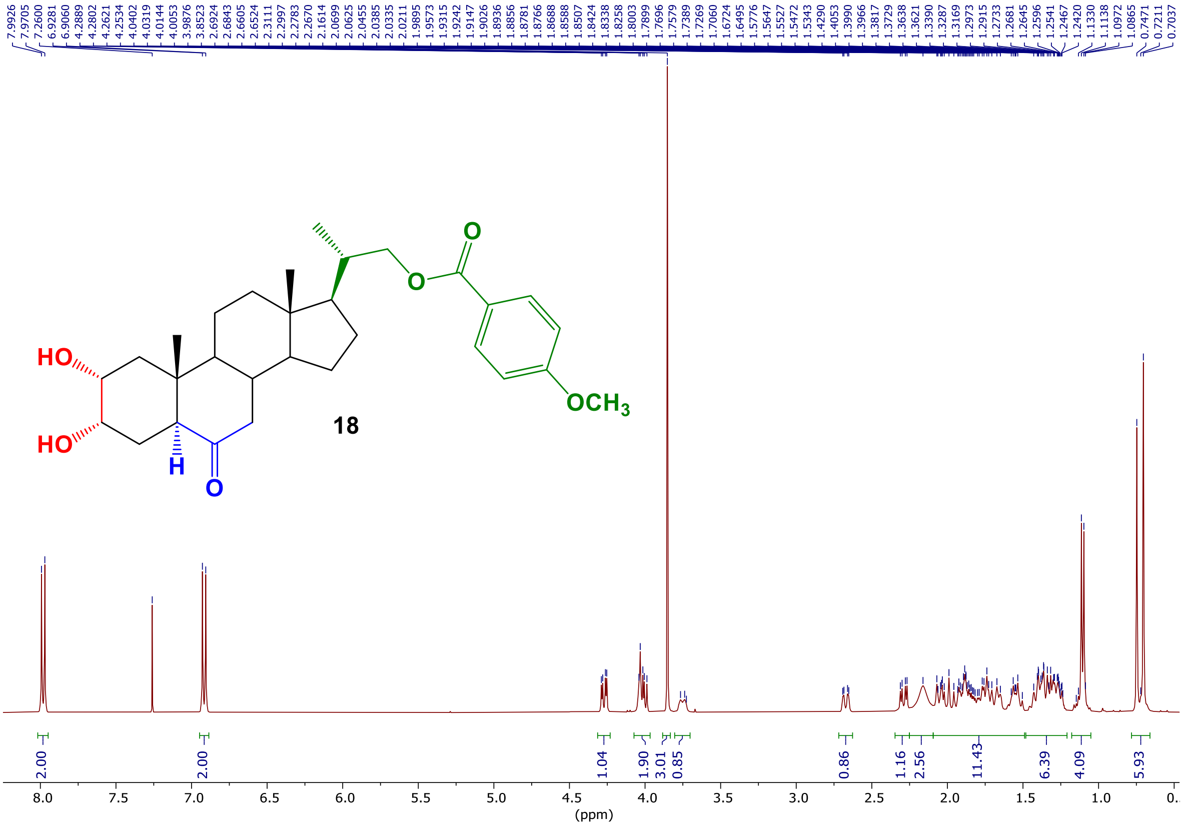

Figure S38. ^{1}H NMR spectrum of 2α,3α-dihydroxy-5α-cholan-6-oxo-23,24-dinor-22-yl 4-methoxybenzoate (**18**).

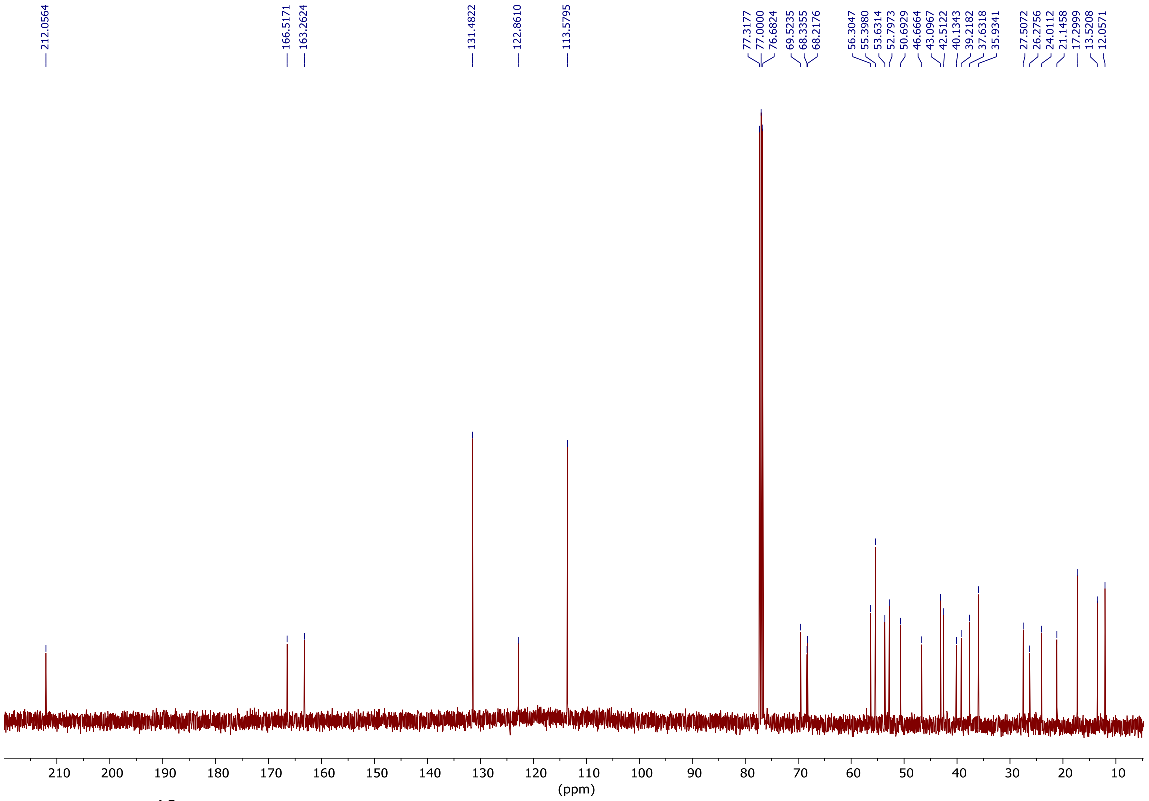

Figure S39. ^{13}C NMR spectrum of 2α,3α-dihydroxy-5α-cholan-6-oxo-23,24-dinor-22-yl 4-methoxybenzoate (**18**).

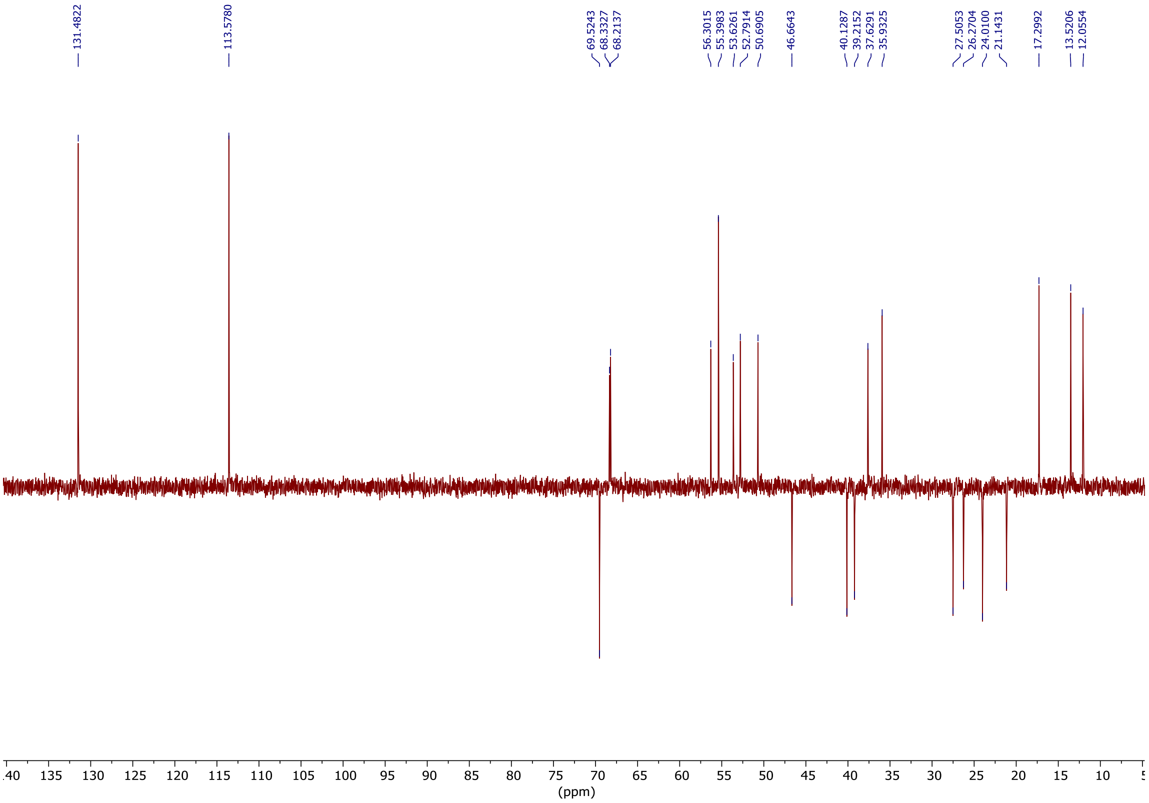

Figure S40. ^{13}C-DEPT 135 NMR spectrum of 2α,3α-dihydroxy-5α-cholan-6-oxo-23,24-dinor-22-yl 4-methoxybenzoate (**18**).

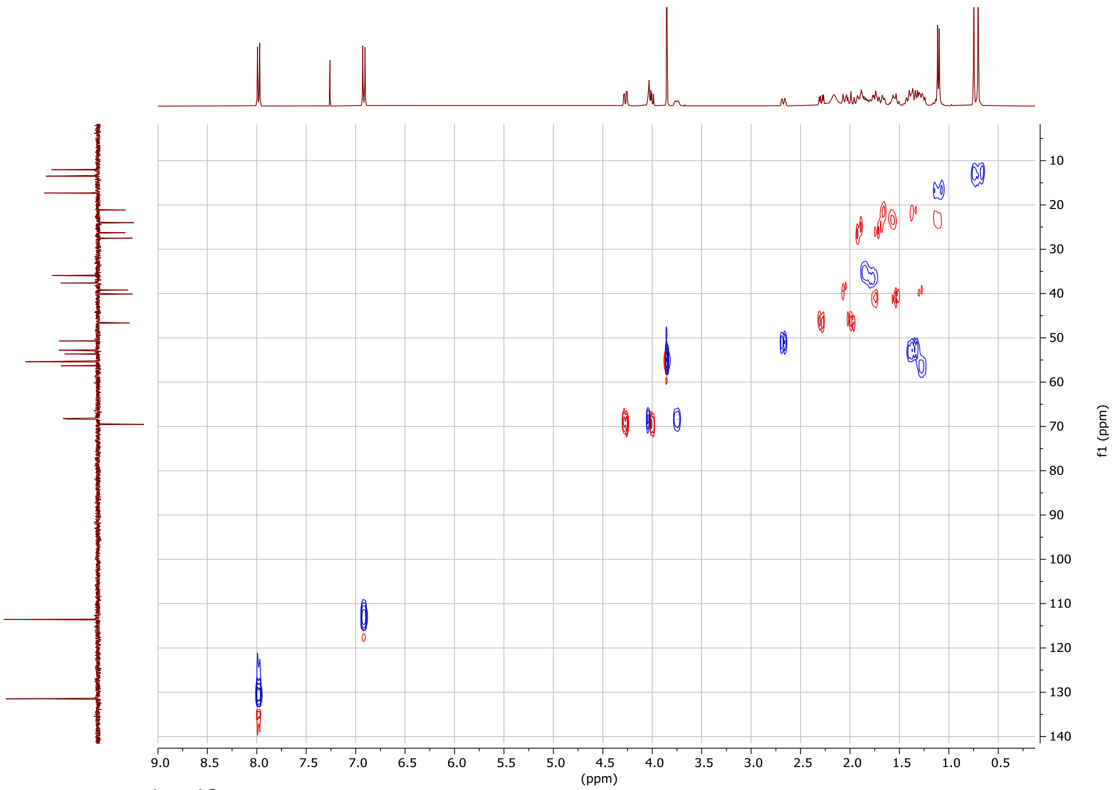

Figure S41. ^{1}H-^{13}C HSQC-ed. spectrum 2α,3α-dihydroxy-5α-cholan-6-oxo-23,24-dinor-22-yl 4-methoxybenzoate (**18**).

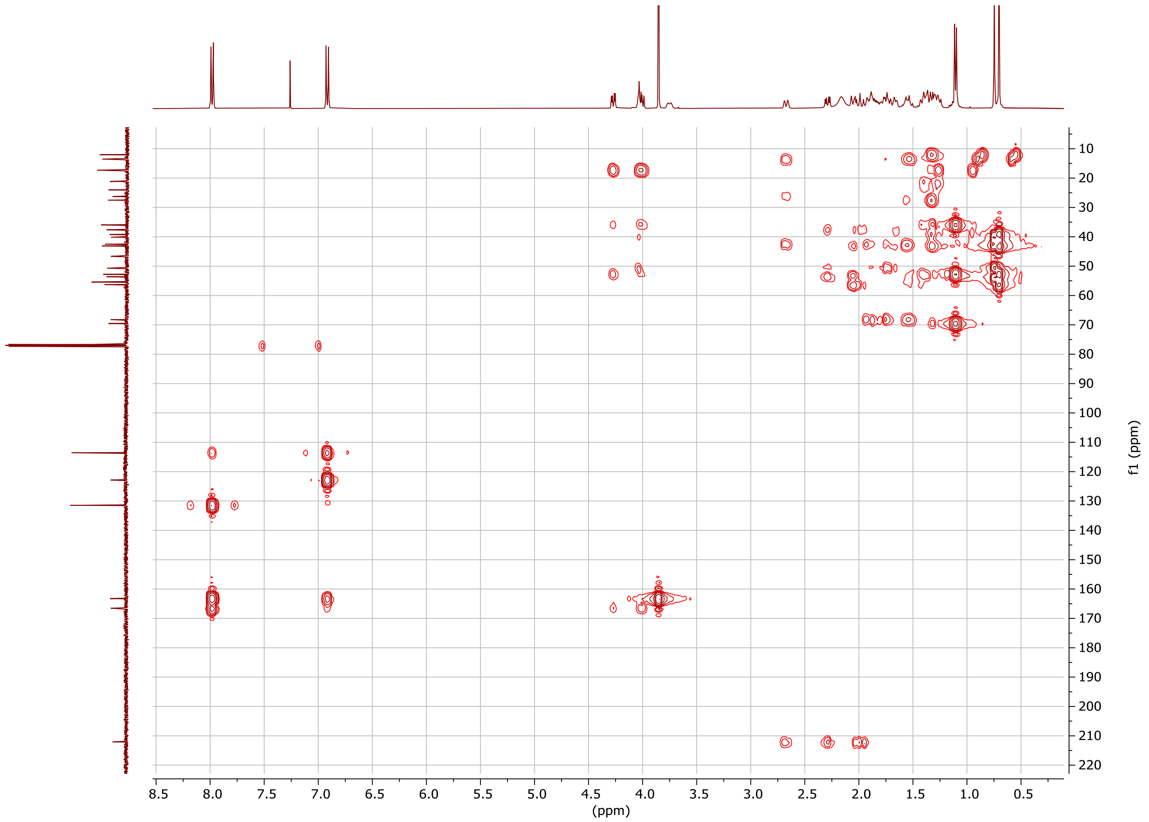

Figure S42. ^{1}H-^{13}C HMBC spectrum of 2α,3α-dihydroxy-5α-cholan-6-oxo-23,24-dinor-22-yl 4-methoxybenzoate (**18**).

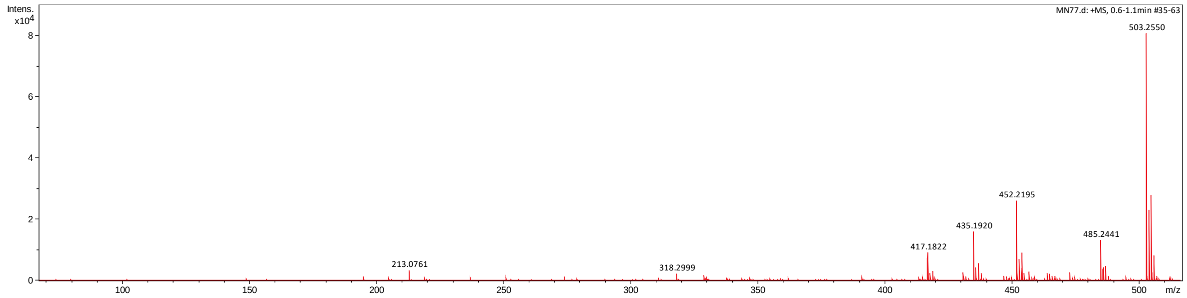

Figure S43. HRMS spectrum of 2α,3α-dihydroxy-5α-cholan-6-oxo-23,24-dinor-22-yl 4-chlorobenzoate (**19**).

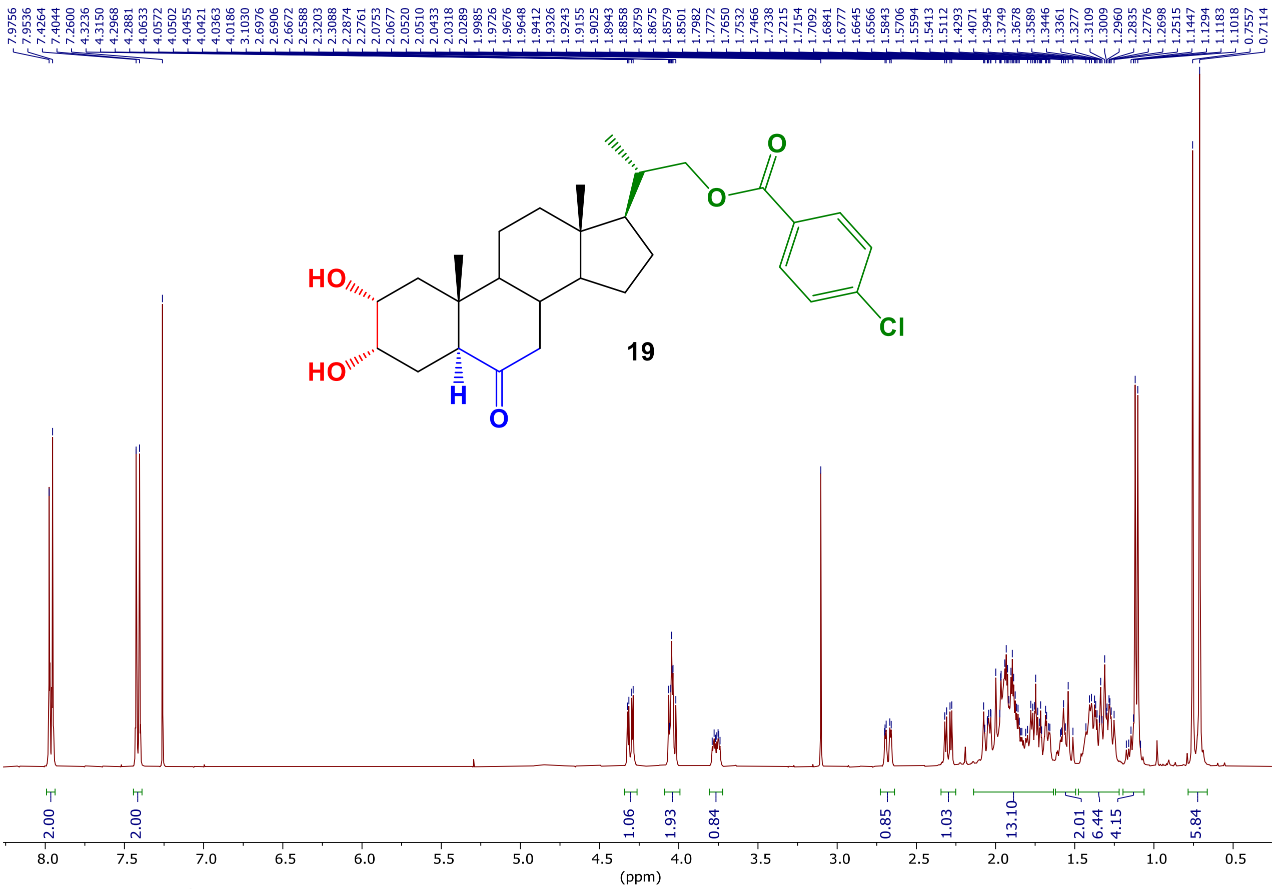

Figure S44. ^{1}H NMR spectrum of 2α,3α-dihydroxy-5α-cholan-6-oxo-23,24-dinor-22-yl 4-chlorobenzoate (**19**).

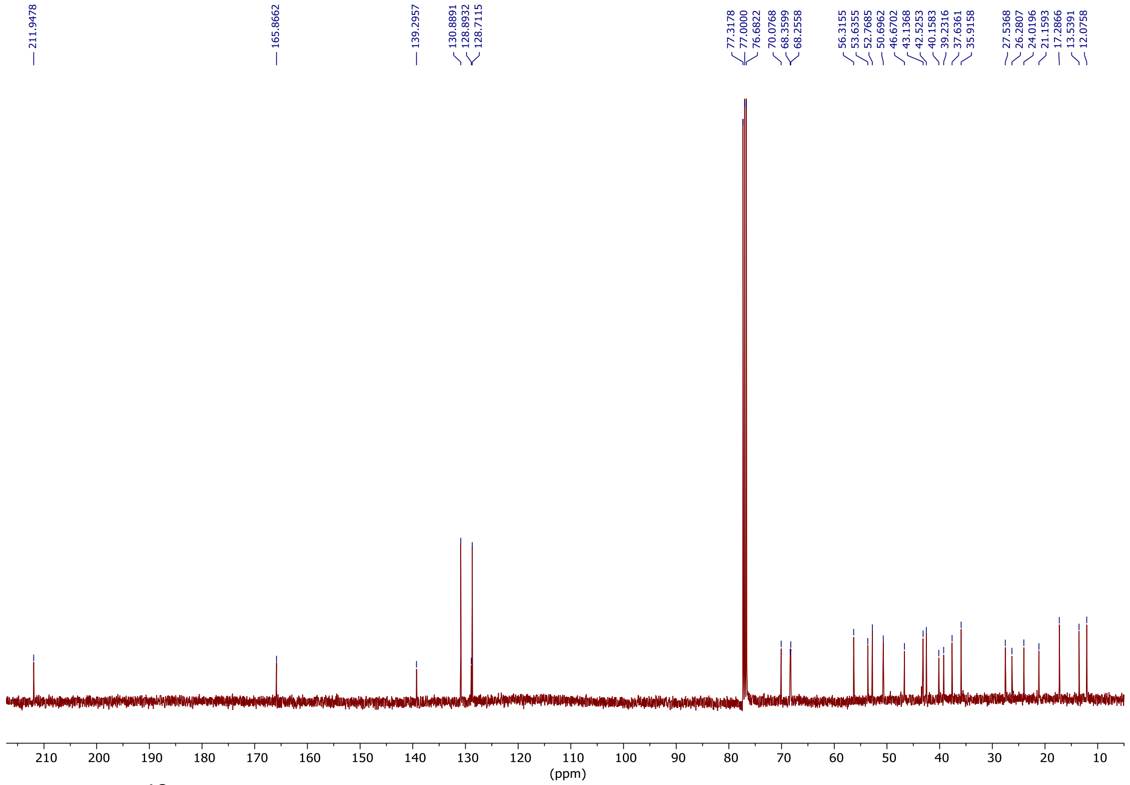

Figure S45. ^{13}C NMR spectrum of 2α,3α-dihydroxy-5α-cholan-6-oxo-23,24-dinor-22-yl 4-chlorobenzoate (**19**).

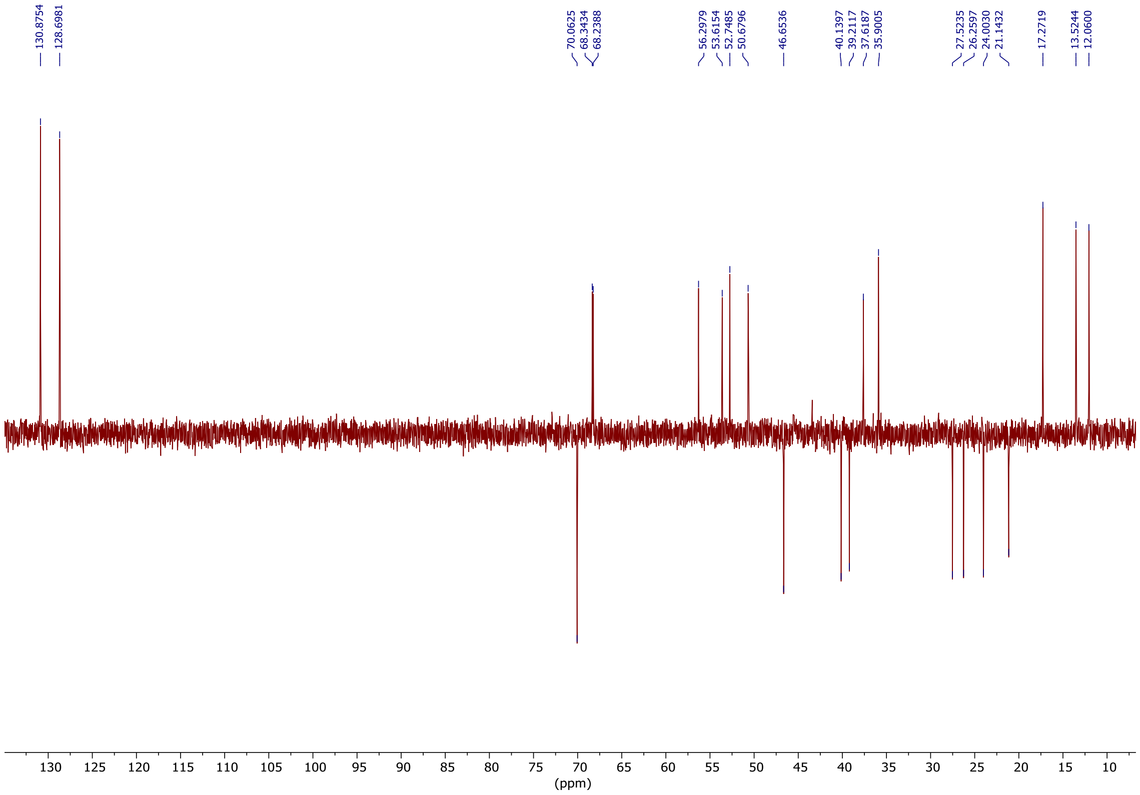

Figure S46. ^{13}C-DEPT 135 NMR spectrum of 2α,3α-dihydroxy-5α-cholan-6-oxo-23,24-dinor-22-yl 4-chlorobenzoate (**19**).

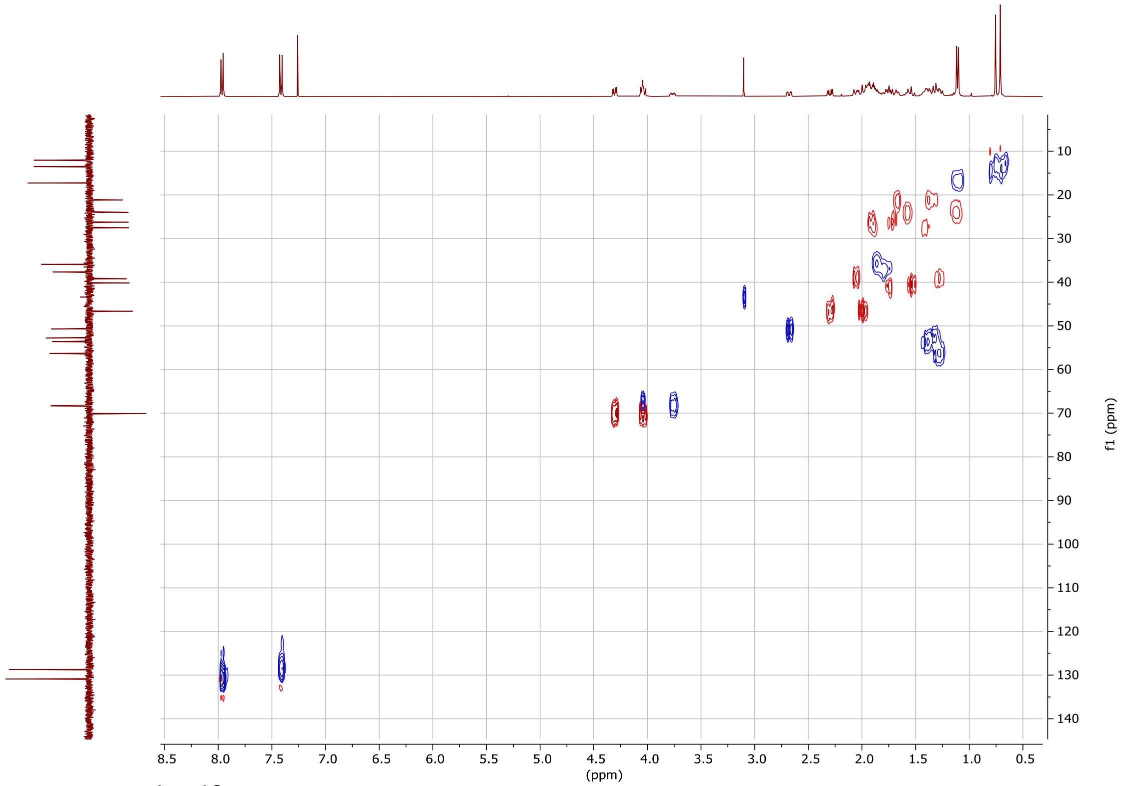

Figure S47. ^{1}H-^{13}C HSQC-ed. spectrum 2α,3α-dihydroxy-5α-cholan-6-oxo-23,24-dinor-22-yl 4-chlorobenzoate (**19**).

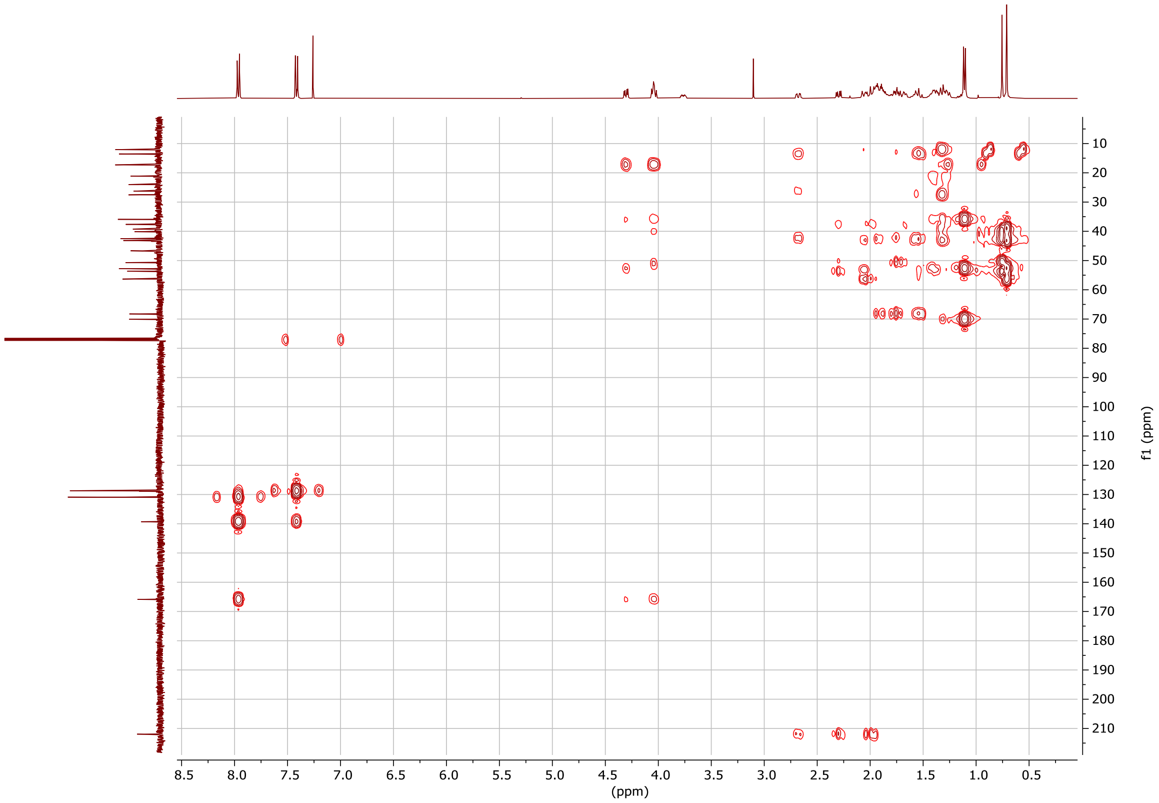

Figure S48. ^{1}H-^{13}C HMBC spectrum of 2α,3α-dihydroxy-5α-cholan-6-oxo-23,24-dinor-22-yl 4-chlorobenzoate (**19**).

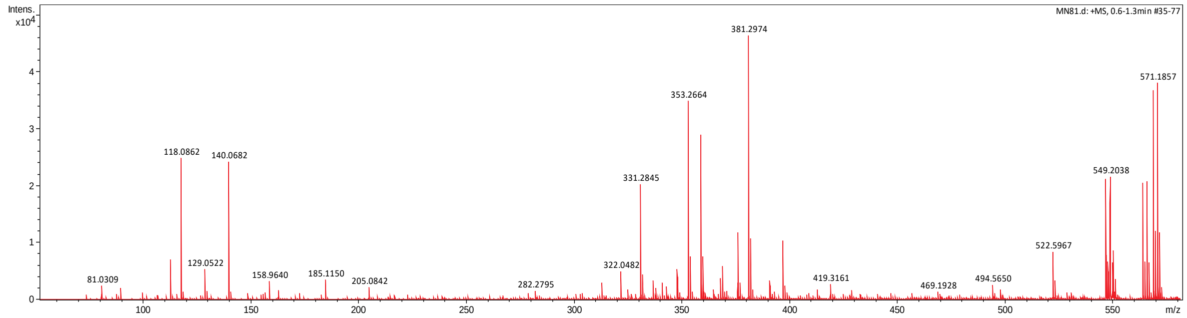

Figure S49. HRMS spectrum of 2α,3α-dihydroxy-5α-cholan-6-oxo-23,24-dinor-22-yl 4-bromobenzoate (**20**).

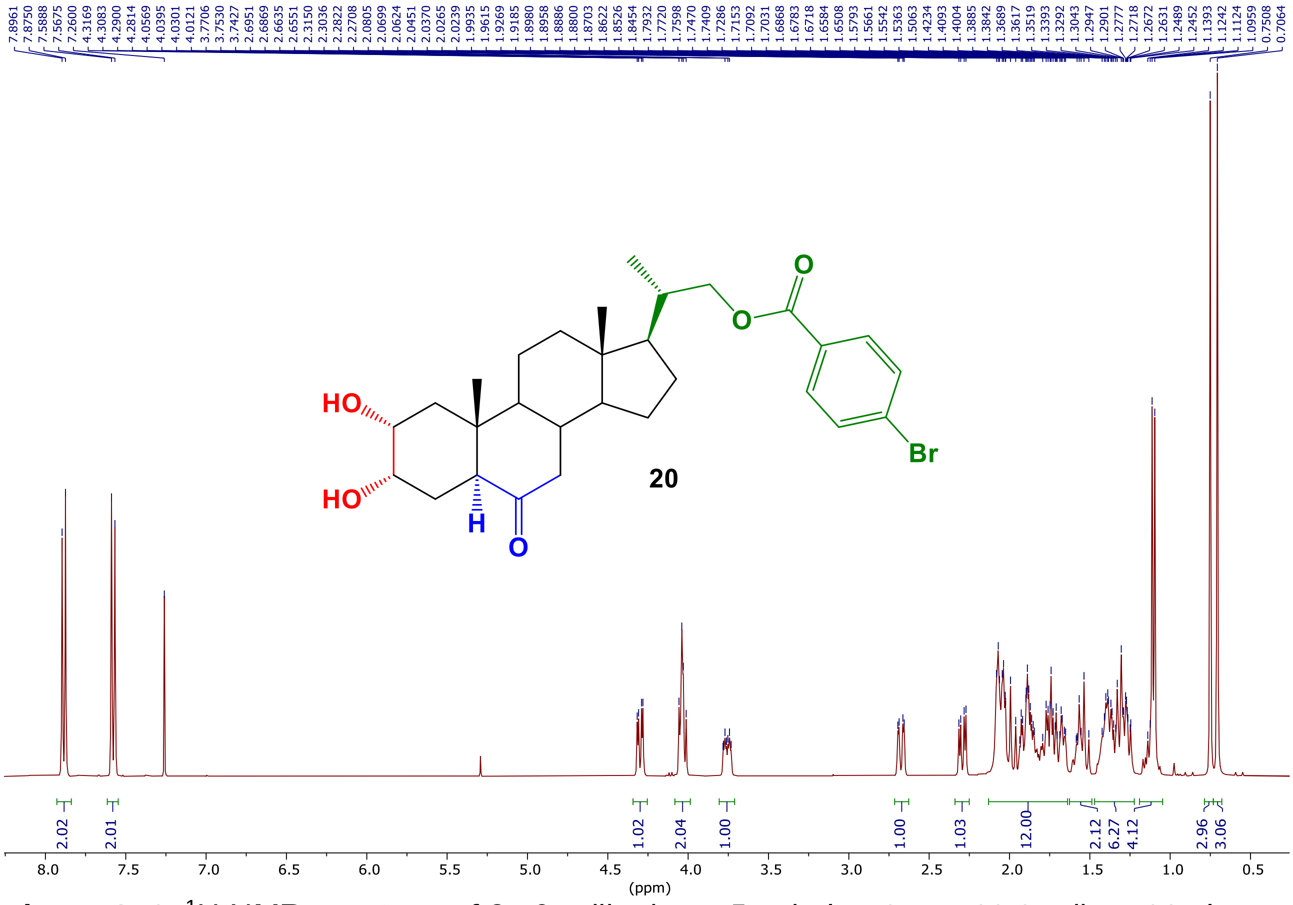

Figure S50. ^{1}H NMR spectrum of 2α,3α-dihydroxy-5α-cholan-6-oxo-23,24-dinor-22-yl 4-bromobenzoate (**20**).

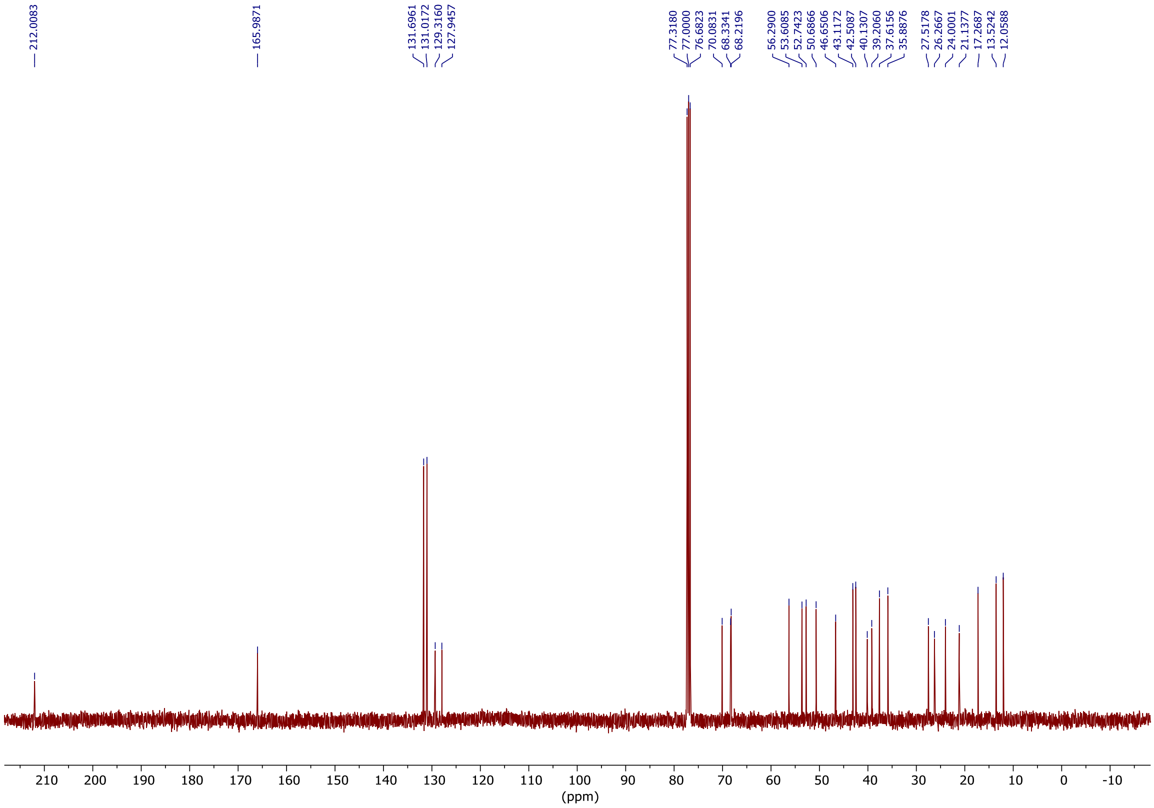

Figure S51. ^{13}C NMR spectrum of 2α,3α-dihydroxy-5α-cholan-6-oxo-23,24-dinor-22-yl 4-bromobenzoate (**20**).

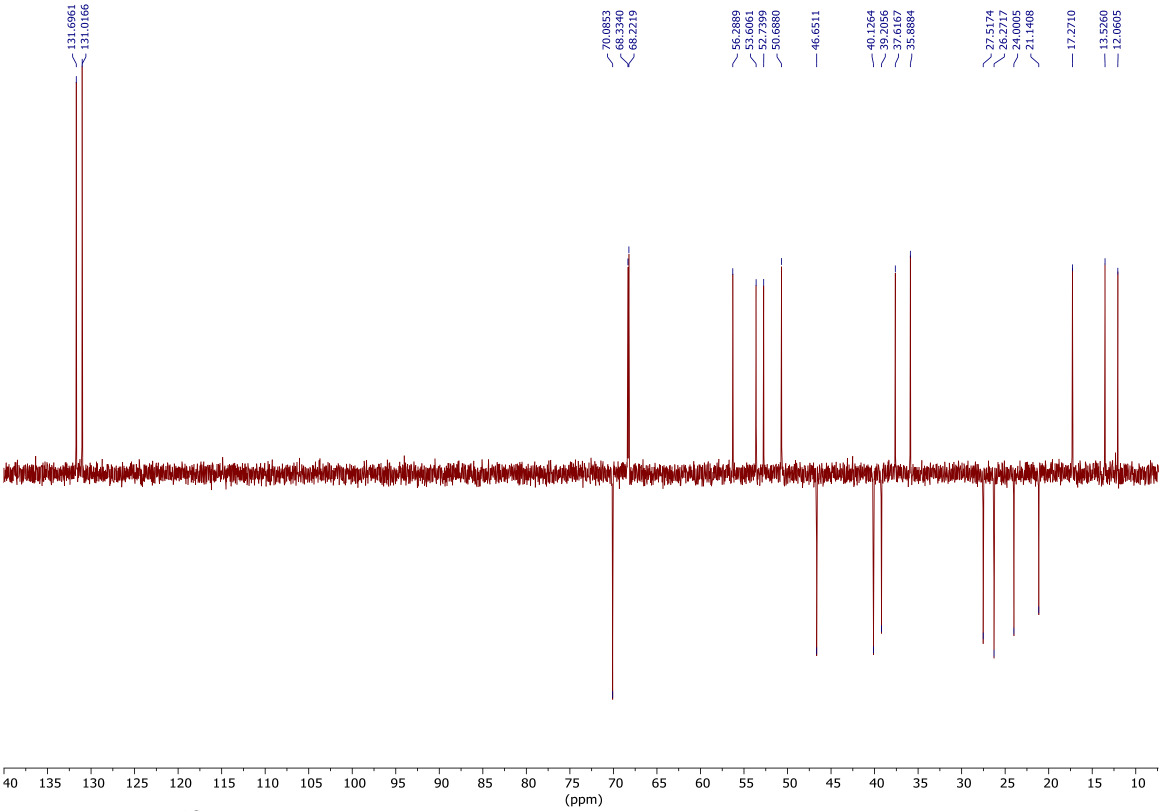

Figure S52. ^{13}C-DEPT 135 NMR spectrum of 2α,3α-dihydroxy-5α-cholan-6-oxo-23,24-dinor-22-yl 4-bromobenzoate (**20**).

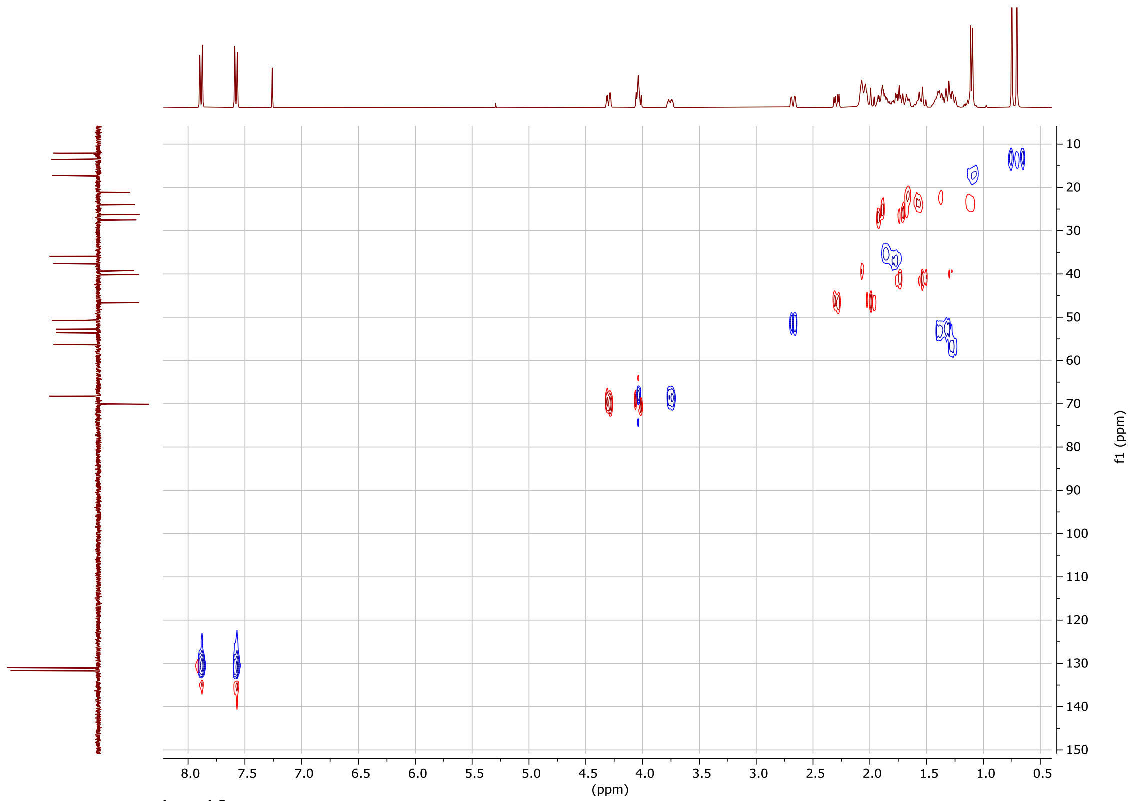

Figure S53. ^{1}H-^{13}C HSQC-ed. spectrum 2α,3α-dihydroxy-5α-cholan-6-oxo-23,24-dinor-22-yl 4-bromobenzoate (**20**).

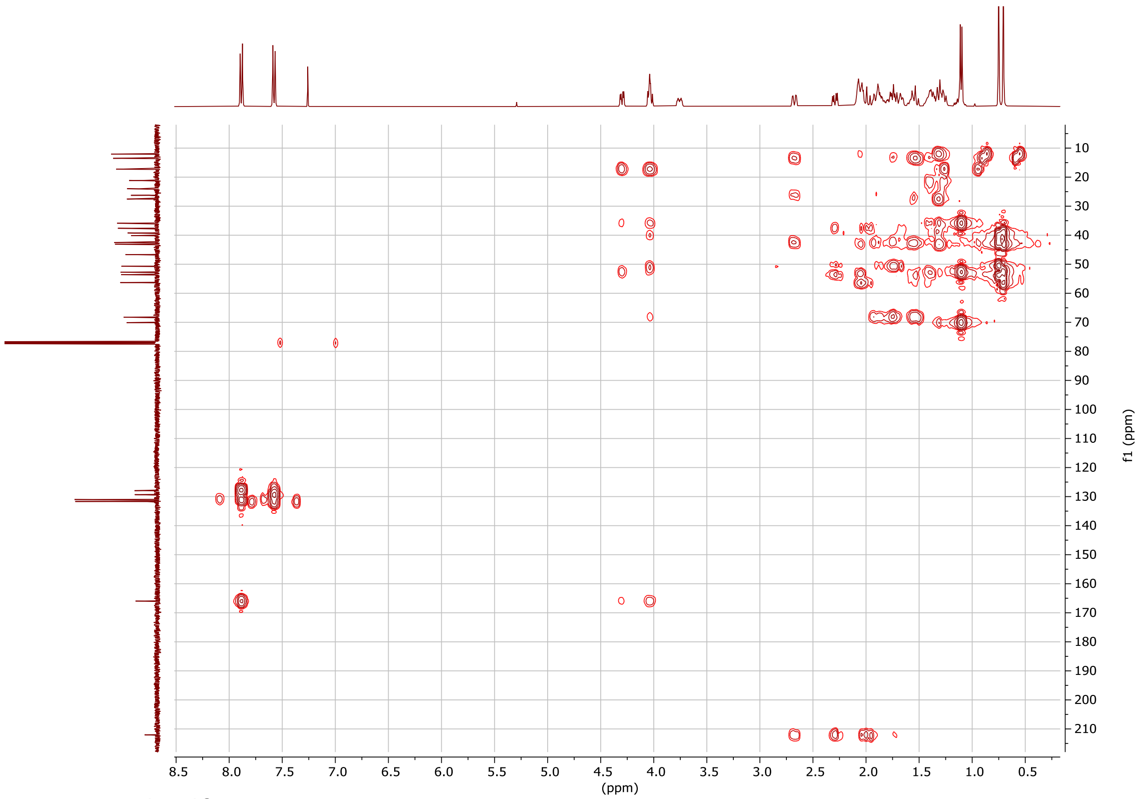

Figure S54. ^{1}H-^{13}C HMBC spectrum of 2α,3α-dihydroxy-5α-cholan-6-oxo-23,24-dinor-22-yl 4-bromobenzoate (**20**).

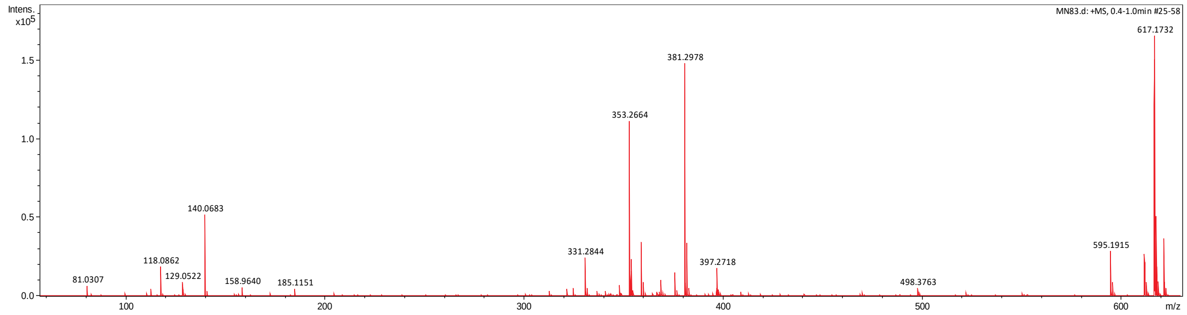

Figure S55. HRMS spectrum of 2α,3α-dihydroxy-5α-cholan-6-oxo-23,24-dinor-22-yl 4-iodobenzoate (**21**).

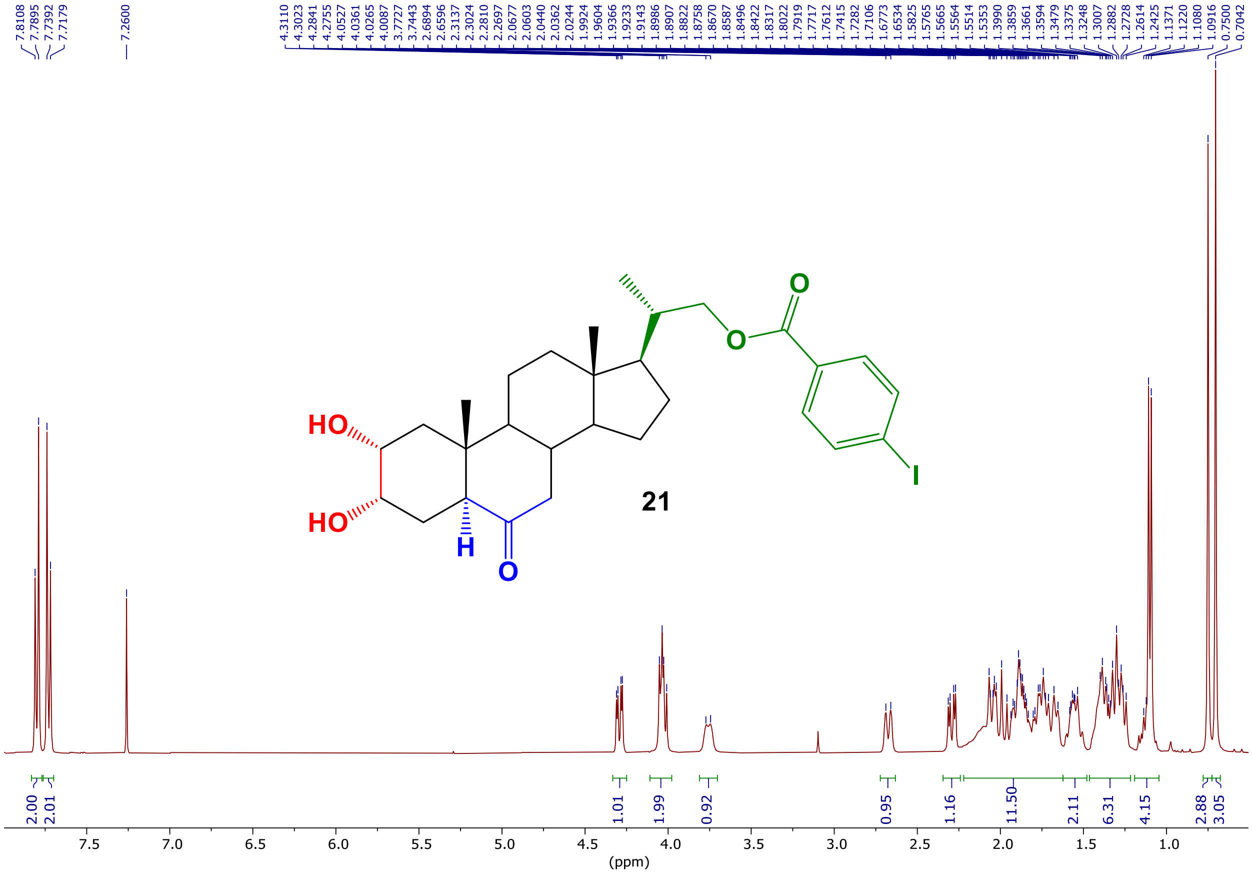

Figure S56. ^{1}H NMR spectrum of 2α,3α-dihydroxy-5α-cholan-6-oxo-23,24-dinor-22-yl 4-iodobenzoate (**21**).

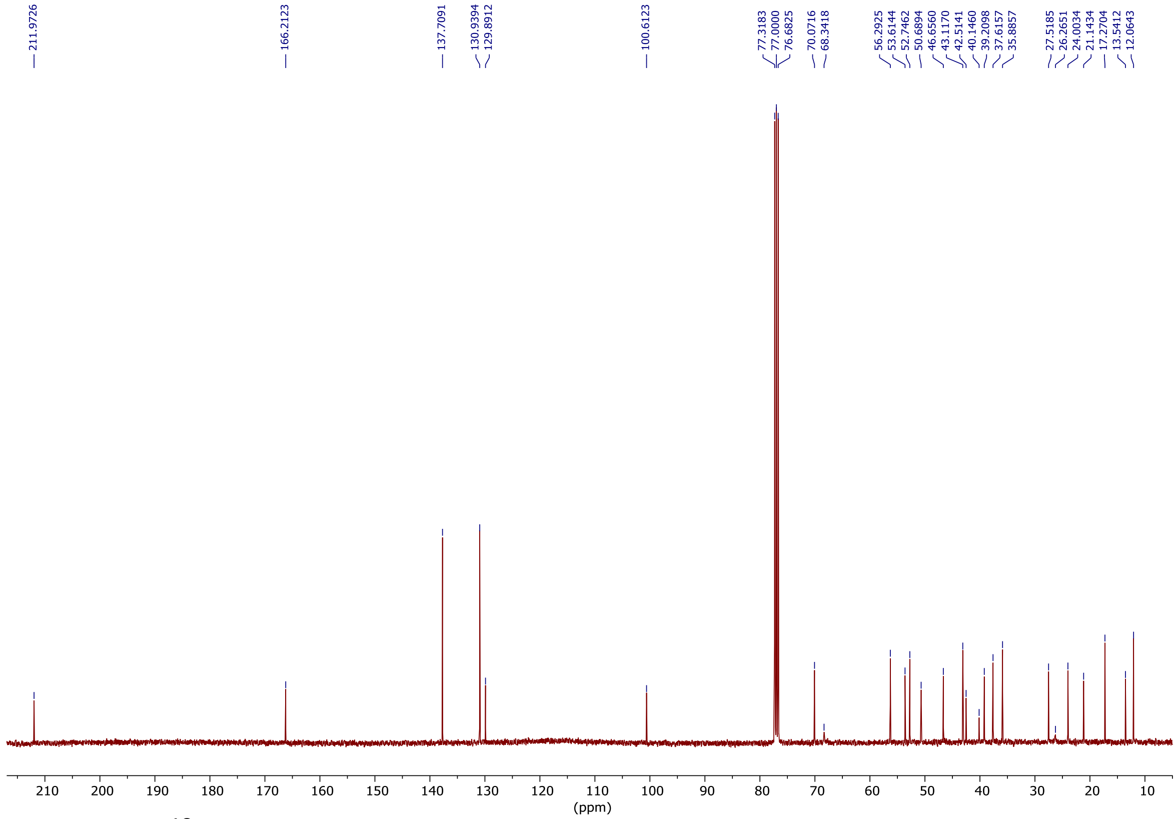

Figure S57. ^{13}C NMR spectrum of 2α,3α-dihydroxy-5α-cholan-6-oxo-23,24-dinor-22-yl 4-iodobenzoate (**21**).

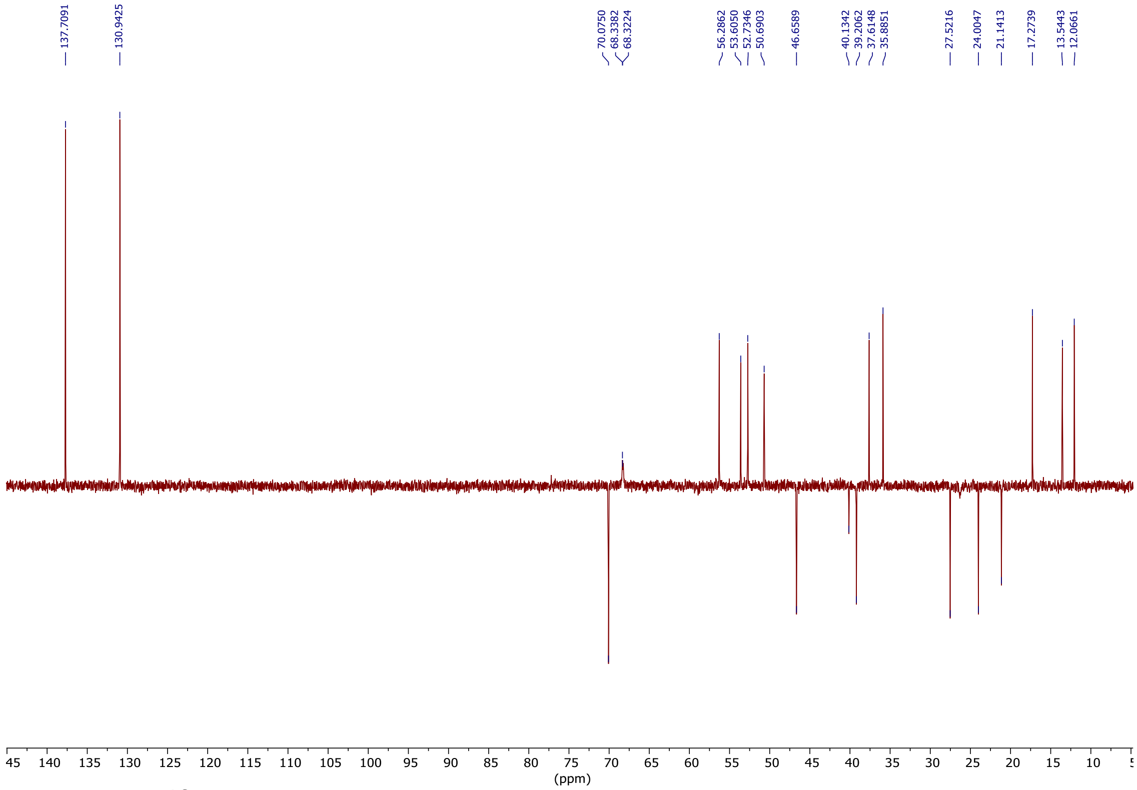

Figure S58. ^{13}C-DEPT 135 NMR spectrum of 2α,3α-dihydroxy-5α-cholan-6-oxo-23,24-dinor-22-yl 4-iodobenzoate (**21**).

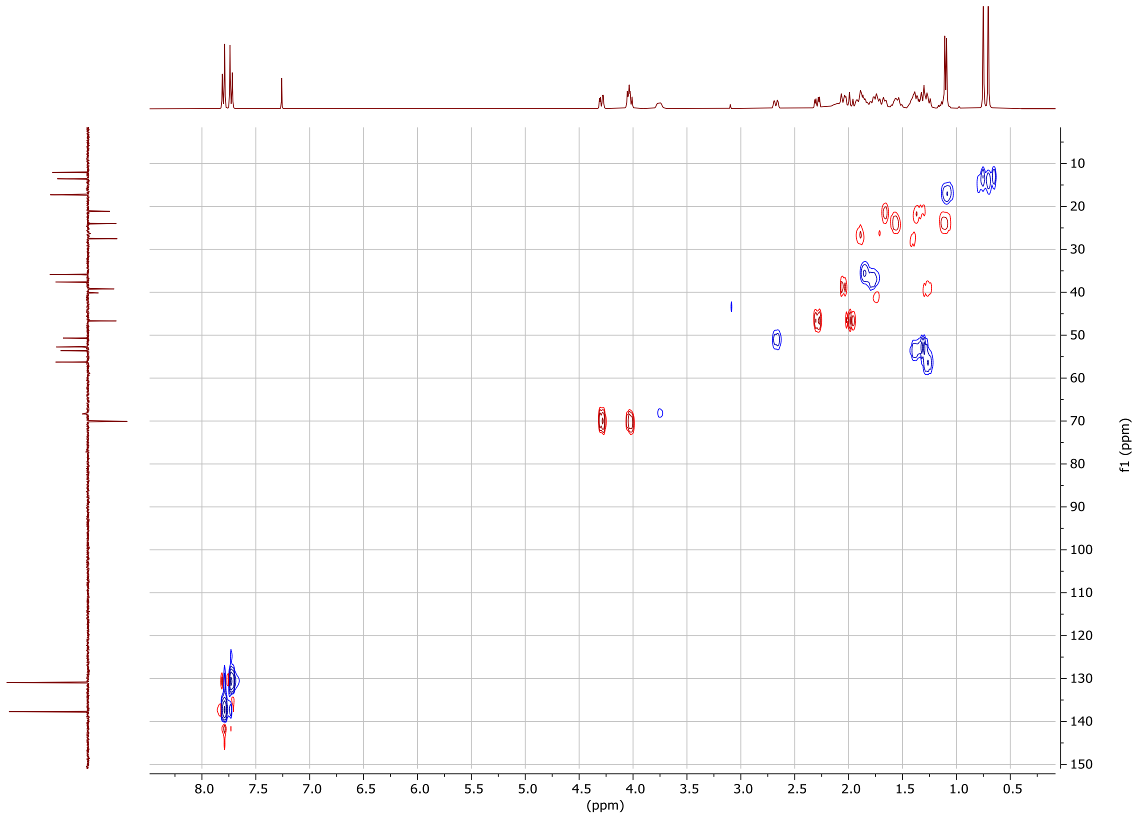

Figure S59. ¹H-¹³C HSQC-ed. spectrum 2α,3α-dihydroxy-5α-cholan-6-oxo-23,24-dinor-22-yl 4-iodobenzoate (**21**).

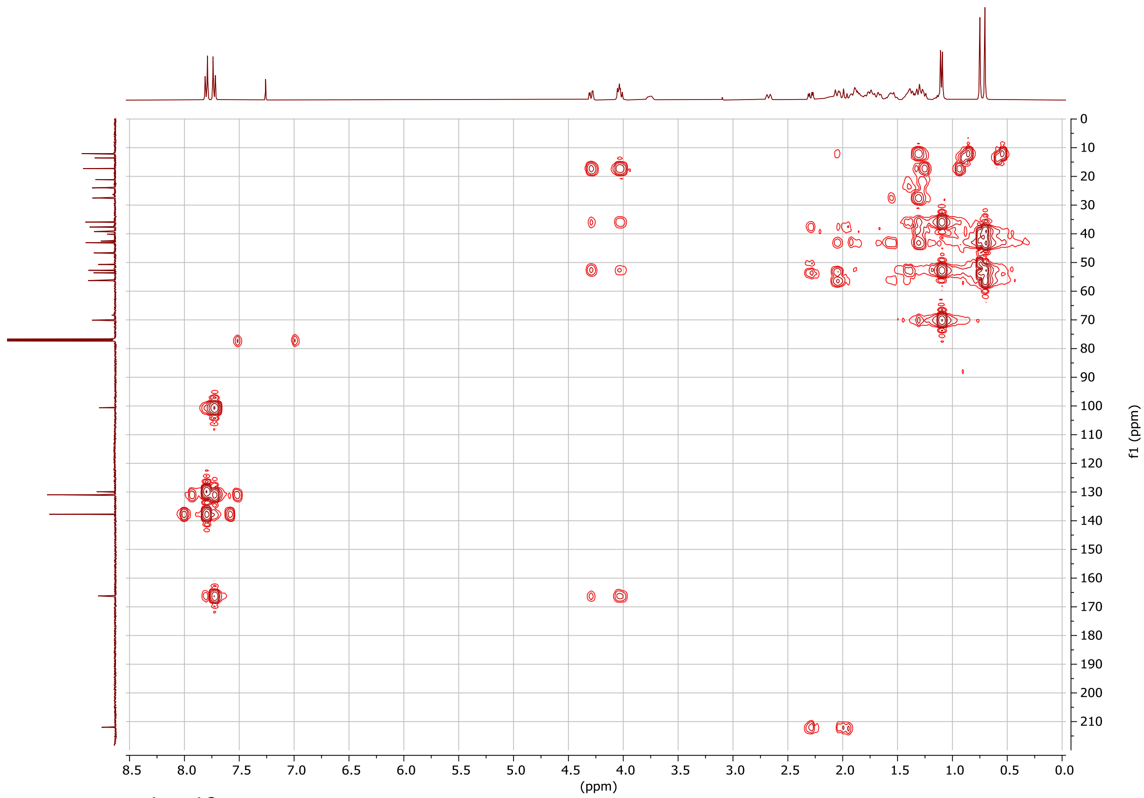

Figure S60. ¹H-¹³C HMBC spectrum of 2α,3α-dihydroxy-5α-cholan-6-oxo-23,24-dinor-22-yl 4-iodobenzoate (**21**).

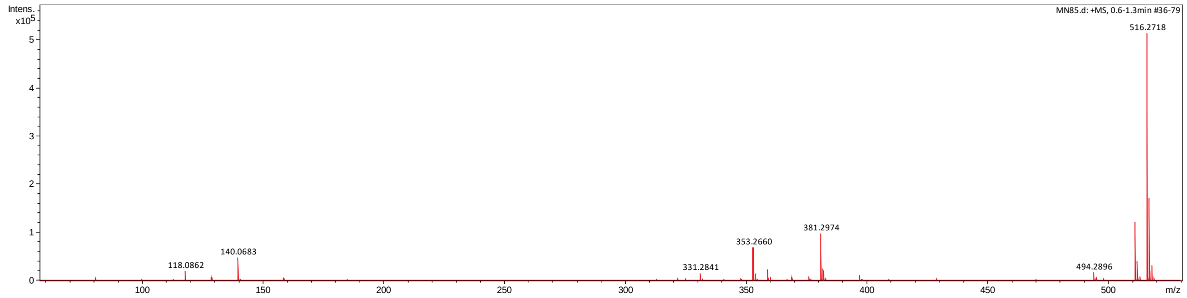

Figure S61. HRMS spectrum of 2α,3α-dihydroxy-5α-cholan-6-oxo-23,24-dinor-22-yl 4-cyanobenzoate (**22**).

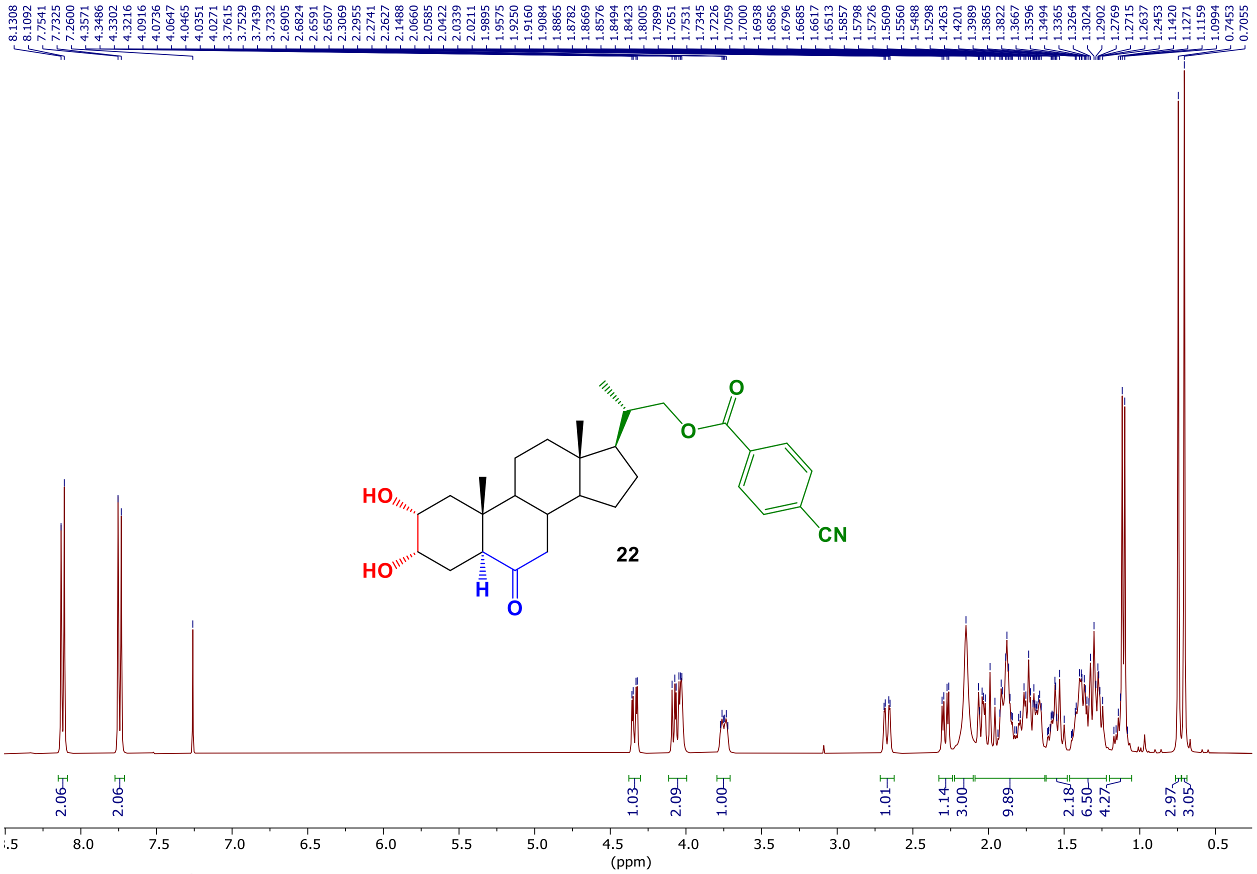

Figure S62. ^{1}H NMR spectrum of 2α,3α-dihydroxy-5α-cholan-6-oxo-23,24-dinor-22-yl 4-cyanobenzoate (**22**).

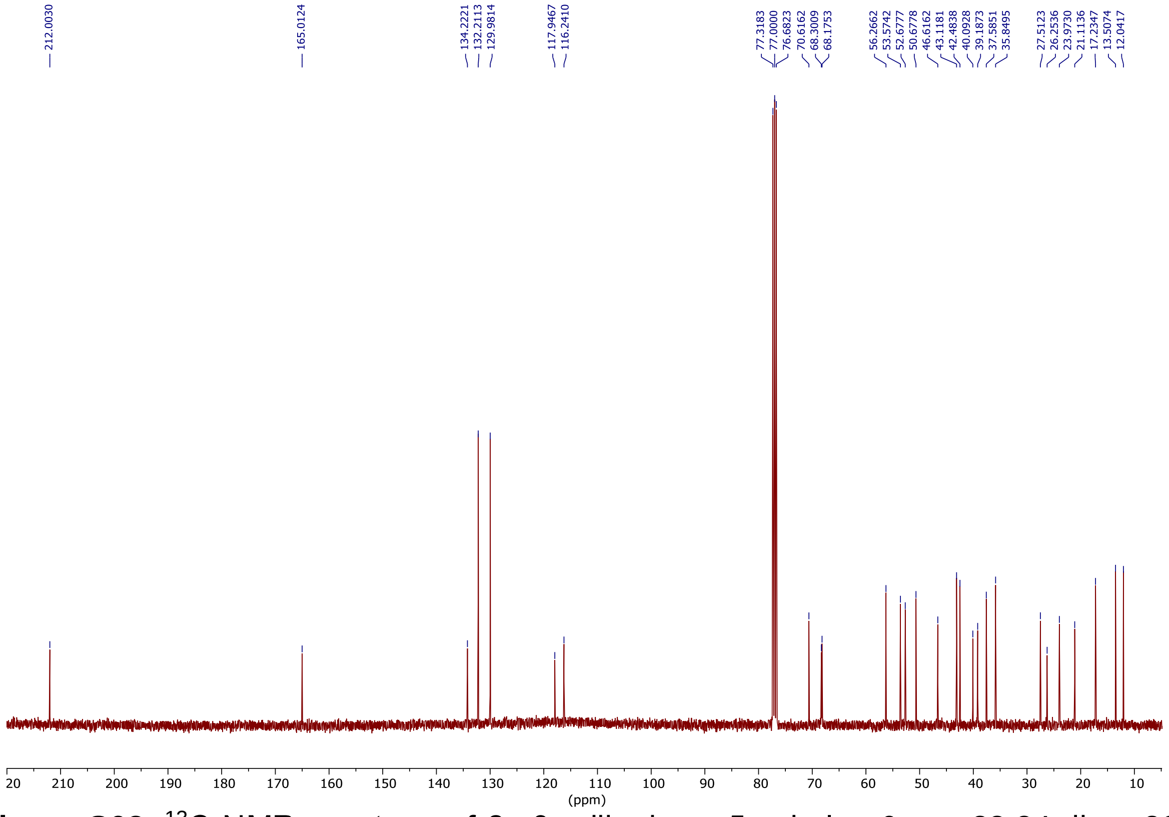

Figure S63. ^{13}C NMR spectrum of 2α,3α-dihydroxy-5α-cholan-6-oxo-23,24-dinor-22-yl 4-cyanobenzoate (**22**).

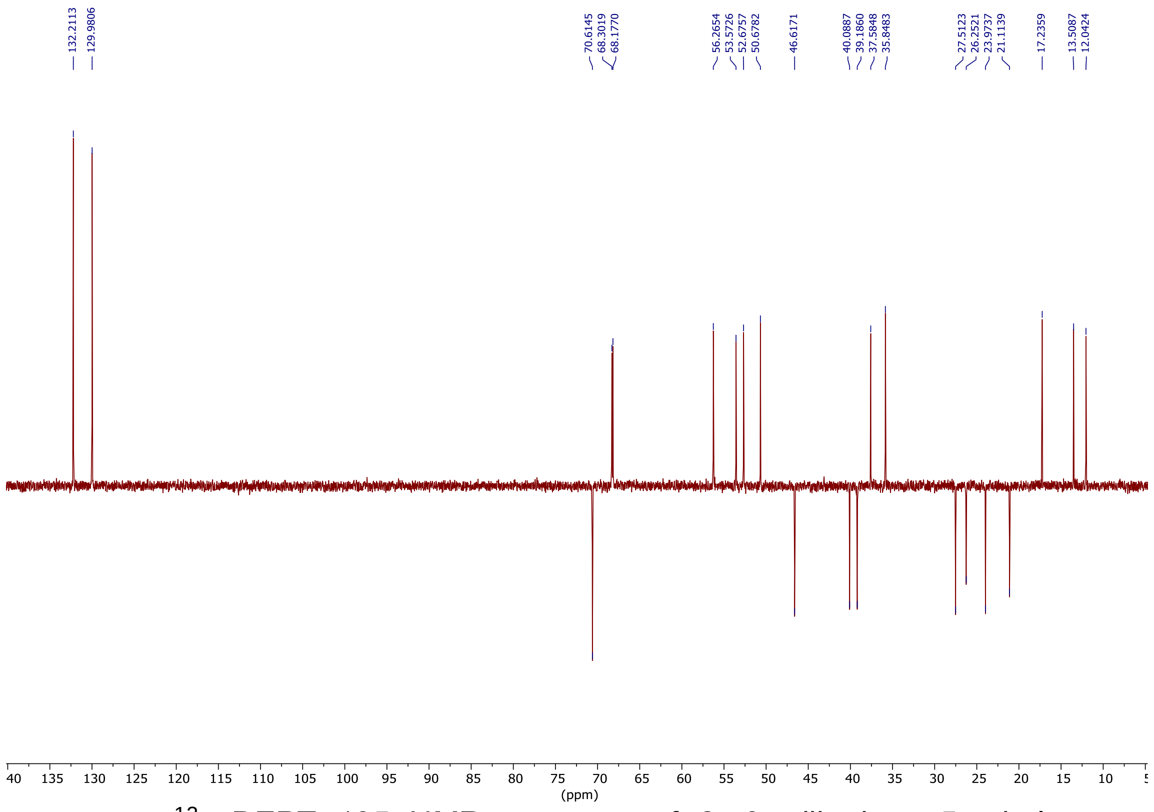

Figure S64. ^{13}C-DEPT 135 NMR spectrum of 2α,3α-dihydroxy-5α-cholan-6-oxo-23,24-dinor-22-yl 4-cyanobenzoate (**22**).

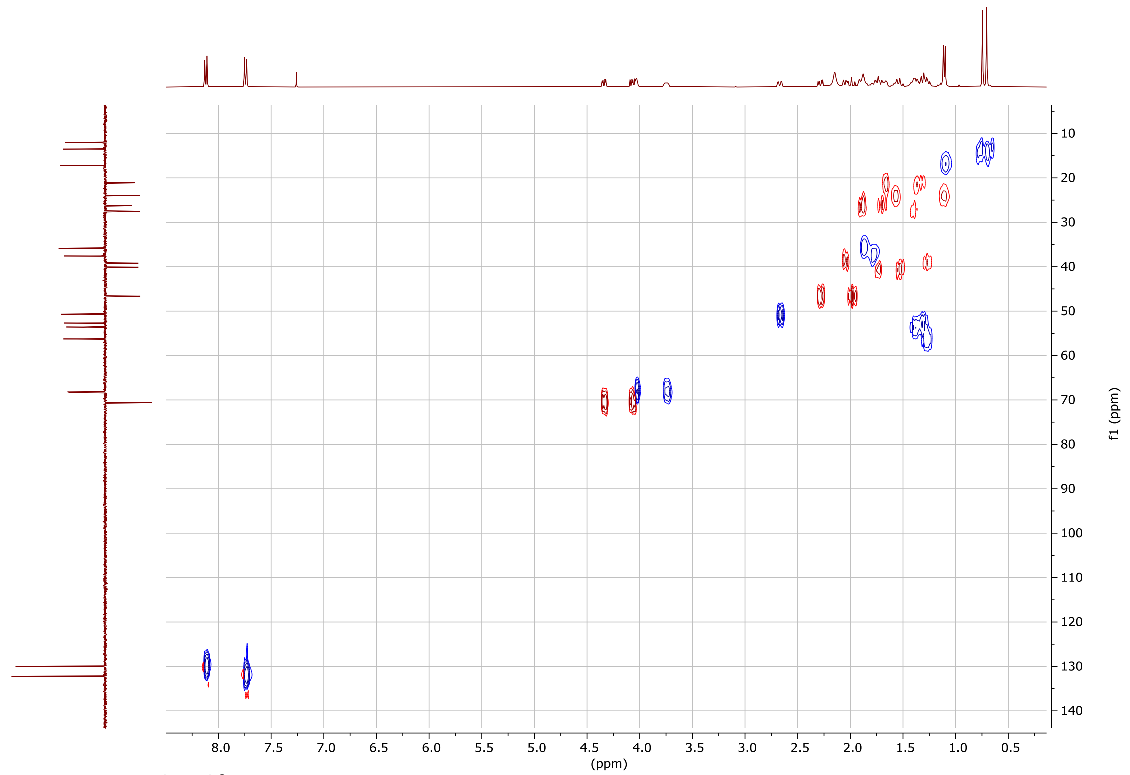

Figure S65. ^{1}H-^{13}C HSQC-ed. spectrum 2α,3α-dihydroxy-5α-cholan-6-oxo-23,24-dinor-22-yl 4-cyanobenzoate (**22**).

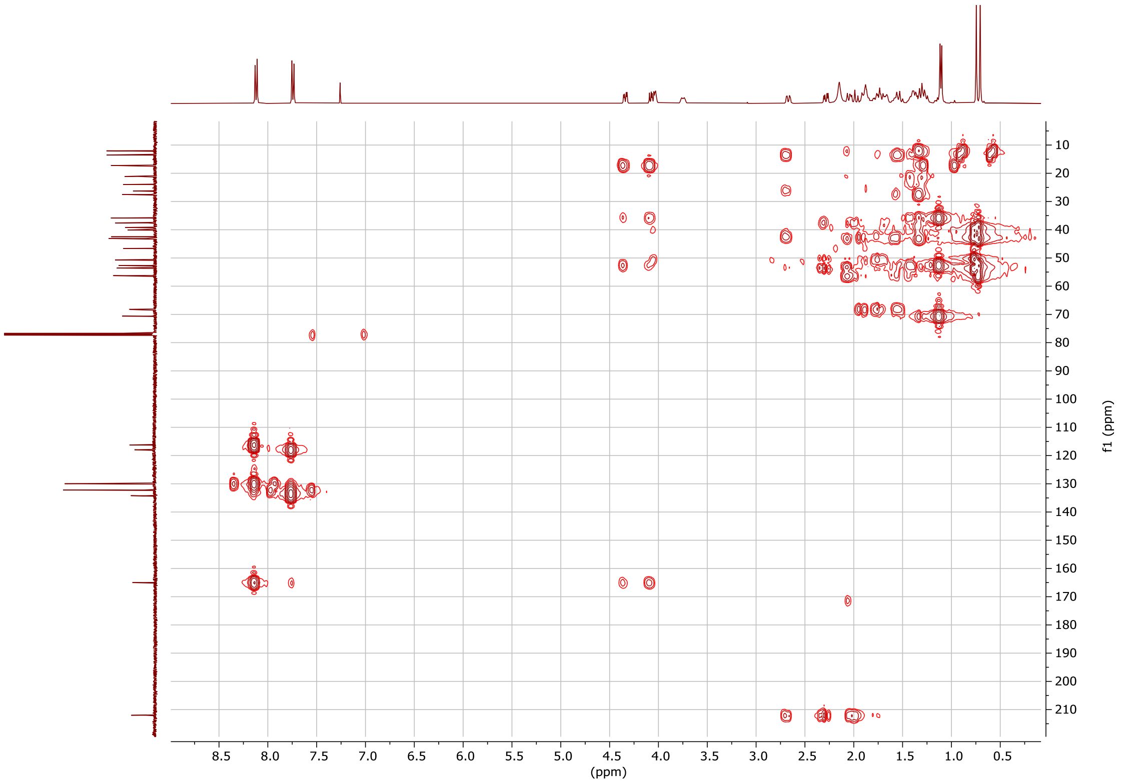

Figure S66. ^{1}H-^{13}C HMBC spectrum of 2α,3α-dihydroxy-5α-cholan-6-oxo-23,24-dinor-22-yl 4-cyanobenzoate (**22**).

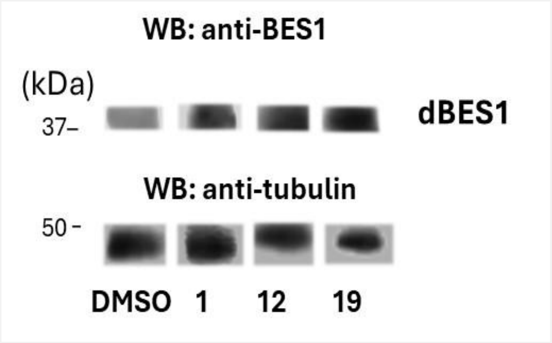

Figure S67. Representative Western blot analysis showing dephosphorylation of BES1 after treatment with active compound **12** and **19**. WB: Western blot; dBES1: dominant Brassinosteroid-Insensitive 1 Suppressor 1; DMSO: Dimethyl sulfoxide; kDa: Kilodalton. Representative images are shown. dBES1, dephosphorylated BES1.

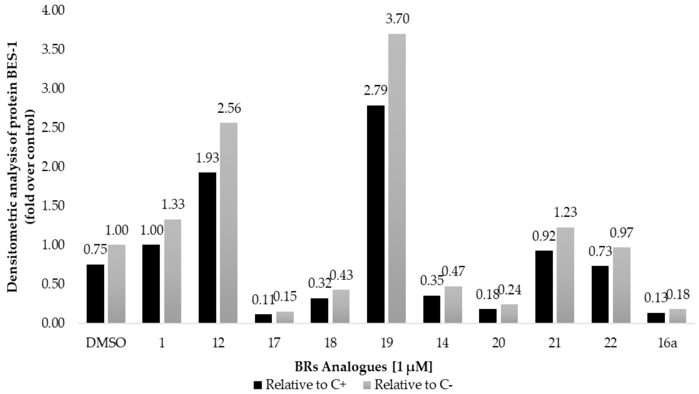

Figure S68: BES1 phosphorylation status tested by immunoblot with α-BES1 antibody in roots of *Arabidopsis thaliana* ecotype Col-0 6-day-old seedlings after analogues of BR treatment. Tubulin detected with α-tubulin antibody was used as a loading control. The graph shows the percentage of dephosphorylated BES1 relative to total BES1 detected in wild-type *Arabidopsis* ecotype (Col-0) treated with brassinolide (**1**) after 6 days.

a) Compound **12**

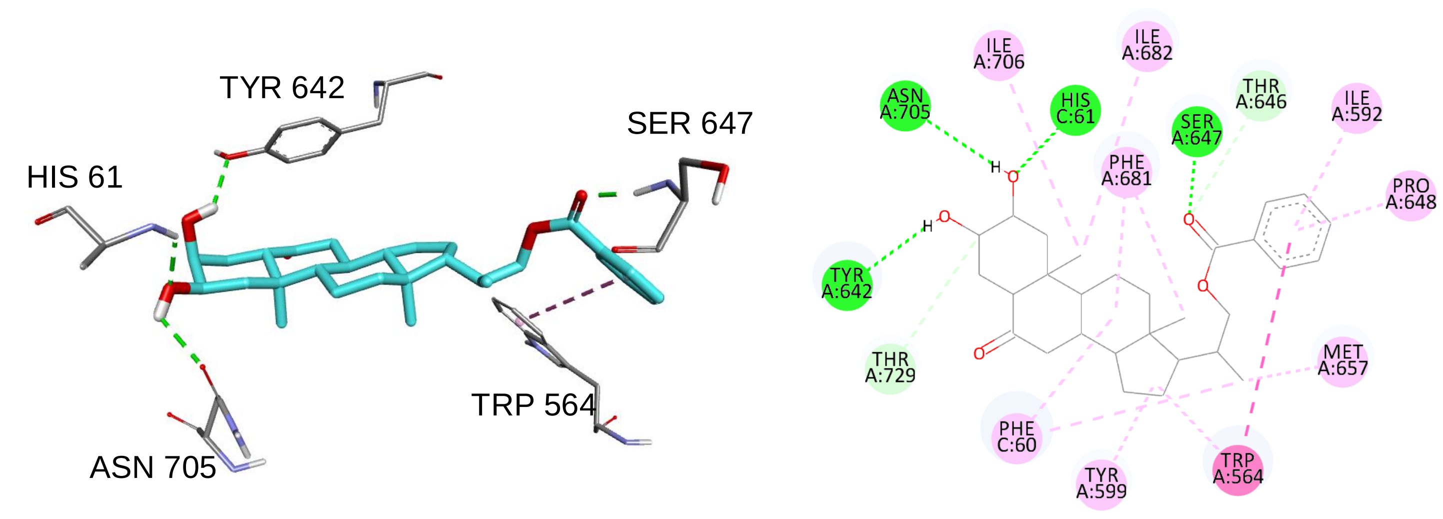

b) Compound **14**

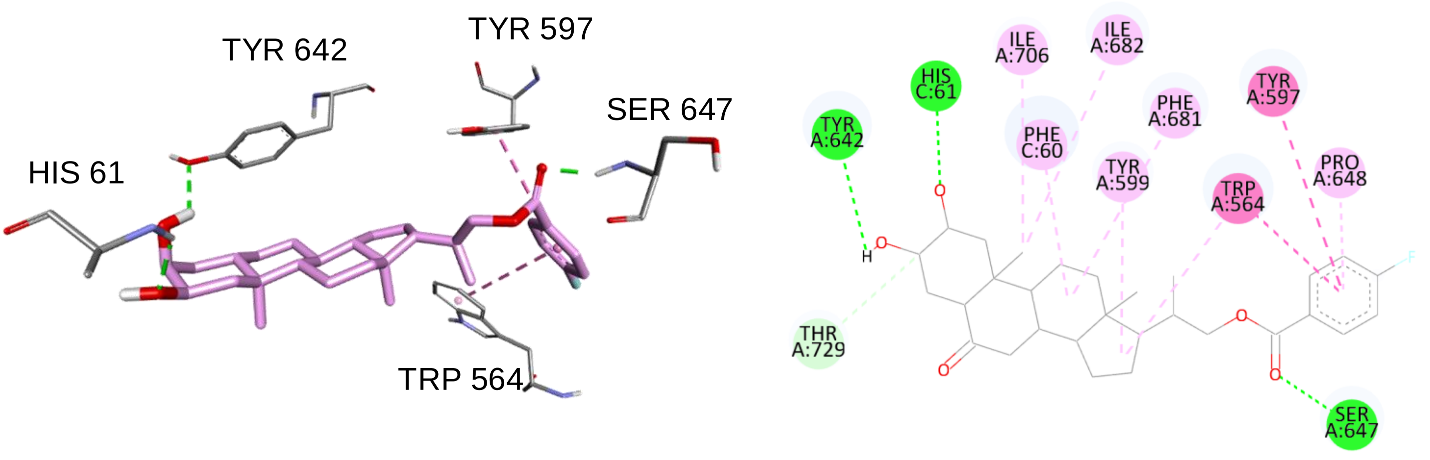

c) Compound **16a**

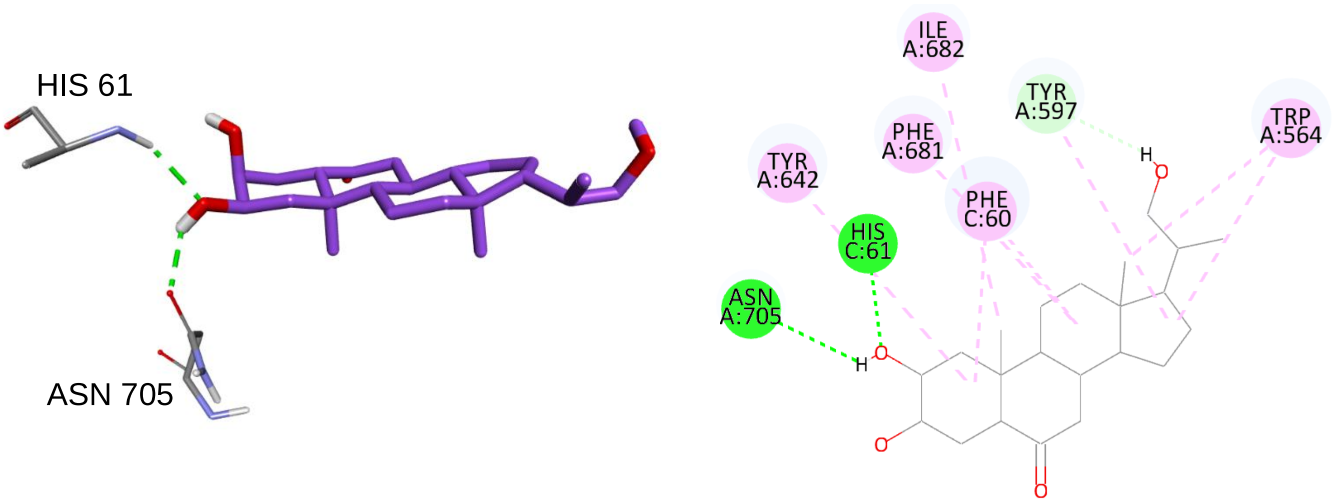

d) Compound **17**

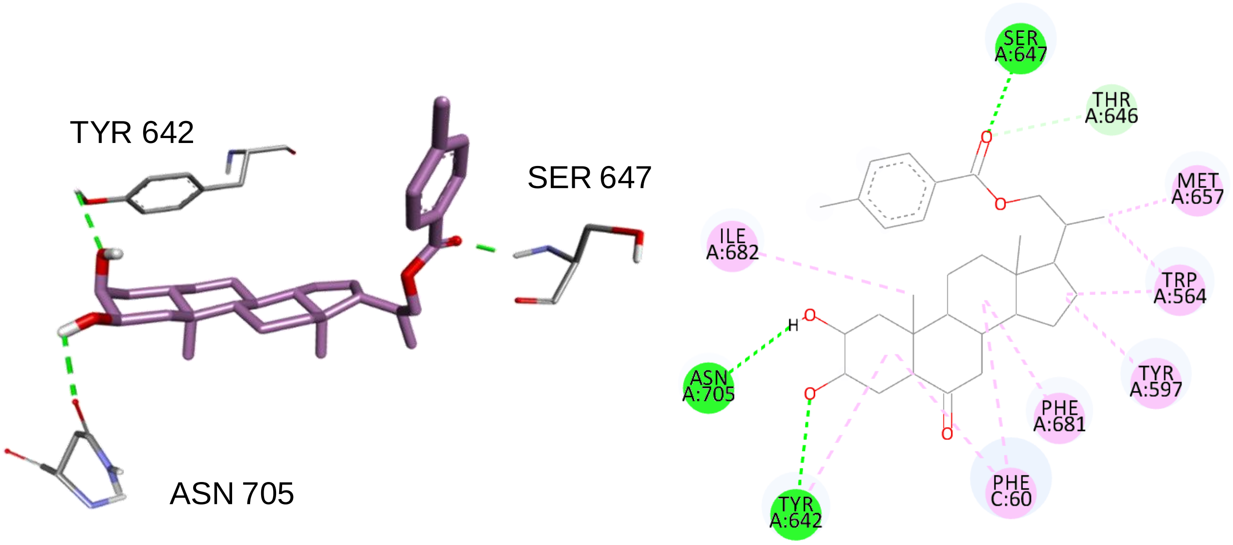

e) Compound **18**

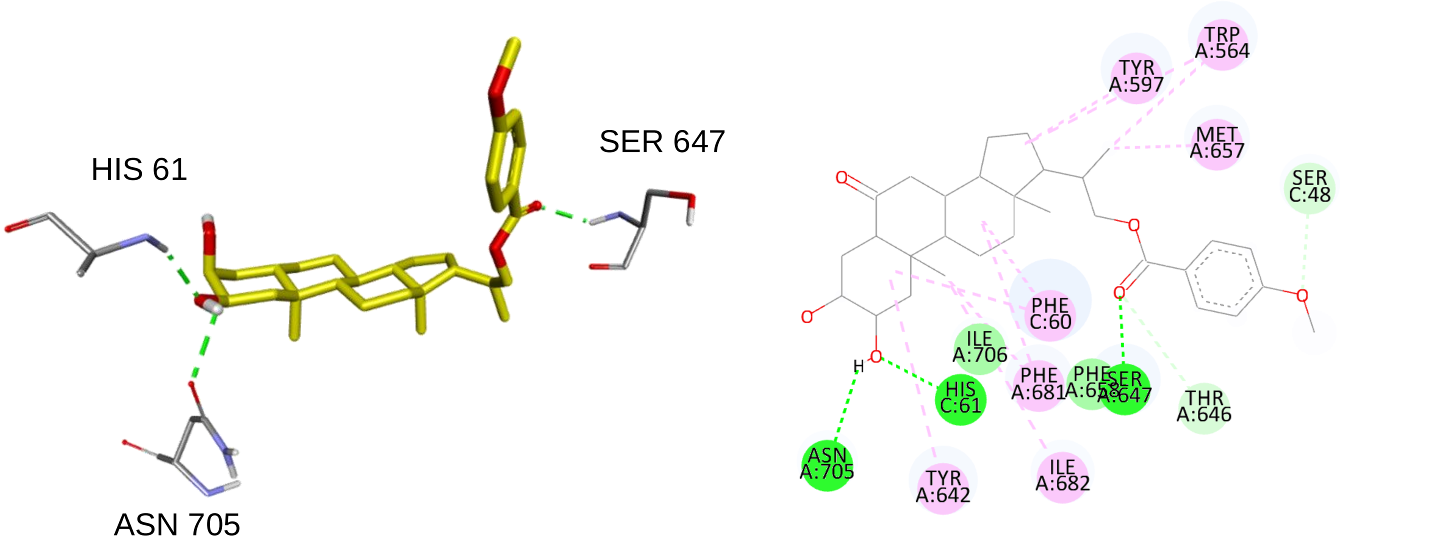

f) Compound **19**

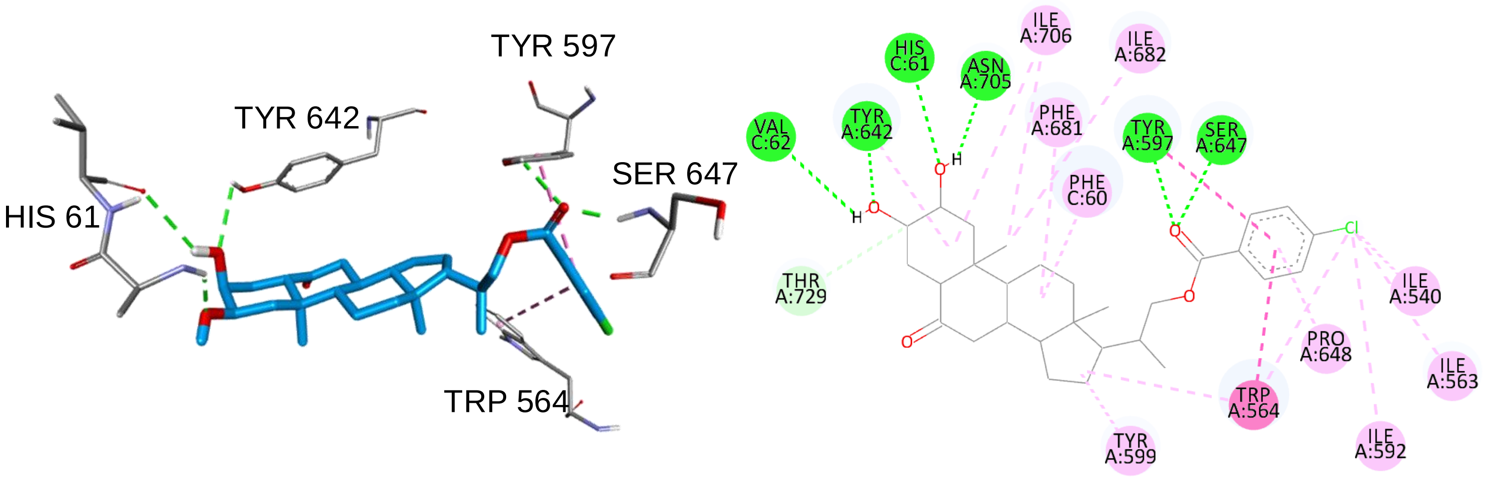

g) Compound **20**

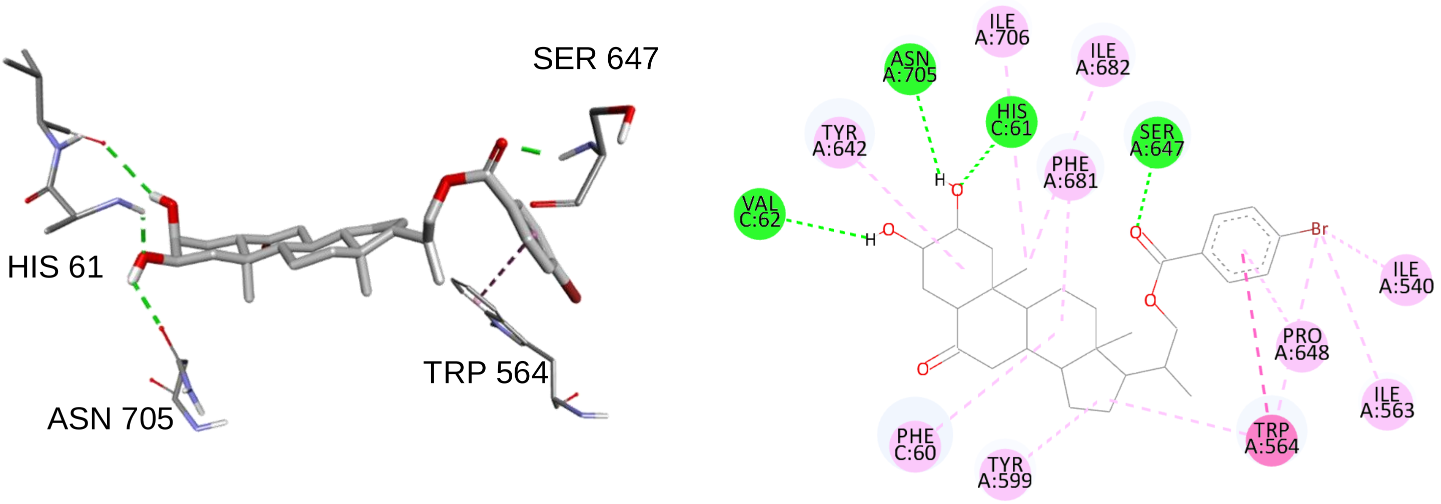

h) Compound **21**

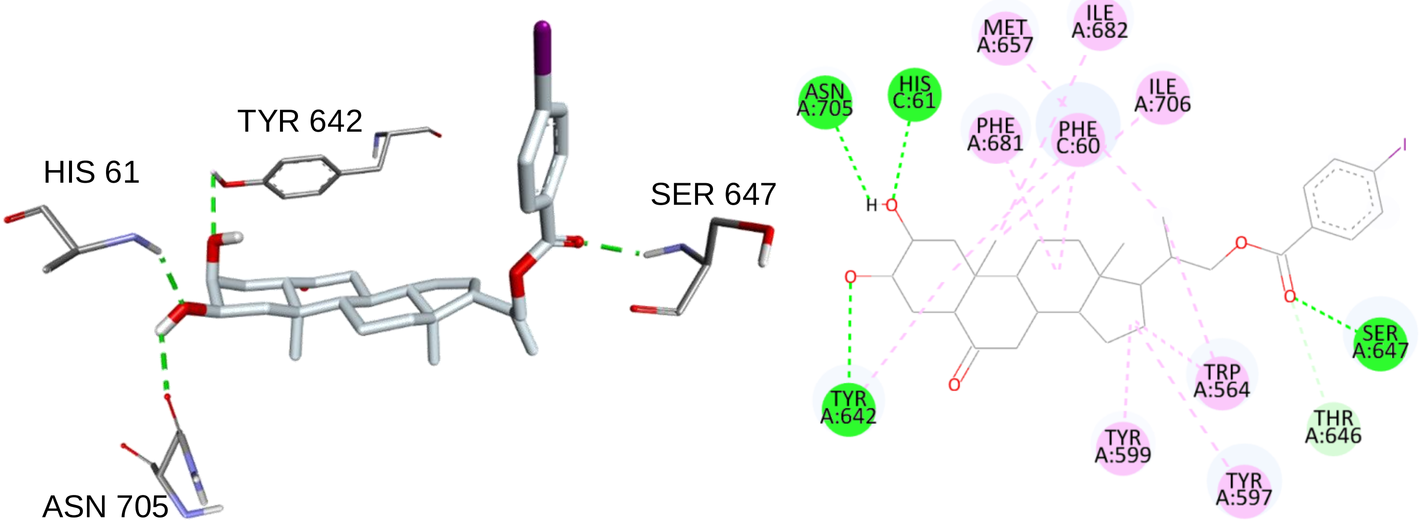

i) Compound **22**

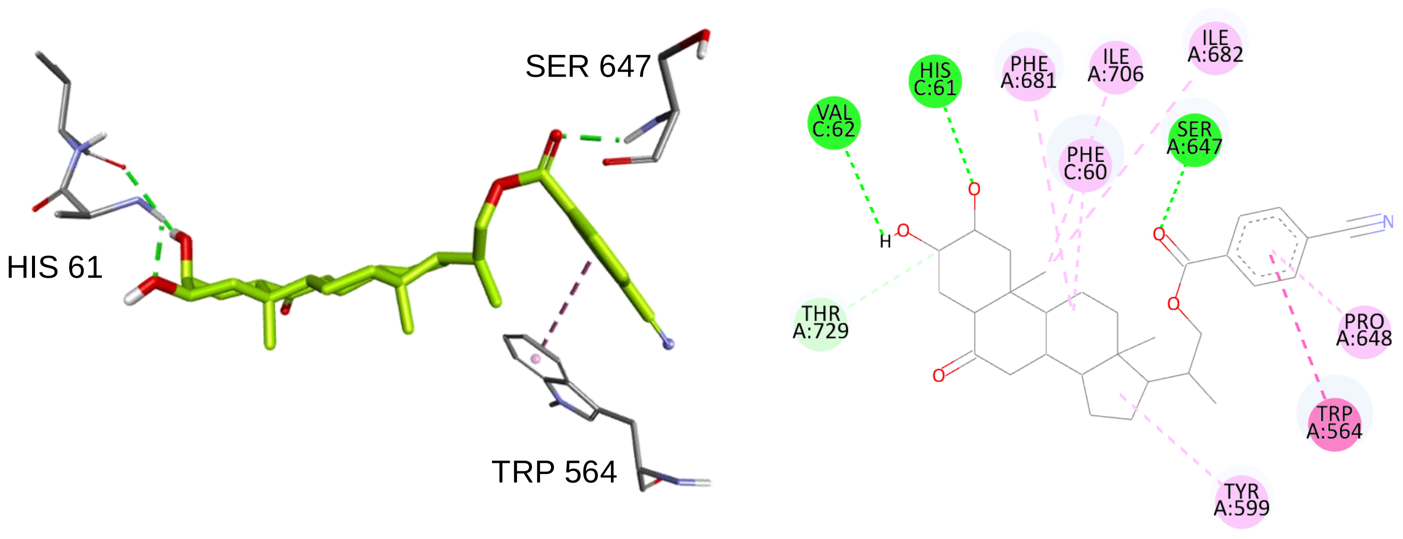

Figure S69. Protein–ligand interactions with a) compound **12** b) compound **14**; c) compound **16a**, d) compound **17**, e) compound **18**, f) compound **19**, g) compound **20**, h) compound **21** and i) compound **22**. Hydrogen bonds are represented in green segmented lines. Π–π stacking are represented in dark pink segmented lines. Hydrophobic interactions are represented in pink segmented lines. Visualization of the docked poses was performed using Discovery Studio Visualizer (BIOVIA, San Diego, CA, USA).

Table S1: Rice lamina assays using the second leaf lamina joints (angle opening, degrees) of excised leaf segments treated with BRs analogs (**1**, **12**, **14** and **17–22**) at different concentrations. Brassinolide was used as positive control at the same concentrations.

	RLIT (angle opening, degrees)		
Compounds	1×10^{-8} M	1×10^{-7} M	1×10^{-6} M
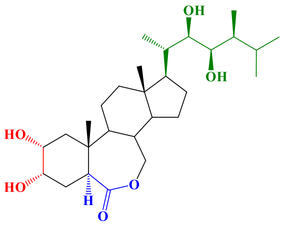 **1**	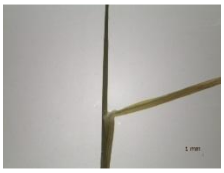	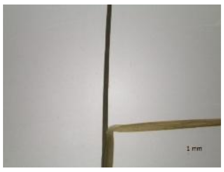	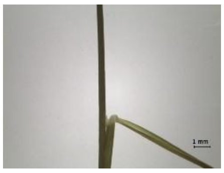
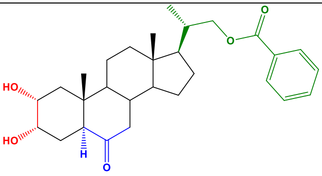 **12**	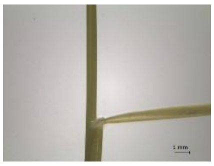	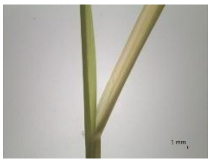	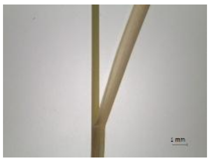
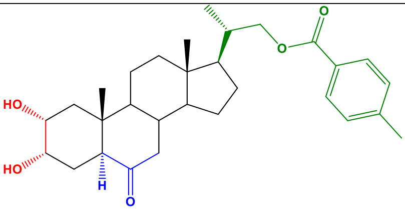 **17**	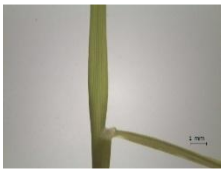	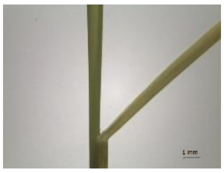	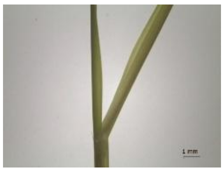
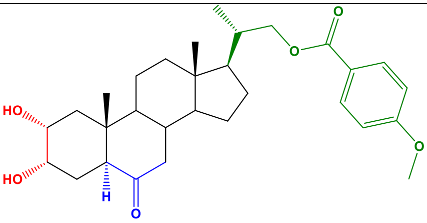 **18**	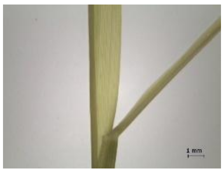	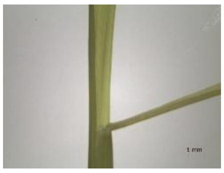	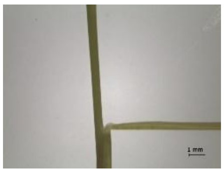
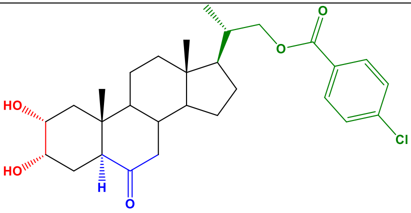 **19**	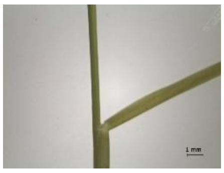	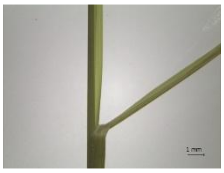	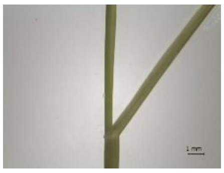
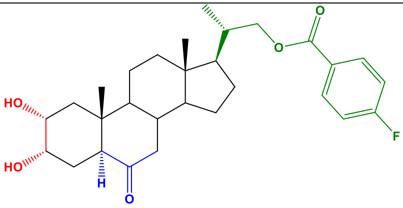 **14**	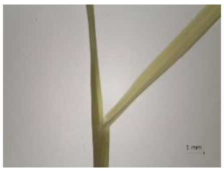	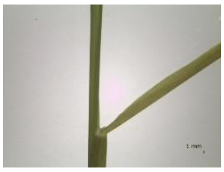	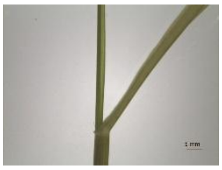

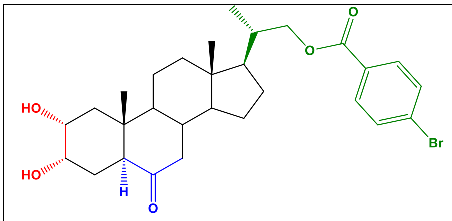 **20**	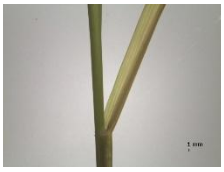	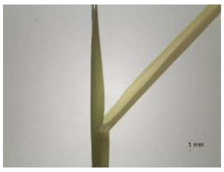	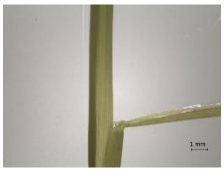
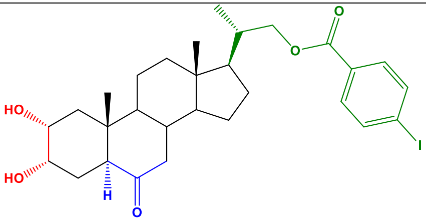 **21**	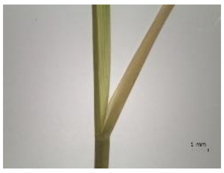	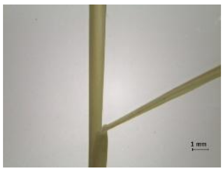	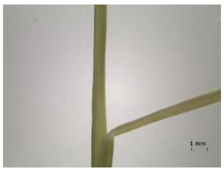
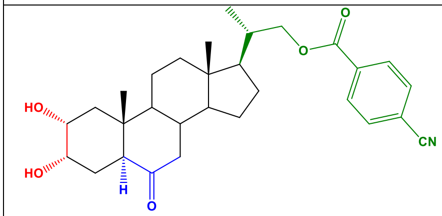 **22**	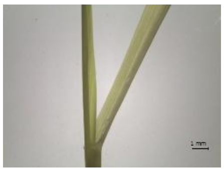	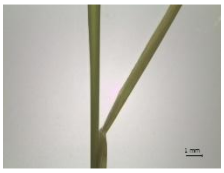	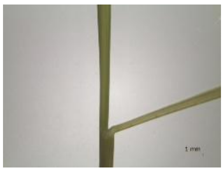

Table S2. Docked compounds–heterodimer protein contacts of synthetic analogs (**12–22**).

Compound	Protein contacts	
	Hydrogen bonds	Non-polar interactions
**12**	Asn705<br>His61<br>Tyr642<br>Ser647	Ile706, Tyr599, Phe681, Met657, Trp564, Pro648, Ile682, Ile592, Phe60
**14**	His61<br>Tyr642<br>Ser647	Ile706, Tyr597, Trp564, Phe681, Ile682, Phe60, Tyr597, Tyr599, Pro648
**16a**	Asn705<br>His61	Trp564, Phe681, Ile682, Phe60, Tyr642, Phe60, Tyr597
**17**	Asn705<br>Ser647<br>Tyr642	Tyr597, Trp564, Met657, Ile682, Phe60, Phe681, Tyr642
**18**	Ser647<br>Asn705	Tyr597, Trp564, Met657, Ile682, Phe60, Phe681, Tyr642

	His61	
**19**	Ser647<br>Val62<br>Tyr597<br>His61<br>Asn705<br>Tyr642	Tyr599, Trp564, Ile682, Phe60, Phe681, Tyr642, Ile706, Ile540, Ile563, Pro648
**20**	Ser647<br>Val62<br>His61<br>Asn705	Tyr599, Trp564, Ile682, Phe60, Phe681, Tyr642, Ile706, Ile540, Ile563, Pro648
**21**	Ser647<br>Asn705<br>Tyr642<br>His61	Tyr599, Trp564, Ile682, Phe60, Phe681, Tyr642, Ile706, Met657, Tyr597
**22**	Ser647<br>Val62<br>His61	Tyr599, Trp564, Ile682, Pro648, Phe681, Ile706, Phe60
