# Supplementary material for: Synthesis and biological evaluation of new brassinosteroid analogs with C-22 benzoate function
Source: Beilstein J Org Chem. 2026 May 18;22:753–62. doi: 10.3762/bjoc.22.57 (PMC13202480; doi:10.3762/bjoc.22.57)
Supplement: File 2 — Experimental section. [file Beilstein_J_Org_Chem-22-753-s002.pdf]

BEILSTEIN JOURNAL OF ORGANIC CHEMISTRY

Supporting Information

for

Synthesis and biological evaluation of new brassinosteroid analogs with C-22 benzoate function

María Núñez, Camila Escobar, Mario Párraga, Mauricio Soto, Luis Espinoza-Catalán, Katy Díaz and Andrés F. Olea

Beilstein J. Org. Chem. **2026,** *22,* 753–762. doi:10.3762/bjoc.22.57

Experimental section

License and Terms: This is a supporting information file under the terms of the Creative Commons Attribution License (https://creativecommons.org/licenses/by/4.0). Please note that the reuse, redistribution and reproduction in particular requires that the author(s) and source are credited and that individual graphics may be subject to special legal provisions.
The license is subject to the *Beilstein Journal of Organic Chemistry* terms and conditions: (https://www.beilstein-journals.org/bjoc/terms)

Experimental

General

All reagents were purchased from commercial suppliers and used without further purification. Melting points were measured with a SMP3 apparatus (Stuart-Scientific, now Merck KGaA, Darmstadt, Germany) and are uncorrected. ^{1}H-, ^{13}C-, ^{13}C DEPT-135, gs 2D HSQC, and gs 2D HMBC NMR spectra were recorded on an Avance Neo 400 Digital NMR spectrometer (Bruker, Rheinstetten, Germany) operating at 400.1 MHz for ^{1}H and 100.6 MHz for ^{13}C. Samples were measured as $CDCl_3$ solutions and signals are referenced to the residual peaks of $CHCl_3$ at δ = 7.26 ppm and δ = 77.00 ppm for ^{1}H and ^{13}C NMR spectra, respectively. Chemical shifts are reported in δ ppm, and coupling constants (*J*) are given in Hz; multiplicities are reported as follows: singlet (s), doublet (d), broad doublet (bd), doublet of doublets (dd), doublet of triplets (dt), triplet (t), broad triplet (bt), quartet (q), doublet of quartet (dq), doublet of double doublets (ddd), triplet of triplets (tt), and multiplet (m). IR spectra were recorded as KBr disks using an FT-IR 6700 spectrometer (Nicolet, Thermo Scientific, San Jose, CA, USA), and frequencies are reported in cm^{-1}. High-resolution mass spectra (HRMS–ESI) were recorded with a Bruker Daltonik (Bruker, Bremen, Germany). The analysis for the reaction products was performed with the following relevant parameters: drying gas temperature, 180 °C; nebulizer 0.4 bar; drying gas, 4 L/min; and spray voltage, 4.5 kV at positive mode. Accurate mass measurements were performed at a resolving power: 140,000 FWHM at range *m*/*z* 50–1300. For analytical TLC, silica gel 60 in a 0.25 mm layer was used, and TLC spots were detected by heating after spraying with 25% H_2SO_4 in H_2O.

Chromatographic separations were carried out by using a conventional column on silica gel 60 (230–400 mesh) using EtOAc-hexane gradients of increasing polarity. All organic extracts were dried over anhydrous magnesium sulfate and evaporated under reduced pressure below 40 °C.

Synthesis

5α-Cholan-6-oxo-2-ene-23,24-dinor-22-yl 4-(substituted)-benzoates (**23**–**28**).

General procedure: precursor **16** is dissolved in DCM and pyridine, and DMAP and *p*-R-PhCOCl are added with slow stirring at room temperature. The end of the reaction is verified by TLC (2 h), solvent volume is reduced to about 10 mL, and then EtOAc (30 mL) is added. The organic layer is washed with 5% $KHSO_4$ (2 × 15 mL) and water (2 × 15 mL), dried over $MgSO_4$ and filtered. The solvent is evaporated under reduced pressure. The crude is redissolved in DCM (3 mL) and chromatographed on silica gel with Hexane/EtOAc mixtures of increasing polarity (100:0 → 90:10).

5α-Cholan-6-oxo-2-ene-23,24-dinor-22-yl 4-methylbenzoate (**23**)

Compound **16** (0.30 g, 0.91 mmol); DCM (45 mL), Py (0.75 mL), DMAP (50 mg), *p*-CH_3-PhCOCl (0.36 mL, 2.72 mmol). Compound **23** is obtained as a colorless solid (0.39 g, 0.88 mmol, 96.7% yield) (m.p. = 138.1–140.1 °C). IR_{vmax} (cm^{-1}): 2943 (CH_3-); 1719 (C=O); 1610 (C=C Ar); 1374 (CH_3-); 1107 and 1038 (C-O). ^1H NMR (400.1 MHz, $CDCl_3$): δ (ppm) = 7.91 (2H, d, *J* = 8.1 Hz, H-2´); 7.21 (2H, d, *J* = 8.1 Hz, H-3´); 5.68-5.64 (1H, m, H-3); 5.56-5.52 (1H, m, H-2); 4.27 (1H, dd, *J* = 10.8 and 3.4 Hz, H-22a); 4.01 (1H, dd, *J* = 10.8 and 7.2 Hz, H-22b); 2.38 (3H, s, CH_3-Ar); 2.35-2.29 (2H, m, H-7 and H-5); 2.27-2.19 (1H, m, H-4); 1.77-1.67 (1H, m, H-8); 1.63-1.52 (2H, m, H-11 and H-15); 1.15-1.05 (1H, m, H-15); 1.10 (3H, d, *J* = 6.6 Hz, H-21);

0.70 (3H, s, H-19); 0.69 (3H, s, H-18). ^{13}C NMR (100.6 MHz, $CDCl_3$): δ (ppm) = 211.58 (C-6); 166.57 (ArCOO); 143.31 (C-4´); 129.40 (C-2´); 128.93 (C-3´); 127.65 (C-1´); 124.82 (C-3); 124.35 (C-2); 69.50 (C-22); 56.25 (C-14); 53.64 (C-5); 53.17 (C-9); 52.73 (C-17); 46.76 (C-7); 42.86 (C-13); 39.82 (C-10); 39.20 (C-1); 39.20 (C-12); 37.52 (C-8); 35.84 (C-20); 27.41 (C-16); 23.90 (C-15); 21.59 (C-4); 21.53 (CH_3-Ar); 20.96 (C-11); 17.21 (C-21); 13.36 (C-19); 11.87 (C-18).

5α-Cholan-6-oxo-2-ene-23,24-dinor-22-yl 4-methoxybenzoate (**24**)
Compound **16** (0.30 g, 0.91 mmol); DCM (45 mL), Py (0.75 mL), DMAP (50 mg), *p*-CH_3O-PhCOCl (0.37 mL, 2.73 mmol). Compound **24** is obtained as a colorless solid (0.33 g, 0.71 mmol, 78.0% yield) (m.p. = 152.2–153.8 °C). IR_{vmax} (cm^{-1}): 2965 and 3940 (CH_3-); 2904 and 2867 (CH_2-); 1709 (C=O); 1606 (C=C); 1386 (CH_3-); 1100 and 1027 (C-O). ^{1}H NMR (400.1 MHz, $CDCl_3$): δ (ppm) = 7.98 (2H, d, *J* = 9.4 Hz, H-2´); 6.90 (2H, d, *J* = 9.4 Hz, H-3´); 5.69-5.65 (1H, m, H-3); 5.57-5.53 (1H, m, H-2); 4.26 (1H, dd, *J* = 10.7 and 3.5 Hz, H-22a); 4.01 (1H, dd, *J* = 10.7 and 7.1 Hz, H-22b); 3.84 (3H, s, OCH_3); 2.36-2.31 (2H, m, H-7 and H-5); 2.28-2.20 (1H, m, H-4); 1.76-1.71 (1H, m, H-8); 1.63-1.55 (2H, m, H-11 and H-15); 1.16-1.06 (1H, m, H-15); 1.10 (3H, d, *J* = 6.6 Hz, H-21); 0.71 (3H, s, H-19); 0.70 (3H, s, H-18). ^{13}C NMR (100.6 MHz, $CDCl_3$): δ (ppm) = 211.71 (C-6); 166.36 (ArCOO); 163.20 (C-4´); 131.43 (C-2´); 124.89 (C-3); 124.41 (C-2); 122.88 (C-1´); 113.52 (C-3´); 69.46 (C-22); 56.34 (C-14); 55.34 (OCH_3); 53.73 (C-5); 53.26 (C-9); 52.81 (C-17); 46.84 (C-7); 42.92 (C-13); 39.90 (C-10); 39.27 (C-1); 39.27 (C-12); 37.60 (C-8); 35.92 (C-20); 27.47 (C-16); 23.97 (C-15); 21.64 (C-4); 21.03 (C-11); 17.28 (C-21); 13.42 (C-19); 11.93 (C-18).

5α-Cholan-6-oxo-2-ene-23,24-dinor-22-yl 4-chlorobenzoate (**25**)

Compound **16** (0.30 g, 0.91 mmol); DCM (45 mL), Py (0.75 mL), DMAP (50 mg), *p*-Cl-PhCOCl (0.70 mL, 5.46 mmol). Compound **25** is obtained as a colorless solid (0.39 g, 0.83 mmol, 91.2% yield) (m.p. = 154.5–156.0 °C). IR_{vmax} (cm^{-1}): 3020 (C=C-H); 2944 (CH_3-); 2869 (CH_2-); 1712 (C=O); 1696 (C=C); 1475 (CH_2-); 1392 (CH_3-); 1102 and 1092 (C-O). ^{1}H NMR (400.1 MHz, $CDCl_3$): δ (ppm) = 7.96 (2H, d, *J* = 8.9 Hz, H-2´); 7.41 (2H, d, *J* = 8.9 Hz, H-3´); 5.69-5.66 (1H, m, H-3); 5.57-5.54 (1H, m, H-2); 4.30 (1H, dd, *J* = 10.7 and 3.4 Hz, H-22a); 4.05 (1H, dd, *J* = 10.7 and 7.2 Hz, H-22b); 2.38-2.32 (2H, m, H-7 and H-5); 2.29-2.21 (1H, m, H-4); 1.92-1.84 (2H, m, H-16 and H-20); 1.77-1.70 (1H, m, H-8); 1.65-1.57 (2H, m, H-11 and H-15); 1.18-1.08 (1H, m, H-15); 1.11 (3H, d, *J* = 6.6 Hz, H-21); 0.72 (3H, s, H-19); 0.71 (3H, s, H-18). ^{13}C NMR (100.6 MHz, $CDCl_3$): δ (ppm) = 211.73 (C-6); 165.78 (ArCOO); 139.24 (C-4´); 130.86 (C-2´); 128.91 (C-1´); 128.67 (C-3´); 124.94 (C-3); 124.42 (C-2); 70.06 (C-22); 56.38 (C-14); 53.78 (C-5); 53.30 (C-9); 52.80 (C-17); 46.88 (C-7); 42.99 (C-13); 39.95 (C-10); 39.31 (C-1); 39.31 (C-12); 37.63 (C-8); 35.91 (C-20); 27.52 (C-16); 23.99 (C-15); 21.67 (C-4); 21.06 (C-11); 17.28 (C-21); 13.46 (C-19); 11.97 (C-18).

5α-Cholan-6-oxo-2-ene-23,24-dinor-22-yl 4-bromobenzoate (**26**)

Compound **16** (0.35 g, 1.06 mmol); DCM (50 mL), Py (0.8 mL), DMAP (50 mg), *p*-Br-PhCOCl (0.94 g, 4.28 mmol). Compound **26** is obtained as a colorless solid (0.52 g, 1.01 mmol, 95.3% yield) (m.p. = 157.8–159.1 °C). IR_{vmax} (cm^{-1}): 3020 (C=C-H); 2964 and 3937 (CH_3-); 3867 and 2849 (CH_2-); 1715 (C=O); 1702 (COO); 1591 (C=C); 1483 (CH_2-); 1397 (CH_3-); 1100 and 1071 (C-O). ^{1}H NMR (400.1 MHz, $CDCl_3$): δ (ppm) = 7.88 (2H, d, *J* = 8.6 Hz, H-2´); 7.56 (2H, d, *J* = 8.6 Hz, H-3´); 5.68-

5.64 (1H, m, H-3); 5.56-5.53 (1H, m, H-2); 4.29 (1H, dd, *J* = 10.7 and 3.5 Hz, H-22a); 4.03 (1H, dd, *J* = 10.7 and 7.2 Hz, H-22b); 2.36-2.31 (2H, m, H-7 and H-5); 2.28-2.19 (1H, m, H-4); 1.90-1.81 (2H, m, H-16 and H-20); 1.78-1.69 (1H, m, H-8); 1.63-1.53 (2H, m, H-11 and H-15); 1.16-1.06 (1H, m, H-15); 1.10 (3H, d, *J* = 6.6 Hz, H-21); 0.70 (3H, s, H-18); 0.69 (3H, s, H-19). ^{13}C NMR (100.6 MHz, $CDCl_3$): δ (ppm) = 211.63 (C-6); 165.82 (ArCOO); 131.62 (C-3´); 130.96 (C-2´); 129.31 (C-4´); 127.84 (C-1´); 124.88 (C-3); 124.38 (C-2); 70.02 (C-22); 56.31 (C-14); 53.71 (C-5); 53.23 (C-9); 52.74 (C-17); 46.81 (C-7); 42.93 (C-13); 39.88 (C-10); 39.25 (C-1); 39.25 (C-12); 37.57 (C-8); 35.85 (C-20); 27.47 (C-16); 23.94 (C-15); 21.63 (C-4); 21.01 (C-11); 17.24 (C-21); 13.41 (C-19); 11.92 (C-18).

5α-Cholan-6-oxo-2-ene-23,24-dinor-22-yl 4-iodobenzoate (**27**)

Compound **16** (0.30 g, 0.91 mmol); DCM (45 mL), Py (0.75 mL), DMAP (50 mg), *p*-I-PhCOCl (1.00 g, 3.75 mmol). Compound **27** is obtained as a colorless solid (0.49 g, 0.87 mmol, 95.6% yield) (m.p. = 169.7–170.2 °C). IR_{vmax} (cm^{-1}): 3026 (C=C-H); 2941 (CH_3-); 2904 and 2865 (CH_2-); 1710 (C=O); 1582 (C=C); 1471 (CH_2-); 1391 (CH_3-); 1100 and 1056 (C-O). ^{1}H NMR (400.1 MHz, $CDCl_3$): δ (ppm) = 7.79 (2H, d, *J* = 8.4 Hz, H-3´); 7.72 (2H, d, *J* = 8.4 Hz, H-2´); 5.69-5.65 (1H, m, H-3); 5.56-5.54 (1H, m, H-2); 4.29 (1H, dd, *J* = 10.7 and 3.3 Hz, H-22a); 4.03 (1H, dd, *J* = 10.7 and 7.2 Hz, H-22b); 2.37-2.31 (2H, m, H-7 and H-5); 2.28-2.20 (1H, m, H-4); 1.90-1.83 (2H, m, H-16 and H-20); 1.79-1.70 (1H, m, H-8); 1.63-1.54 (2H, m, H-11 and H-15); 1.17-1.06 (1H, m, H-15); 1.10 (3H, d, *J* = 6.5 Hz, H-21); 0.71 (3H, s, H-19); 0.70 (3H, s, H-18). ^{13}C NMR (100.6 MHz, $CDCl_3$): δ (ppm) = 211.73 (C-6); 166.09 (ArCOO); 137.64 (C-3´); 130.90 (C-2´); 129.88 (C-4´); 124.89 (C-3); 124.40 (C-2); 100.56 (C-1´); 70.03 (C-22); 56.32 (C-14); 53.73 (C-5); 53.23 (C-9); 52.74 (C-17); 46.84 (C-7);

42.94 (C-13); 39.91 (C-10); 39.26 (C-1); 39.26 (C-12); 37.58 (C-8); 35.86 (C-20); 27.48 (C-16); 23.96 (C-15); 21.64 (C-4); 21.02 (C-11); 17.25 (C-21); 13.44 (C-19); 11.94 (C-18).

5α-Cholan-6-oxo-2-ene-23,24-dinor-22-yl 4-cyanobenzoate (**28**)

Compound **16** (0.36 g, 1.09 mmol); DCM (50 mL), Py (0.8 mL), DMAP (50 mg), *p*-CN-PhCOCl (0.69 g, 4.17 mmol). Compound **28** is obtained as a colorless solid (0.47 g, 1.02 mmol, 93.6% yield) (m.p. = 183.3–185.0 °C). IR_{vmax} (cm^{-1}): 3023 (C=C-H); 2965 and 2941 (CH_3-); 2904 and 2848 (CH_2-); 2229 (CN); 1720 (C=O); 1707 (COO); 1458 (CH_2-); 1391 (CH_3-); 1114 and 1020 (C-O). ^1H NMR (400.1 MHz, $CDCl_3$): δ (ppm) = 8.11 (2H, d, *J* = 8.4 Hz, H-2´); 7.73 (2H, d, *J* = 8.4 Hz, H-3´); 5.68-5.64 (1H, m, H-3); 5.56-5.53 (1H, m, H-2); 4.33 (1H, dd, *J* = 10.7 and 3.4 Hz, H-22a); 4.07 (1H, dd, *J* = 10.7 and 7.2 Hz, H-22b); 2.36-2.31 (2H, m, H-7 and H-5); 2.27-2.19 (1H, m, H-4); 1.93-1.83 (2H, m, H-16 and H-20); 1.78-1.690 (1H, m, H-8); 1.64-1.54 (2H, m, H-11 and H-15); 1.17-1.07 (1H, m, H-15); 1.10 (3H, d, *J* = 6.5 Hz, H-21); 0.71 (3H, s, H-19); 0.69 (3H, s, H-18). ^{13}C NMR (100.6 MHz, $CDCl_3$): δ (ppm) = 211.64 (C-6); 164.91 (ArCOO); 134.19 (CN); 132.15 (C-3´); 129.93 (C-2´); 124.86 (C-3); 124.36 (C-2); 117.90 (C-4´); 116.20 (C-1´); 70.58 (C-22); 56.29 (C-14); 53.70 (C-5); 53.20 (C-9); 52.67 (C-17); 46.80 (C-7); 42.94 (C-13); 39.88 (C-10); 39.23 (C-1); 39.23 (C-12); 37.55 (C-8); 35.82 (C-20); 27.47 (C-16); 23.93 (C-15); 21.61 (C-4); 20.99 (C-11); 17.22 (C-21); 13.41 (C-19); 11.92 (C-18).

2α,3α-Dihydroxy-5α-cholan-6-oxo-23,24-dinor-22-yl 4-substituted-benzoates (**17-22**).

General procedure for Sharpless dihydroxylation [1]: In a mixture of *tert*-butanol and water 1:1 (v/v) are dissolved the correxsponding olefines, hydroquinidine 4-chloro-

benzoate (DHQD-CLB), methanesulfonamide ($CH_3SO_2NH_2$), potassium carbonate (K_2CO_3), and potassium ferricyanide ($K_3[Fe(CN)_6]$). Then, osmium tetroxide (OsO_4) in *tert*-butanol (1 g per 20 mL) is added. The reaction mixture is stirred at room temperature for 36 h. A saturated solution of sodium sulfite (Na_2SO_3) is then added. After additional 30 min of stirring, the reaction mixture is diluted with ethyl acetate (30 mL) and extracted with water (2 × 20 mL). The combined organic fractions are dried over anhydrous magnesium sulfate and evaporated under reduced pressure. Column chromatography on silica gel with hexane/EtOAc/MeOH mixtures of increasing polarity (6:4:0 → 4.8:4.8:0.4) leads to the desired product.

2α,3α-Dihydroxy-5α-cholan-6-oxo-23,24-dinor-22-yl 4-methylbenzoate(**17**)

Olefin **23** (0.2 g, 0.45 mmol); DHQD-CLB (0.06 g, 0.13 mmol); $CH_3SO_2NH_2$ (0.10 g, 1.05 mmol); K_2CO_3 (0.35 g, 2.53 mmol); $K_3[Fe(CN)_6]$ (0.86 g, 2.61 mmol); OsO_4 (0.25 mL, 0.05 mmol). Compound **17** is obtained as a colorless solid (0.17 g, 0.35 mmol, 77.8% yield) (m.p. = 185.1–187.0 °C). IR_{vmax} (cm^{-1}): 3398 (O-H); 2944 (CH_3-); 2866 and 2848 (CH_2-); 1716 (C=O); 1611 (C=C); 1452 (CH_2-); 1389 (CH_3-); 1108 and 1041 (C-O). ^1H NMR (400.1 MHz, $CDCl_3$): δ (ppm) = 7.89 (2H, d, *J* = 8.1 Hz, H-2´); 7.21 (2H, d, *J* = 8.1 Hz, H-3´); 4.26 (1H, dd, *J* = 10.4 and 3.4 Hz, H-22a); 4.02-3.97 (2H, m, H-2 and H-22b); 3.71 (1H, bd, *J* = 10.2 Hz, H-3); 2.98 (2H, bs, 2 x OH); 2.65 (1H, bd, *J* = 10.6 Hz, H-5); 2.38 (3H, s, CH_3-Ar); 2.25 (1H, dd, *J* = 13.1 and 4.4 Hz, H-7a); 1.09 (3H, d, *J* = 6.7 Hz, H-21); 0.72 (3H, s, H-19); 0.68 (3H, s, H-18). ^{13}C NMR (100.6 MHz, $CDCl_3$): δ (ppm) = 211.97 (C-6); 166.83 (ArCOO); 143.50 (C-4´); 129.53 (C-2´); 129.07 (C-3´); 127.74 (C-1´); 69.65 (C-22); 68.38 (C-3); 68.27 (C-2); 56.33 (C-14); 53.66 (C-9); 52.80 (C-17); 50.70 (C-5); 46.69 (C-7); 43.12 (C-13); 42.54 (C-10); 40.18 (C-1); 39.24 (C-12); 37.65 (C-8); 35.95 (C-20); 27.53 (C-16);

26.28 (C-4); 24.03 (C-15); 21.64 (Ar-CH_3); 21.17 (C-11); 17.31 (C-21); 13.54 (C-19); 12.08 (C-18). HRMS-ESI (positive mode): m/z calculated for $C_{30}H_{42}O_5$: 482.3032 $[M]^+$; found 483.3116 $[M + H]^+$.

2α,3α-Dihydroxy-5α-cholan-6-oxo-23,24-dinor-22-yl 4-methoxybenzoate (**18**)
Olefin **24** (0.3 g, 0.65 mmol); DHQD-CLB (0.08 g, 0.17 mmol); $CH_3SO_2NH_2$ (0.14 g, 1.47 mmol); K_2CO_3 (0.51 g, 3.69 mmol); $K_3[Fe(CN)_6]$ (1.24 g, 3.77 mmol); OsO_4 (0.35 mL, 0.07 mmol). Compound **18** is obtained as a colorless solid (0.22 g, 0.44 mmol, 67.9% yield) (m.p. = 170.7–172.2 °C). IR_{vmax} (cm^{-1}): 3395 (O-H); 2943 (CH_3-); 2867 and 2848 (CH_2-); 1711 (C=O); 1607 (C=C); 1463 (CH_2-); 1389 (CH_3-); 1103 and 1038 (C-O). ^{1}H NMR (400.1 MHz, $CDCl_3$): δ (ppm) = 7.98 (2H, d, *J* = 9.4 Hz, H-2´); 6.92 (2H, d, *J* = 9.4 Hz, H-3´); 4.27 (1H, dd, *J* = 10.7 and 3.4 Hz, H-22a); 4.03-3.99 (2H, m, H-2 and H-22b); 3.85 (3H, s, OCH_3); 3.75 (1H, bd, *J* = 10.5 Hz, H-3); 2.68 (1H, dd, *J* = 12.5 and 2.4 Hz, H-5); 2.29 (1H, dd, *J* = 13.1 and 4.5 Hz, H-7a); 2.16 (2H, bs, 2 x OH); 1.15-1.06 (1H, m, H-15); 1.11 (3H, d, *J* = 6.6 Hz, H-21); 0.75 (3H, s, H-19); 0.70 (3H, s, H-18). ^{13}C NMR (100.6 MHz, $CDCl_3$): δ (ppm) = 212.06 (C-6); 166.52 (ArCOO); 163.26 (C-4´); 131.48 (C-2´); 122.86 (C-1´); 113.58 (C-3´); 69.52 (C-22); 68.34 (C-3); 68.22 (C-2); 56.30 (C-14); 55.40 (OCH_3); 53.63 (C-9); 52.80 (C-17); 50.69 (C-5); 46.67 (C-7); 43.10 (C-13); 42.51 (C-10); 40.13 (C-1); 39.22 (C-12); 37.63 (C-8); 35.93 (C-20); 27.51 (C-16); 26.28 (C-4); 24.01 (C-15); 21.15 (C-11); 17.30 (C-21); 13.52 (C-19); 12.06 (C-18). HRMS-ESI (positive mode): m/z calculated for $C_{30}H_{42}O_6$: 498.2981 $[M]^+$; found 499.3051 $[M + H]^+$.

2α,3α-Dihydroxy-5α-cholan-6-oxo-23,24-dinor-22-yl 4-chlorobenzoate (**19**)
Olefin **25** (0.37 g, 0.79 mmol); DHQD-CLB (0.10 g, 0.22 mmol); $CH_3SO_2NH_2$ (0.17 g, 1.79 mmol); K_2CO_3 (0.63 g, 4.56 mmol); $K_3[Fe(CN)_6]$ (1.53 g, 4.65 mmol); OsO_4

(0.43 mL, 0.08 mmol). Compound **19** is obtained as a colorless solid (0.26 g, 0.52 mmol, 65.8% yield) (m.p. = 140.7–144.4 °C). IR_{vmax} (cm^{-1}): 3447 (O-H); 2940 (CH_3-); 2869 (CH_2-); 1722 (C=O); 1697 (COO); 1596 (C=C); 1488 (CH_2-); 1399 (CH_3-); 1104 and 1047 (C-O). ^{1}H NMR (400.1 MHz, $CDCl_3$): δ (ppm) = 7.96 (2H, d, *J* = 8.9 Hz, H-2´); 7.41 (2H, d, *J* = 8.9 Hz, H-3´); 4.30 (1H, dd, *J* = 10.7 and 3.4 Hz, H-22a); 4.06-4.01 (2H, m, H-2 and H-22b); 3.76 (1H, dt, *J* = 10.7 and 3.1 Hz, H-3); 2.68 (1H, dd, *J* = 12.6 and 2.7 Hz, H-5); 2.28 (1H, dd, *J* = 13.1 and 4.5 Hz, H-7a); 2.27 (2H, bs, 2 x OH); 1.17-1.06 (1H, m, H-15); 1.11 (3H, d, *J* = 6.6 Hz, H-21); 0.75 (3H, s, H-19); 0.71 (3H, s, H-18). ^{13}C NMR (100.6 MHz, $CDCl_3$): δ (ppm) = 211.95 (C-6); 165.87 (ArCOO); 139.30 (C-4´); 130.89 (C-2´); 128.89 (C-1´); 128.71 (C-3´); 70.08 (C-22); 68.36 (C-3); 68.26 (C-2); 56.32 (C-14); 53.64 (C-9); 52.77 (C-17); 50.70 (C-5); 46.67 (C-7); 43.14 (C-13); 42.53 (C-10); 40.16 (C-1); 39.23 (C-12); 37.64 (C-8); 35.92 (C-20); 27.54 (C-16); 26.28 (C-4); 24.02 (C-15); 21.16 (C-11); 17.29 (C-21); 13.54 (C-19); 12.08 (C-18). HRMS-ESI (positive mode): m/z calculated for $C_{29}H_{39}O_5Cl$: 502.2486 $[M]^+$; found 503.2550 $[M + H]^+$.

2α,3α-Dihydroxy-5α-cholan-6-oxo-23,24-dinor-22-yl 4-bromobenzoate(**20**)

Olefin **26** (0.51 g, 0.99 mmol); DHQD-CLB (0.13 g, 0.28 mmol); $CH_3SO_2NH_2$ (0.22 g, 2.31 mmol); K_2CO_3 (0.80 g, 5.79 mmol); $K_3[Fe(CN)_6]$ (1.96 g, 5.95 mmol); OsO_4 (0.57 mL, 0.11 mmol). Compound **20** is obtained as a colorless solid (0.46 g, 0.84 mmol, 84.9% yield) (m.p. = 209.7–211.4 °C). IR_{vmax} (cm^{-1}): 3488 (O-H); 2970 and 2942 (CH_3-); 2869 (CH_2-); 1719 (C=O); 1697 (COO); 1591 (C=C); 1483 (CH_2-); 1385 (CH_3-); 1102 and 1047 (C-O). ^{1}H NMR (400.1 MHz, $CDCl_3$): δ (ppm) = 7.89 (2H, d, *J* = 8.4 Hz, H-2´); 7.58 (2H, d, *J* = 8.4 Hz, H-3´); 4.30 (1H, dd, *J* = 10.7 and 3.4 Hz, H-22a); 4.06-4.01 (2H, m, H-2 and H-22b); 3.76 (1H, dt, *J* = 11.2 and 3.9 Hz, H-3);

2.68 (1H, dd, *J* = 12.6 and 2.8 Hz, H-5); 2.29 (1H, dd, *J* = 13.1 and 4.5 Hz, H-7a); 1.17-1.06 (1H, m, H-15); 1.10 (3H, d, *J* = 6.5 Hz, H-21); 0.75 (3H, s, H-19); 0.71 (3H, s, H-18). ^{13}C NMR (100.6 MHz, $CDCl_3$): δ (ppm) = 212.01 (C-6); 165.99 (ArCOO); 131.70 (C-3´); 131.02 (C-2´); 129.32 (C-4´); 127.95 (C-1´); 70.08 (C-22); 68.33 (C-3); 68.22 (C-2); 56.29 (C-14); 53.61 (C-9); 52.74 (C-17); 50.69 (C-5); 46.65 (C-7); 43.12 (C-13); 42.51 (C-10); 40.13 (C-1); 39.21 (C-12); 37.62 (C-8); 35.89 (C-20); 27.52 (C-16); 26.27 (C-4); 24.00 (C-15); 21.14 (C-11); 17.27 (C-21); 13.52 (C-19); 12.06 (C-18). HRMS-ESI (positive mode): m/z calculated for $C_{29}H_{39}O_5Br$: 546.1981 $[M]^+$; found 571.1857 $[M + Na]^+$.

2α,3α-Dihydroxy-5α-cholan-6-oxo-23,24-dinor-22-yl 4-iodobenzoate (**21**)
Olefin **27** (0.5 g, 0.89 mmol); DHQD-CLB (0.12 g, 0.26 mmol); $CH_3SO_2NH_2$ (0.21 g, 2.21 mmol); K_2CO_3 (0.76 g, 5.50 mmol); $K_3[Fe(CN)_6]$ (1.86 g, 5.65 mmol); OsO_4 (0.55 mL, 0.11 mmol). Compound **21** is obtained as a colorless solid (0.41 g, 0.69 mmol, 77.5% yield) (m.p. = 201.7–202.9 °C). IR_{vmax} (cm^{-1}): 3472 (O-H); 2977 and 2949 (CH_3-); 2902 and 2864 (CH_2-); 1719 (C=O); 1703 (COO); 1584 (C=C); 1470 (CH_2-); 1393 (CH_3-); 1106 and 1055 (C-O). ^{1}H NMR (400.1 MHz, $CDCl_3$): δ (ppm) = 7.80 (2H, d, *J* = 8.5 Hz, H-3´); 7.73 (2H, d, *J* = 8.5 Hz, H-2´); 4.29 (1H, dd, *J* = 10.7 and 3.4 Hz, H-22a); 4.05-4.01 (2H, m, H-2 and H-22b); 3.76 (1H, bd, *J* = 8.9 Hz, H-3); 2.68 (1H, bd, *J* = 11.1 Hz, H-5); 2.29 (1H, dd, *J* = 13.1 and 4.4 Hz, H-7a); 1.17-1.06 (1H, m, H-15); 1.10 (3H, d, *J* = 6.5 Hz, H-21); 0.75 (3H, s, H-19); 0.70 (3H, s, H-18). ^{13}C NMR (100.6 MHz, $CDCl_3$): δ (ppm) = 211.97 (C-6); 166.21 (ArCOO); 137.71 (C-3´); 130.94 (C-2´); 129.89 (C-4´); 100.61 (C-1´); 70.07 (C-22); 68.35 (C-3); 68.23 (C-2); 56.29 (C-14); 53.61 (C-9); 52.75 (C-17); 50.69 (C-5); 46.66 (C-7); 43.12 (C-13); 42.51 (C-10); 40.15 (C-1); 39.21 (C-12); 37.62 (C-8); 35.89 (C-20); 27.52 (C-16);

26.27 (C-4); 24.00 (C-15); 21.14 (C-11); 17.27 (C-21); 13.54 (C-19); 12.06 (C-18). HRMS-ESI (positive mode): m/z calculated for $C_{29}H_{39}O_5I$: 594.1842 $[M]^+$; found 617.1732 $[M + Na]^+$.

2α,3α-Dihydroxy-5α-cholan-6-oxo-23,24-dinor-22-yl 4-cyanobenzoate (**22**)
Olefin **28** (0.43 g, 0.93 mmol); DHQD-CLB (0.14 g, 0.30 mmol); $CH_3SO_2NH_2$ (0.24 g, 2.52 mmol); K_2CO_3 (0.88 g, 6.37 mmol); $K_3[Fe(CN)_6]$ (2.14 g, 6.50 mmol); OsO_4 (0.63 mL, 0.12 mmol). Compound **22** is obtained as a colorless solid (0.29 g, 0.59 mmol, 63.4% yield) (m.p. = 218.8–220.3 °C). IR_{vmax} (cm^{-1}): 3483 (O-H); 2943 (CH_3-); 2865 (CH_2-); 2200(CN); 1722 (C=O); 1697 (COO); 1446 (CH_2-); 1390 (CH_3-); 1108 and 1047 (C-O). ^{1}H NMR (400.1 MHz, $CDCl_3$): δ (ppm) = 8.12 (2H, d, *J* = 8.5 Hz, H-2´); 7.74 (2H, d, *J* = 8.5 Hz, H-3´); 4.34 (1H, dd, *J* = 10.8 and 3.3 Hz, H-22a); 4.07 (1H, dd, *J* = 10.8 and 7.4 Hz, H-22b); 4.03 (1H, bs, H-2); 3.75 (1H, dt, *J* = 10.6 Hz, H-3); 2.67 (1H, dd, *J* = 12.3 and 2.5 Hz, H-5); 2.29 (1H, dd, *J* = 13.1 and 4.5 Hz, H-7a); 2.15 (2H, bs, 2 x OH); 1.17-1.07 (1H, m, H-15); 1.11 (3H, d, *J* = 6.6 Hz, H-21); 0.75 (3H, s, H-19); 0.71 (3H, s, H-18). ^{13}C NMR (100.6 MHz, $CDCl_3$): δ (ppm) = 212.00 (C-6); 165.01 (ArCOO); 134.22 (CN); 132.21 (C-3´); 129.98 (C-2´); 117.95 (C-4´); 116.24 (C-1´); 70.62 (C-22); 68.30 (C-3); 68.18 (C-2); 56.27 (C-14); 53.57 (C-9); 52.68 (C-17); 50.68 (C-5); 46.62 (C-7); 43.12 (C-13); 42.48 (C-10); 40.09 (C-1); 39.19 (C-12); 37.59 (C-8); 35.85 (C-20); 27.51 (C-16); 26.25 (C-4); 23.97 (C-15); 21.11 (C-11); 17.23 (C-21); 13.51 (C-19); 12.04 (C-18). HRMS-ESI (positive mode): m/z calculated for $C_{30}H_{39}O_5N$: 493.2828 $[M]^+$; found 516.2718 $[M + Na]^+$.

Biological assays

Rice lamina inclination test (RLIT).

All synthesized BR analogues (**12**, **14** and **17**–**22**) were assayed by RLIT according to a modified procedure described previously [2,3]. Seeds of a local Rice cultivar (*Oryza sativa* L, variety Sapphire) provided by INIA-QUILAMAPU, Chile were sown and grown for approximately 10 days in pots with substrate (peat, perlite, and vermiculite in a 2:1:1 ratio (v/v/v)) under controlled conditions in a plant growth chamber (22 °C, 16 h light/8 h dark photoperiod, and 50% relative humidity). Subsequently, uniformly growing rice plants that presented the second internode of the rice lamina were selected to cut an approximately 8-cm segment; these segments were placed in a Petri dish containing sterile distilled water (50 mL) and BR analogues or brassinolide (APExBIO Technology LLC, Houston, Texas, USA) as positive control at different concentrations (1×10^{-8}; 1×10^{-7} and 1×10^{-6} M). The negative control included sterile distilled water (SDW) and dimethyl sulfoxide (DMSO, Sisco Research Laboratories Pvt. Ltd. India) at 0.002 vol %, which is used as solvent to dilute BRs analogues solutions. The segments are incubated for 72 h, at 25 °C, in the dark and the angle of inclination between the leaf and sheath is measured. Each treatment consists of 8–10 independent replicates; significant differences between the positive control and treatments are evaluated with these data. Images are taken with a Leica EZ4HD stereo microscope (Wetzlar and Mannheim; Germany) with camera software.

Root growth inhibition assays in *Arabidopsis thaliana* seedlings.

Arabidopsis thaliana L. (Heyhn.) seeds (ecotype Columbia, Col-0; referred to *Arabidopsis* (Arabidopsis Biological Resource Center, Phoenix Bioinformatics

Corporation (TAIR), USA) are sterilized, stratified for 3 days at 4 °C, and grown vertically in Murashige and Skoog growth medium (1/2 MS) supplemented with 1 wt % sucrose. Brassinolide or different BRs analogues (**12**, **14** and **17**–**22**) are added, reaching a final concentration of 1×10^{-6} M. DMSO is used as negative control at a final concentration of 0.1 vol %. The germinated seeds are then transferred to a plant growth chamber in an upright position at 22 °C in a 16 h/8 h light–dark cycle for 5 days to subsequently evaluate root growth. Finally, root lengths are photographed for measurement with ImageJ/FIJI 1.46r image processing software (National Institutes of Health) (http://rsbweb.nih.gov/ij/; accessed May 30, 2023). For each treatment, 20 seedlings (replicates) are analyzed [4].

Western blots analysis

For immunological analysis of BES1 protein levels, experiments have been performed according to Vukasinovic et al. [5]. with some modifications. For protein extraction, approximately 25 roots are collected from 6-day-old Col-0 seedlings. Plant material is frozen in liquid nitrogen, crushed with the Retsch MM400 and homogenized in 100 µL of cold homogenization buffer (1% v/v SDS, 25mM Tris/HCl pH 7.5, 150 mM NaCl, 10mM DTT and Roche complete protease inhibitor (one tablet/ 10 mL) and placed on ice for 30 min. Homogenates are centrifuged twice (10 min,14,000 rpm) at 4 °C. After addition of 4 × lithium dodecyl sulphate (LDS) and reducing agent (10×), samples are heated for 10 min at 70 °C, centrifuged again and separated on unstained polyacrylamide electrophoresis gel (4–15 vol %, Bio-Rad Laboratories) and blotted into PVDF membranes using Trans-Blot Turbo Mini PVDF Transfer Packs. Membranes are blocked at 4 °C in Difco 5 vol % skimmed milk. Immunodetection of BES-1 is performed with rabbit polyclonal anti-BES1 antibody

(R3489-2; Abiocode, California, USA) diluted 1:1000 in Difco 5 vol % skimmed milk as primary antibody for 3 h at 37 °C followed by 3 washes with TBS-T 1X for 5 min each and incubation with a HRP conjugated anti-rabbit antibody (Merck) diluted 1:10,000 in Difco 5 vol % skimmed milk for 1 h at 37 °C as secondary antibody. Tubulin was used as a normalizer, and its detection is performed with an anti-Tubulin antibody (Abcam) diluted 1:5000 in Difco 5 vol % skimmed milk and anti-sheep mouse (Merck) diluted 1:10000 in Difco 5 vol % skimmed milk as secondary antibody. Secondary antibodies are developed with Clarity Western ECL substrate (BIO-RAD, USA) following the manufacturer instructions and signal from labelled protein bands is analysed with the ChemiDoc MP imaging system. For BES1, the ratio of dephosphorylated BES1 proteins to total BES1 is quantified according to signal intensity. The loading is adjusted to an equal level based on the amount of tubulin. Signal intensities are determined with Image software Lab.

Molecular docking

The crystallographic structure of Brassinosteroid Insensitive 1 (BRI1) in complex with BRI1-Associated Receptor Kinase 1 (BAK1) and the natural ligand brassinolide (BLD) (PDB ID: 4M7E), resolved at 3.60 Å, was retrieved from the Protein Data Bank (http://www.rcsb.org/). The structure is optimized using pdb2pqr (v. 3.6.0), accessed on November 17, 2025 through the PDB2PQR web server (https://server.poissonboltzmann.org/pdb2pqr), and processed with the AMBER force field. The protonation states of ionizable groups at pH 8 were assigned using PROPKA 4.5 [6,7]. For each analyzed compound, a molecular model was generated from its SMILES string using UCSF Chimera [8]. Energy minimization of the models

was performed under Chimera's default conditions using MMTK and Antechamber parameters [9].

AutoDock Vina 1.1.2 was employed as the docking algorithm, using a grid box of 20 × 20 × 20 Å centered on the BLD coordinates in the crystal structure. For each molecular model, five docking runs were conducted, generating ten poses per run, with an exhaustiveness value of eight. The maximum energy difference between the modes was set to 2 kcal/mol. Docking results were evaluated based on binding affinity (kcal/mol) as well as hydrogen-bonding, hydrophobic, and electrostatic interactions. Following docking, comparisons were made between each docked ligand and the re-docked BLD poses. Visualization of the docked poses for analysis was performed using UCSF Chimera [X3] and Discovery Studio Visualizer (Dassault Systemes BIOVIA, San Diego, CA, USA).

References

1. Aitken, V.; Diaz, K.; Soto, M.; Olea, A. F.; Cuellar, M. A.; Nuñez, M.; Espinoza-Catalán, L. *Int. J. Mol. Sci.* **2024,** *25,* 419.
2. Yin, Y.; Wang, Z.-Y.; Mora-Garcia, S.; Li, J.; Yoshida, S.; Asami, T.; Chory, J. *Cell* **2002**, *109* (2), 181–191.
3. Diaz, K.; Espinoza, L.; Carvajal, R.; Conde-Gonzalez, M.; Niebla, V.; Olea, A. F.; Coll, Y. *Int. J. Mol. Sci.* **2020**, *21* (5), 1832.
4. Nuñez, M.; Wang, Y.; Russinova, E.; Estévez-Braun, A.; Amesty, A.; Olea, A. F.; Mellado, M.; Díaz, K.; Espinoza Catalán, L. *Int. J. Mol. Sci.* **2024**, *25* (18), 10158.

5. Vukašinović, N.; Wang, Y.; Vanhoutte, I.; Fendrych, M.; Guo, B.; Kvasnica, M.; Jiroutová, P.; Oklestkova, J.; Strnad, M.; Russinova, E. *Nature Plants* **2021**, *7* (5), 619–632.
6. Olsson, M. H. M.; Søndergaard, C. R.; Rostkowski, M.; Jensen, J. H. *J. Chem. Theory Comput.* **2011**, *7* (2), 525–537.
7. Søndergaard, C. R.; Olsson, M. H. M.; Rostkowski, M.; Jensen, J. H. *J. Chem. Theory Comput.* **2011**, *7* (7), 2284–2295.
8. Pettersen, E. F.; Goddard, T. D.; Huang, C. C.; Couch, G. S.; Greenblatt, D. M.; Meng, E. C.; Ferrin, T. E. *J Comput Chem* **2004**, *25* (13), 1605–12.
9. Wang, J.; Wang, W.; Kollman, P. A.; Case, D. A. *J Mol Graph Model* **2006**, *25* (2), 247–60.
